# Protein modification in neurodegenerative diseases

**DOI:** 10.1002/mco2.674

**Published:** 2024-08-04

**Authors:** Shahin Ramazi, Maedeh Dadzadi, Mona Darvazi, Nasrin Seddigh, Abdollah Allahverdi

**Affiliations:** ^1^ Department of Biophysics Faculty of Biological Sciences Tarbiat Modares University Tehran Iran; ^2^ Department of Biotechnology Faculty of Advanced Science and Technology Tehran Medical Sciences Islamic Azad University Tehran Iran; ^3^ Department of Biochemistry Faculty of Advanced Science and Technology Tehran Medical Sciences Islamic Azad University Tehran Iran

**Keywords:** immune system, neurodegenerative diseases, posttranslational modifications, SUMOylation

## Abstract

Posttranslational modifications play a crucial role in governing cellular functions and protein behavior. Researchers have implicated dysregulated posttranslational modifications in protein misfolding, which results in cytotoxicity, particularly in neurodegenerative diseases such as Alzheimer disease, Parkinson disease, and Huntington disease. These aberrant posttranslational modifications cause proteins to gather in certain parts of the brain that are linked to the development of the diseases. This leads to neuronal dysfunction and the start of neurodegenerative disease symptoms. Cognitive decline and neurological impairments commonly manifest in neurodegenerative disease patients, underscoring the urgency of comprehending the posttranslational modifications’ impact on protein function for targeted therapeutic interventions. This review elucidates the critical link between neurodegenerative diseases and specific posttranslational modifications, focusing on Tau, APP, α‐synuclein, Huntingtin protein, Parkin, DJ‐1, and Drp1. By delineating the prominent aberrant posttranslational modifications within Alzheimer disease, Parkinson disease, and Huntington disease, the review underscores the significance of understanding the interplay among these modifications. Emphasizing 10 key abnormal posttranslational modifications, this study aims to provide a comprehensive framework for investigating neurodegenerative diseases holistically. The insights presented herein shed light on potential therapeutic avenues aimed at modulating posttranslational modifications to mitigate protein aggregation and retard neurodegenerative disease progression.

## Graphical abstract

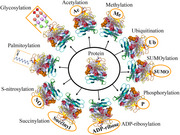

## 1 INTRODUCTION

**FIGURE 1:**
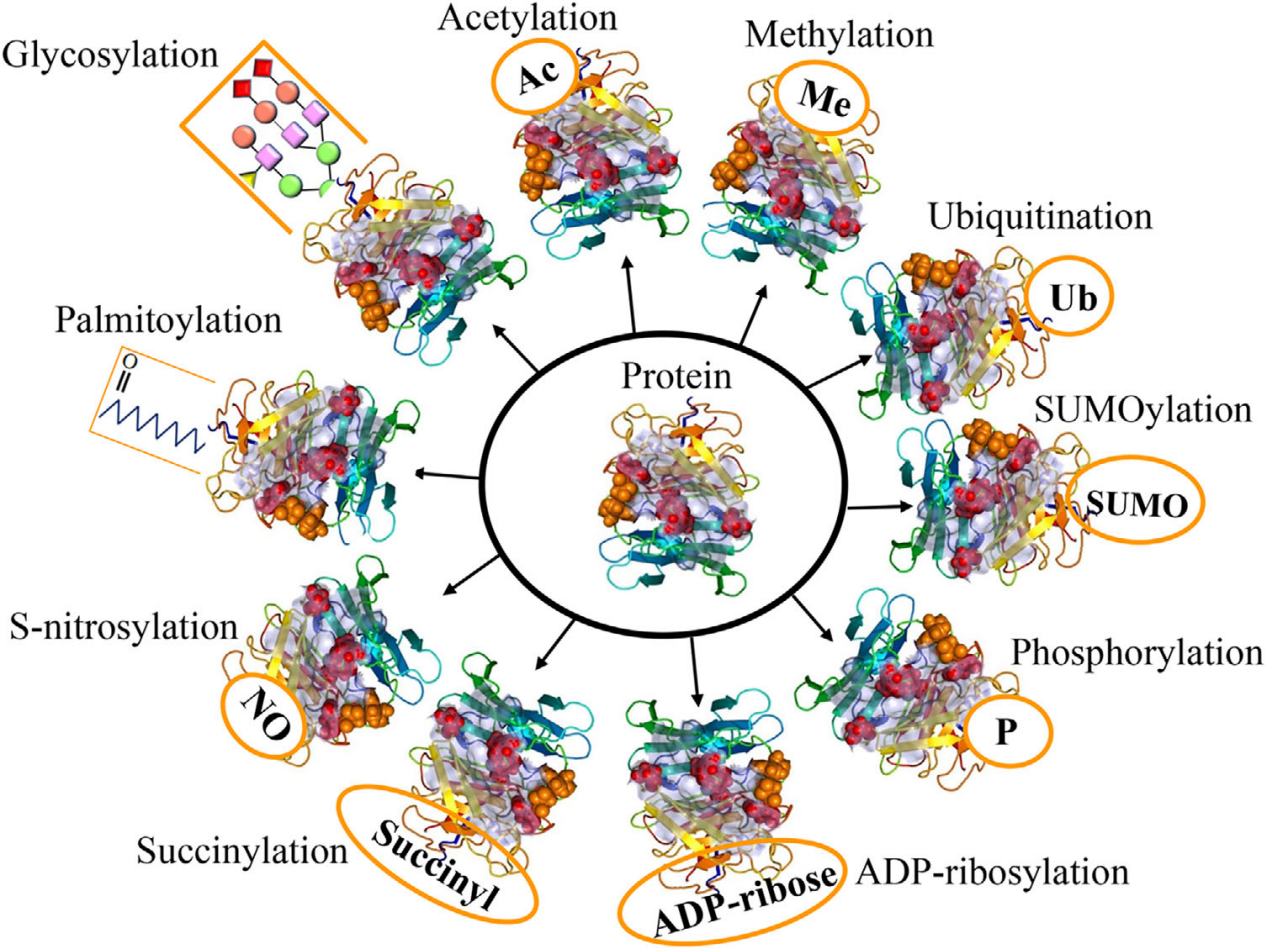
The graphical abstract showcases the diversity of posttranslational modifications (PTMs) influencing protein structure and function. It features schematic representations of the following 10 prominent PTMs: phosphorylation (addition of phosphate groups), acetylation (addition of acetyl groups), methylation (addition of methyl groups), SUMOylation (attachment of SUMO proteins), ubiquitylation (attachment of ubiquitin molecules), succinylation (addition of succinyl groups), S‐nitrosylation (attachment of NO), ADP‐ribosylation (addition of ADP‐ribose groups), glycosylation (addition of sugar molecules), and palmitoylation (attachment of palmitate groups).

Posttranslational modifications (PTMs) are crucial for regulating protein function, stability, and cellular processes. These modifications, which occur after protein synthesis, fine‐tune protein activities and enable dynamic responses to physiological conditions.^1^ The dynamic nature of PTMs allows for the addition, removal, or alteration of chemical groups on specific amino acid residues within a protein. This intricate network of modifications expands the functional repertoire of proteins beyond their primary amino acid sequence.^2^ PTMs play a crucial role in regulating cellular processes, signaling pathways, and regulatory networks by modulating protein interactions, enzymatic activities, subcellular localization, and stability. PTMs involve covalent modifications that can be either enzymatic or nonenzymatic. These modifications can occur on amino acid side chains or protein termini, introducing novel functional groups or modifying existing ones.^3^, ^4^ By modulating protein interactions, enzymatic activities, subcellular localization, and stability, PTMs contribute to the complexity of cellular processes, signaling pathways, and regulatory networks. PTMs involve covalent modifications that can be enzymatic or nonenzymatic. These modifications can attach to amino acid side chains or protein termini, introducing novel functional groups or modifying existing ones (Figure 1).^5^ The most common type of PTM is phosphorylation, but there are many other types, such as acetylation, methylation, ubiquitination, glycosylation, and proteolysis. PTMs occur within diverse cellular compartments, such as the endoplasmic reticulum (ER) and the Golgi apparatus. The association of PTM malfunctions with a wide range of developmental disorders and human diseases underscores the critical role played by PTMs in preserving the equilibrium of cellular states. PTMs are essential for maintaining cellular homeostasis and responding to environmental stimuli. However, aberrant PTMs can also contribute to the development and progression of various diseases, such as cancer, neurodegeneration, inflammation, and infection.^3^, ^4^ Recent studies have reported the existence of over 620 types of PTMs, which can involve proteolytic degradation or the covalent attachment of functional groups such as acetyl, phosphoryl, glycosyl, methyl, and others.^6^, ^7^ The experimental methods and information obtained from mass spectrometry (MS) are used to identify the different types of PTMs and their positions.^1^ Imbalances in the regulation of PTMs of proteins are known to play a crucial role in the development of neurodegenerative diseases (NDDs) such as Alzheimer's disease (AD), Parkinson disease (PD), and Huntington's disease (HD).^8^ Disruptions in PTMs and protein quality control mechanisms, including molecular chaperones, the ubiquitin–proteasome system (UPS), and the autophagy–lysosomal degradation pathway, can lead to the accumulation of misfolded proteins, ultimately leading to impaired neuronal function. Aberrant PTMs can exert a profound influence on the propensity of proteins to aggregate, a defining characteristic of NDDs. PTMs have a significant impact on the activity, stability, and clearance of proteins, thereby influencing key neurodegenerative processes. Extensive research has elucidated the critical role of PTMs, especially in proteins such as Tau, in modulating protein function, degradation, and aggregation. These findings underscore the importance of PTMs in the context of NDDs. Numerous studies have provided insights into the dysregulation of diverse PTMs, such as phosphorylation, ubiquitination, O‐GlcNAcylation, acetylation, methylation, and glycosylation, in the pathogenesis of NDDs.^9^ Perturbations in these PTMs can have significant implications for the initiation and advancement of these conditions. Hence, the identification and investigation of aberrant PTMs and their underlying mechanisms in NDDs present a promising avenue for enhancing our comprehension of disease progression and facilitating the development of novel treatment and prevention approaches.

This review delves into the intricate roles of 10 abnormal PTMs: phosphorylation, acetylation, methylation, SUMOylation, ubiquitylation, succinylation, S‐nitrosylation, glycosylation, ADP‐ribosylation (ADPR), and palmitoylation in NDDs. By scrutinizing the impact of these PTMs on NDD pathogenesis, our objective is to enrich our comprehension of how these modifications drive disease progression and pinpoint potential therapeutic targets. This article endeavors to provide a comprehensive overview of the current research landscape on PTMs in NDDs, aiming to catalyze further investigations into the complexities of PTMs and their implications in the realm of NDDs.^10^

## 2 EFFECTS OF ABNORMAL SUMOYLATION OF PROTEINS IN NDDs

Small ubiquitin‐related modifiers (SUMO) and other ubiquitin‐like proteins (Ubls) have been identified as important regulators with a variety of biological roles among these modifiers.^11^ SUMO proteins covalently and reversibly conjugate to specific lysine (Lys) residues in target proteins.^12^ The SUMO protein was primarily discovered by Mahajan in 1996 in the context of the Ran GTPase‐activating protein.^13^ SUMOylation is a process that occurs at specific Lys residues in target proteins through SUMO conjugation.^14^ SUMO is a small protein with a molecular weight of about 11 kDa and a length of 96 amino acids that exhibits a three‐dimensional structure similar to ubiquitin.^15^ Furthermore, SUMOylation is essential for nearly all eukaryotic organisms and occurs in a large number of cells.^16^ This modification primarily occurs on protein substrates, including nucleoproteins, cytoplasmic proteins, and membrane proteins.^17^ SUMOylation is highly conserved and is central to the regulation of various cellular processes, with significant effects on the stability of the modified proteins.^10^ Moreover, this modification is an important mechanism in cellular processes such as regulation of the cell cycle, cellular stress responses, genome stability, DNA repair, and cell viability,^18^, ^19^ metabolism, regulation of DNA replication, mRNA transcription, apoptosis, intracellular transport, protein transport, activation and deactivation of the acetylation process, chromosome separation, consolidation of chromatin structure, and mitotic division.^20^, ^21^ However, many of the functional outcomes of SUMOylation remain unknown.^22^, ^23^ Defects in the SUMOylation pathway, as well as an imbalance between SUMOylation and deSUMOylation, have been associated with the occurrence and progression of various diseases, such as cancer, heart failure, diabetes, brain stroke, and brain failure.^3^, ^24^ Additionally, SUMOylation has emerged as a factor in several NDDs, such as HD, PD, and AD.^3^

**FIGURE 2:**
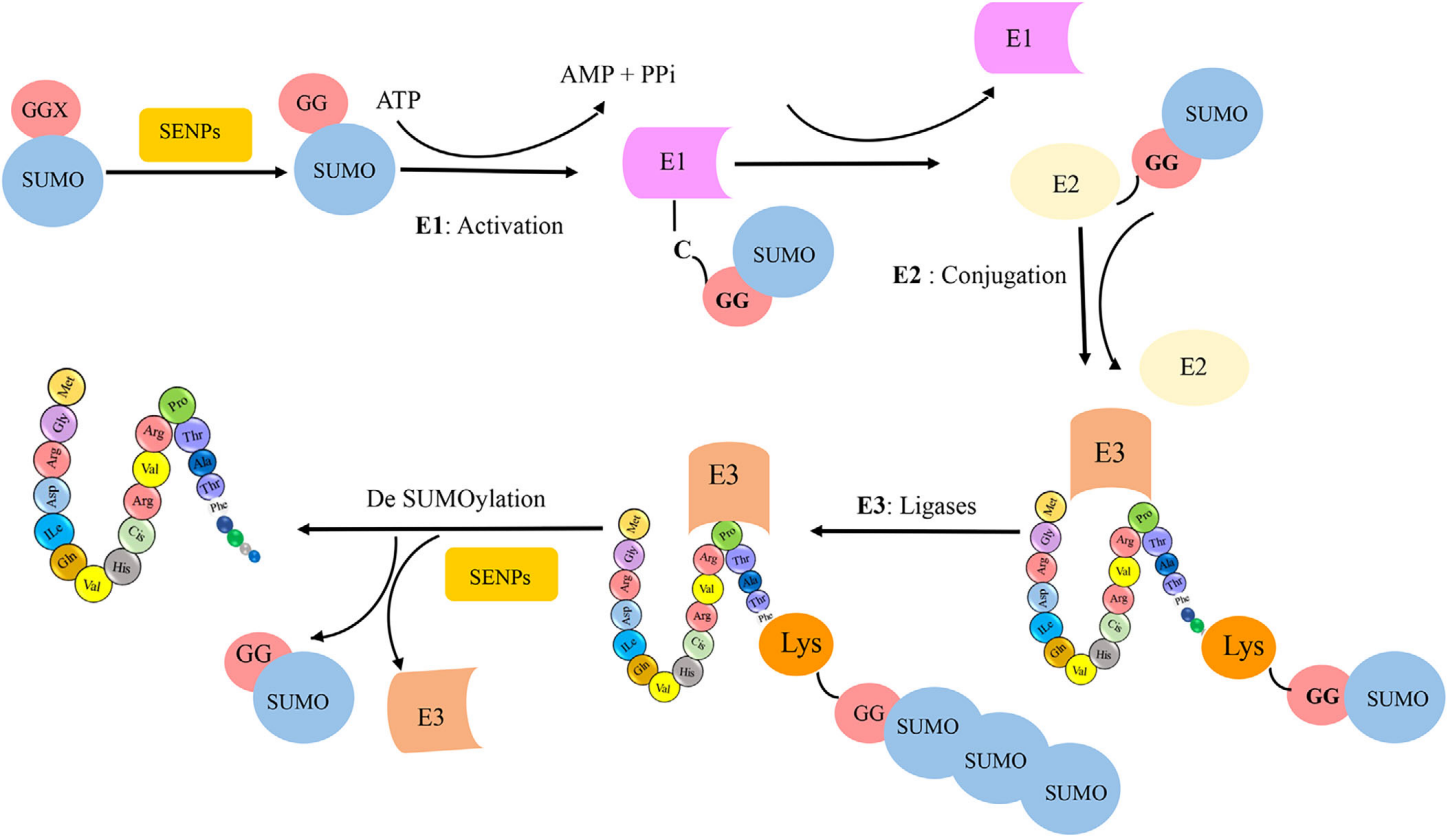
SUMOylation is a reversible modification in which SUMO is covalently attached to proteins through an enzymatic cascade involving three enzymes: an activating enzyme (E1), a conjugating enzyme (E2), and a ligating enzyme (E3). Ultimately, SUMO is removed by a sumo‐specific protease.

SUMO proteins have been found to interact with a wide range of enzymatic families.^25^ Members of the SUMO family can alter the biochemical properties of their target proteins by binding reversibly to them.^26^, ^27^ SUMO has been identified under various names, such as smt3p, pmt2, PIC‐1, GMP‐1, Ubl1, and Sentrin.^17^, ^28^ Specifically, the SUMO family has three isoforms in mammals, four in humans, one in yeasts, and eight in plants.^14^ In most vertebrates, the SUMO family has three isoforms, known as SUMO‐1 (also called Sentrin, PIC1, GMP1, Ubl1, Smt3c), SUMO‐2 (also called Sentrin‐2, Smt3b), and SUMO‐3 (also called Sentrin‐3, SMT3B).^29^ These isoforms are involved in different cellular mechanisms.^30^ SUMO‐2 and SUMO‐3 are highly similar to each other than to SUMO‐1, sharing 97% identity. Notably, currently available antibodies cannot distinguish between SUMO‐2 and SUMO‐3 due to their high sequence similarity. In contrast, SUMO1 shares only 47% sequence identity with SUMO‐2/3.^31^ SUMOylation is a reversible PTM involving the covalent attachment of SUMO proteins to target proteins. This process shares a remarkable resemblance with the ubiquitylation pathway. It is mediated by an enzymatic cascade comprised of three essential enzymes: an activating enzyme (E1, SAE1/SAE2 heterodimer), a conjugating enzyme (E2, UbC9), and a ligating enzyme (E3). Ultimately, SUMO is enzymatically cleaved by a sumo‐specific protease (see Figure 2). The N‐terminal of the SUMO protein contains GGX amino acids, where X can be any amino acid.^3^ During SUMOylation, the SUMO protein connects with glycine via a covalent bond to Lys in a substrate. Additionally, the SUMO family binds to proteins via noncovalent bonds and creates a motif for binding other proteins, known as SUMO‐interacting motifs.^32^

Proteins involve a multitude of Lys residues, but only a specific subset of distinct Lys residues serves as a site for SUMOylation.^22^, ^33^ SUMO substrates contain both consensus and nonconsensus sites.^34^ Recent proteomics studies suggest that a considerable amount of SUMOylation occurs on nonconsensus sites.^10^ However, consensus motifs in SUMOylated proteins have contained an acceptor Lys within a (ΨKXD/E) where Ψ is a hydrophobic residue (e.g., I, V, L, A, P, or M), X is any residue, and D/E is an acidic residue.^35^ SUMOylation sites can also be found in extended motifs that contain a SUMOylation negatively charged amino acid motif (NDSM: WKXE (D/E), a SUMOylation dependent phosphorylation motif (PDSM: WKXEXXSP), and a SUMO‐style motif (WKXEP).^19^ These results imply that the core motif's flanking sequence (WKXE) might possibly be involved in the particular recognition of SUMOylation sites. Additionally, it is noteworthy that some SUMOylation sites do not follow the above motifs and are called nonconsensus motifs. However, analysis and experimental data have shown that approximately 23% of sites do not follow this consensus motif. SUMOylation motifs typically include the target Lys with an equal number of residues in the upstream and downstream regions, which play a crucial role in the reaction at these sites. The amino acids surrounding the target Lys greatly influence the site's biochemical properties.^11^, ^12^

NDDs are significant contributors to disability in aging societies, primarily due to the progressive deterioration of neuronal function, resulting in brain atrophy.^13^ These disorders constitute a broad category of neurological diseases that affect the central nervous system (CNS), peripheral nervous system, and autonomic nervous system. These disorders are characterized by area‐specific extensive neuronal loss, leading to sensory, motor, consciousness, and autonomic dysfunction.^36^, ^37^ The genesis of neurodegenerative disorders is frequently attributed to misfolded or clumped proteins.^16^ Diverse PTMs, such as phosphorylation, SUMOylation, and others, heavily modify neuronal proteins involved in cell signaling pathways. Previous studies suggest that SUMOylation may be a generic mechanism for regulating phosphorylation dynamics.^14^, ^15^ Various PTM types play essential roles in the intricate synaptic connection that occurs between neurons in the CNS, facilitating swift, efficient, and reversible reactions to environmental inputs.^14^ Conversely, disruption of PTMs in neuronal proteins can lead to neurological disorders associated with various NDDs.^15^ Any aggregation of proteins can be toxic to neurons and significantly contribute to neuronal death.^16^

Some NDDs are associated with protein SUMOylation disorders, which have variable effects on key proteins involved in disease pathogenesis. Thus, regulating protein SUMOylation in neurons might present a novel approach to creating therapeutic medications with specific targets for neurodegenerative illnesses.^36^, ^38^ SUMOylation plays a crucial role in the regulation of intracellular signaling and maintains genome integrity, and its dysfunction has been linked to cancer, ischemia, NDDs, diabetes, and heart failure. Consequently, SUMOylation inhibitors are currently in development for clinical applications.^39^ Although SUMOylation plays several roles in the brain, its role in neuroinflammation is relatively new, and limited research has been conducted concerning glial SUMOylation. The SUMOylation of glial cells under pathological conditions appears to affect relevant cellular processes. Whether SUMOylation exhibits pro‐ or anti‐inflammatory activity depends on the conjugated SUMO isoform, the target protein, and the type of cell.^36^ The critical aspect of neurodegenerative disorders is neuroinflammation, in which glial cells such as astrocytes and microglia play key roles. The proper functioning of glial cells is critical in a healthy CNS, and their dysfunction is associated with gliosis. The role of SUMOylation, with its pro‐ or anti‐inflammatory actions, in neuroinflammation is a subject of much interest. Growing evidence suggests that loss of the SUMOylation pathway in astrocytes and microglial cells can impact inflammatory conditions relevant to pathophysiological processes. Nitric oxide synthase type 2 (NOS_2_) is known for its involvement in various neurological disorders through the generation of nitric oxide (NO). NOS_2_ is induced in primary astrocytes under inflammatory conditions and is also implicated in neurological disorders such as multiple sclerosis (MS), cerebral ischemia, and AD. The expression of NOS_2_ is regulated by noradrenaline (NA), an anti‐inflammatory neurotransmitter. Notably, NA inhibits the inflammatory induction of NOS_2_ in astrocytes. Additionally, the upregulation of SUMO‐1, ubc9, and SENP1 has been found to reduce the activity of the NOS_2_ promoter. Moreover, normal aging and proinflammatory conditions induced by lipopolysaccharide (LPS) treatment have been shown to decrease the expression of SUMO‐1, ubc9, and SENP1 in primary astrocytes. These findings suggest that SUMO‐1 may possess an anti‐inflammatory role. Importantly, NA mitigates the effects of LPS on SUMO‐1, ubc9, and SENP1, leading to an increase in SUMO‐1 levels. Consequently, SUMO‐1 may contribute to the anti‐inflammatory effects mediated by NA in the context of NDDs.^40^ The synthetic liver X receptor (LXR) α and LXR β, both ligand‐activated nuclear receptors, are involved in regulating immune and inflammatory responses. In the context of AD, the downregulation of LXR α and LXR β expression in mouse models contributes to the development of senile plaques (SP). This loss of LXRs results in an inflammatory response from glial cells towards β‐amyloid fibrils (fAβ), contributing to the pathology observed in AD. LXR ligands have been shown to inhibit the binding of signal transducer and activator of transcription 1 (STAT1) to the promoters of IRF1, TNFα, and IL‐6, thereby preventing their expression. Notably, investigations have revealed that SUMOylation of LXRs plays a crucial role in inhibiting STAT1 binding. When LXRs are SUMOylated, they form a complex with STAT1, preventing the binding of STAT1 to the promoter regions of target genes. As a result, the transcription of these target genes fails to occur. This highlights the importance of SUMOylation in the anti‐inflammatory function of LXRs, as it suppresses the STAT1‐dependent inflammatory response in interferon‐gamma (IFN‐γ)‐stimulated brain astrocytes.^41^ Promyelocytic leukemia (PML) is an IFN‐stimulated gene (ISG) with a promoter that contains IFN‐stimulated response elements. PML is known to form unique structures called PML nuclear bodies (PML‐NBs) within the nucleus, which are associated with the nuclear matrix. These PML‐NBs respond to various stressors, particularly those related to viral infections. In interferonopathy models and in microglia associated with amyloid‐β (Aβ) plaques in AD, there is an upregulation of PML expression. This suggests that PML plays a role in the immune response within microglia and is involved in the pathogenesis of AD.^42^ Overexpression of nuclear PML isoforms has been observed to enhance the phosphorylation of STAT1, increase its DNA binding activity, and promote the expression of ISGs in response to IFNγ stimulation. On the other hand, silencing PML leads to a reduction in IFNγ‐induced STAT1 activation. Importantly, SUMOylation of PML is crucial for promoting STAT1 activity and the overall global SUMOylation in response to IFNγ treatment. Additionally, PML deletion has been shown to decrease SUMOylation levels in primary bone marrow‐derived macrophage (BMDM) cultured cells. These findings highlight the role of PML SUMOylation in regulating STAT1 activity and the broader SUMOylation response in the context of IFNγ signaling. Thus, SUMOylation seems to play a role in inflammatory PML. Interestingly, arsenic trioxide (ATO), an FDA‐approved drug against acute PML, leads to the prevention of STAT1 phosphorylation. The inhibitory impact of ATO on STAT1 is partially dependent on PML, as PML deletion and ATO pretreatment similarly inhibit inflammation resulting from IFN in the CNS: in both models in microglia under polyinosinic–polycytidylic acid (PI:C) stimulation, there have been lower levels of CD68 lysosomal markers and ISG transcription. ATO inhibits the overall SUMOylation upon PI:C and prevents the PI:C‐induced inflammation in microglia, suggesting that ATO can be an anti‐inflammatory agent by suppressing SUMOylation in the context of CNS diseases correlated with IFN signaling.^42^ Astrocyte reactivity is known to play a role in the pathogenesis of NDDs, and it is increased in mouse models as well as in patients with AD. This increased reactivity is characterized by enhanced expression of glial fibrillary acidic protein (GFAP), an intermediate filament protein, and morphological changes in astrocytes. In AD, the presence of Aβ peptides contributes to the activation of astrocytes and their reactive phenotype. Furthermore, Aβ‐treated reactive astrocytes have been found to be associated with an inflammatory response, which can exacerbate neurotoxicity in the surrounding environment. However, curcumin, a naturally occurring compound with anti‐inflammatory and antioxidant properties, has been shown to modulate astrocyte properties and reduce astrocyte reactivity. Curcumin has been investigated for its potential therapeutic effects in NDDs, including its ability to attenuate astrocyte‐mediated inflammation and neurotoxicity.^43^ Indeed, curcumin has been found to reduce the enhanced expression of GFAP and morphological changes induced by Aβ exposure. Additionally, it is worth noting that upon Aβ exposure, the conjugation of proteins by SUMO‐1 is significantly decreased, while SUMO‐2/3 conjugation remains unaffected. In astrocytes, increasing the SUMOylation of proteins, through the overexpression of SUMO‐1GG (a mutant form of SUMO‐1 that promotes SUMOylation), leads to a decrease in elevated GFAP levels caused by Aβ. This suggests that SUMO‐1 may play a protective role against astrocyte reactivity in the context of Aβ‐induced pathology. Research has indeed demonstrated that curcumin has the ability to inhibit the Aβ‐induced reduction in SUMO‐1 conjugation in astrocytes. Furthermore, curcumin has been shown to inhibit the Aβ‐induced activation of JNK, which is a crucial kinase involved in cellular stress responses. These findings suggest that astrocytes may rely on SUMO‐1 conjugation to maintain a nonreactive state, and higher levels of SUMO‐1 conjugation in astrocytes may have a neuroprotective role. The ability of curcumin to preserve SUMO‐1 conjugation and inhibit JNK activation underscores its potential as a therapeutic agent for modulating astrocyte reactivity and promoting neuroprotection in the context of Aβ‐induced pathology.^43^

There is now clear evidence that nuclear and extranuclear proteins can be SUMOylated in both neuropathological and normal states.^14^, ^25^ The impact of SUMOylation extends beyond nuclear functions and encompasses a wide range of processes, including neuronal development, stress responses, synaptic transmission, and plasticity. By modifying target proteins, SUMOylation plays a crucial role in fine‐tuning the activity and interactions of proteins involved in these neuronal processes. Overall, SUMOylation serves as an important regulatory mechanism in the nervous system, influencing a diverse array of cellular functions and contributing to the maintenance of neuronal homeostasis and proper brain function.^44^ This regulation applies to both nuclear and extranuclear proteins, exerting influence over a diverse array of functions. The binding of SUMO to proteins located outside the nucleus encompasses a wide range of extranuclear activities. These include autophagy, exocytosis, the regulation of G‐protein signaling, modulation of mitochondrial dynamics, enzyme activity, adjustments in channel activity, modulation of receptor function, cytoskeletal functions, mRNA trafficking, and regulation of phosphorylation.^14^, ^45^ Disruptions in SUMOylation can profoundly affect nuclear function, thereby contributing to neuronal dysfunction and altered synaptic plasticity, both of which are vital for maintaining optimal nervous system performance. Impairments in nuclear function resulting from SUMOylation disruption can engender neuronal stress responses and perturb synaptic plasticity. Consequently, gaining a comprehensive understanding of the intricate interactions between SUMOylation and its influence on nuclear function is essential for comprehending the underlying mechanisms associated with brain health and disorders.^44^

Within the neuronal nucleus, both SUMOylation and deSUMOylation enzymes, along with their target proteins, are highly concentrated and serve essential roles in maintaining neuronal viability, nucleocytoplasmic transport, maturation, and differentiation.^25^ The precise equilibrium between SUMOylation and deSUMOylation can be disrupted by environmental and metabolic stressors, leading to the regulation of these processes in response to synaptic activity. Moreover, evidence suggests that extranuclear SUMOylated proteins can impact synaptic function.^25^, ^27^ The role of SUMOylation in crucial neuronal pathways within axons and synapses, such as axonal trafficking and guidance, as well as synapse formation and synaptic transmission, has been established.^46^ As a result, the SUMO substrates can also be found localized in presynaptic regions, excitatory postsynapse regions, and inhibitory postsynapse regions at synapses, highlighting the significance of SUMOylation and deSUMOylation in shaping synaptic function.^25^ For instance, local protein synthesis in neurons involves various processes such as synapse formation and synaptic plasticity and also relies on mRNA transport via mRNA‐binding proteins. La protein, as a multifunctional RNA‐binding protein, is one such protein regulated by SUMOylation, which determines the direction of its axonal transport by interacting with the motor proteins kinesin and dynein. SUMOylated La binds exclusively to dynein, while non‐SUMOylated La binds only to kinesin, determining the transport direction of La and associated mRNAs. Other mRNA‐binding proteins may also be subject to directional regulation, highlighting the importance of SUMOylation in mRNA delivery within neurons.^47^ SUMOylation's role in synaptic function and signaling is underlined by its impact on some presynaptic proteins, such as synaptotagmin‐1.^46^ Notably, it has been observed that SUMO‐1, SUMO‐2/3, and Ubc9 predominantly reside within the nucleus. However, there is evidence indicating their partial colocalization with pre‐ and postsynaptic markers, including synaptophysin and postsynaptic density protein 95 (PSD‐95). The localization of SUMO‐1, SUMO‐2/3, and Ubc9 with synaptic markers PSD95 and synaptophysin strongly suggests that SUMO proteins are present at the synapse, providing valuable insights into their potential roles in synaptic function and plasticity.^48^

High levels of SUMO‐1 and SUMO‐2/3 expression have been found in the brain.^28^, ^29^ Studies have substantiated the indispensable contribution of SUMO‐1 to the development and functioning of neurons.^46^ Accordingly, SUMO‐1 is observed to modify Tau and α‐synuclein (α‐syn), the two important proteins in the brain, with greater frequency compared with SUMO‐2 or SUMO‐3.^28^ SUMO‐1 is predominantly abundant in the nuclear membrane, while SUMO‐2 and SUMO‐3 are typically found in the nuclear bodies and cytoplasm, respectively.^26^ SUMO‐1 is involved in the development and associated function of neurons; thus, alternations in neuronal SUMO‐1 modification are believed to be associated with impaired cognition, possibly being involved in the underlying mechanisms of mental retardation and a variety of NDDs, particularly AD and PD diseases.^46^ Protein transport into the nucleus may be affected by SUMO‐mediated changes, which could contribute to the development of diseases such as spincerebellar ataxia type 1 (SCA1), a dominantly inherited progressive NDD. The mutant ataxin‐1 in SCA1 accumulates and is modified by SUMO‐1 via different Lys residues, negatively regulating ataxin‐1 SUMOylation by enhancing the length of the polyglutamine (poly‐Q) tract (82Q). Accumulation of mutant ataxin‐1 [82Q] in nuclear inclusions is a hallmark of the disease, characterized by a functional nuclear localization signal (NLS). Mutations in the NLS lead to cytoplasmic localization and reduced pathology. The SUMO modification is dependent on the NLS and is influenced by phosphorylation. This transport defect has the potential to disrupt gene transcription, a common feature of poly‐Q disorders. Additionally, SUMOylation plays a critical role as a transcriptional regulator for several poly‐Q‐related proteins.^26^, ^49^

### 2.1 Effects of abnormal SUMOylation proteins in AD

AD is undeniably the most prevalent neurodegenerative disorder affecting the CNS. It primarily affects the elderly population and is characterized by chronic dementia. The disease is associated with synaptic dysfunction, a gradual decline in cognitive function, memory loss, neuropsychiatric symptoms, and impairments in language and skilled movements. One interesting aspect related to AD is the process of SUMOylation, which involves the attachment of SUMO proteins to various target proteins in neurons. Dysregulation of SUMOylation has been linked to AD, although the specific mechanisms underlying this association are not yet fully understood. Previous studies have indicated that changes in SUMOylation likely contribute to the development and progression of AD. Several proteins have been identified as relevant to AD pathology, and they are known to undergo SUMOylation. There are a number of proteins in the brain that have been found to undergo SUMOylation. Notably, Tau, amyloid precursor protein (APP), β‐secretase 1, and histone deacetylase 1 (HDAC1) are among the proteins associated with AD that have been reported to be involved in SUMO‐related processes. These proteins play vital roles in the regulation of synaptic physiology, mitochondrial dynamics, and inflammatory signaling.^50^ Histopathological analyses have revealed distinctive brain regions that are prone to the formation of neurofibrillary tangles (NFts) and SP, which are characteristic features of AD. NFts are aggregates of a hyperphosphorylated form of the microtubule‐associated protein Tau (MAPT), while SP contain aggregated fragments of the Aβ peptide. In recent research, the proteins Tau and APP have been found to be connected to the SUMOylation process. Studies have suggested that SUMOylation could play a role in the regulation of Tau and APP in AD. Abnormal SUMOylation of these proteins has been observed in the brains of individuals with AD, and it is thought to contribute to the pathological processes associated with the disease.^36^, ^50^ The APP is processed by the β‐ and γ‐secretase enzymes to produce the protein Aβ, which is involved in the physiology and plasticity of synaptic connections.^37^ In the pathophysiology of AD, the presence of Aβ peptides can trigger reactive responses in microglial cells and astrocytes. This, in turn, leads to the release of proinflammatory chemokines and cytokines, upregulation of GFAP, which is an intermediate filament protein, morphological changes in astrocytes, and the exacerbation of neurotoxicity. Chemokines and cytokines can activate various intracellular signaling pathways, such as protein kinase C, c‐Jun N‐terminal kinase (JNK), p38 mitogen‐activated protein kinase (p38/MAPK), PI3 kinase, extracellular signaling‐related kinase, and caspase‐1/3, all of which have been implicated in AD.^36^

#### 2.1.1 Abnormal SUMOylation of Tau protein in AD

Tau is an essential protein that plays a crucial role in microtubule binding and is highly abundant in neurons, particularly within the axons. The expression patterns of Tau exhibit distinct variations during different stages of development and in specific brain regions. In the adult human brain, six isoforms of Tau are expressed, ranging in length from 352 to 441 amino acids. This diversity in expression arises from the alternative splicing of three exons (2, 3, and 10) within the gene encoding the MAPT. The isoforms of Tau exhibit structural variations in terms of the quantity of N‐terminal inserts and C‐terminal repeat domains. Consequently, the N‐terminal inserts are expressed in different forms at varying levels, designated as 0, 1, and 2 N‐terminal inserts (0N, 1N, and 2N, respectively). Furthermore, Tau protein isoforms can be classified based on the number of microtubule‐binding repeats (MTBRs) present in their C‐terminal domain. Isoforms with four MTBRs are referred to as four‐repeat (4R) Tau, while those with three MTBRs are known as three‐repeat (3R) Tau. Consequently, any aberrant PTM within Tau domains, especially in the 3R or 4R binding repeats, results in the destabilization of microtubules.^51^, ^52^, ^53^ Tau protein molecular weights also vary, with approximate values ranging from 36.8 to 45.9 kDa.^54^ Therefore, Tau comprises distinct domains, including the N‐terminal (also known as the projection domain), the proline‐rich, microtubule‐binding repeat (MTBR), and the C‐terminal. Significantly, the proline‐rich domain also assumes a crucial function in mediating the binding of Tau to microtubules.^55^ The different isoforms of Tau play essential physiological roles in regulating microtubules and their functions, such as polymerization and stabilization, which involve several neuronal functions in the brain.^13^, ^56^ Additionally, Tau protein is known to possess certain characteristics, such as being heat‐resistant, highly soluble, and naturally unfolded.^28^ This abnormal hyperphosphorylation of Tau is associated with various NDDs collectively known as tauopathies.^57^ The Tau protein is susceptible to various PTMs, containing phosphorylation, methylation, acetylation, SUMOylation, and ubiquitination.^56^ While Tau serves various functions in neurons, the abnormal PTMs of Tau, especially hyperphosphorylation, cause its accumulation and subsequent neuronal death.^53^, ^58^ In AD, the presence of NFts composed of hyperphosphorylated Tau (p‐Tau) is accompanied by the presence of ubiquitin. Studies have demonstrated that ubiquitin levels are significantly elevated in the brains of individuals with AD.^59^ Moreover, Tau serves as a substrate for tumor necrosis factor receptor‐associated factor 6 (TRAF6), a known ubiquitin E3 ligase. This interaction can lead to the degradation of Tau through ubiquitin‐independent proteasomal degradation, highlighting a novel pathway for the regulation of Tau protein levels and potentially offering insights into the development of therapeutic interventions for tauopathies.^50^

Tau can be SUMOylated by SUMO‐1, which occurs more frequently than SUMO‐2/3 and preferentially attaches at Lys 340. SUMOylation is frequently reported within the fourth microtubule‐binding repeat (4R) region, where Tau interacts with microtubules.^60^ Studies suggest that Tau SUMOylation promotes p‐Tau at several sites linked to AD. However, this effect can be effectively counteracted by either site‐specific mutagenesis of Tau at Lys340Arg (the SUMOylation site) or by concurrently inhibiting SUMO‐1 activity.^61^ SUMOylation of Lys‐340 can lead to an upregulation of Tau phosphorylation, causing an increase in insoluble Tau production and a decrease in Tau ubiquitination and degradation. Consequently, the combined impact of different PTMs may collaborate to regulate the aggregation of Tau through their cooperative interactions. The role of SUMOylation in Tau aggregation remains a topic of ongoing debate, and it may play a part in AD‐like Tau accumulation. p‐Tau probably promotes its SUMOylation, and, conversely, Tau SUMOylation enhances Tau phosphorylation. This, in turn, can reduce the solubility and ubiquitination of Tau through poly‐SUMOylation, ultimately inhibiting the ubiquitination‐mediated degradation of Tau.^29^, ^61^ Hence, when the proteasomal degradation process is inhibited, Tau tends to become more ubiquitinated and less SUMOylated. This suggests that there may be competition between SUMO and ubiquitin for the regulation of Tau stability. As a result, the regulation of Tau modifications may have significant implications for the pathogenic effects of neurodegenerative disorders.^38^ The C‐terminal microtubule domains of Tau protein contain several Lys residues that can be ubiquitinated, which can prevent SUMOylation at that site in AD.^62^ Interestingly, there is evidence to suggest that amyloid pathology may be necessary for Tau SUMOylation. In vivo, research on neuritic plaques in APP‐transgenic mice has shown that SUMO‐1 and ubiquitin exhibit distinct immunoreactivity in phosphorylated Tau aggregates. Specifically, in APP‐transgenic mice, SUMO‐1 immunoreactivity colocalized with phosphorylated Tau aggregates in amyloid plaques.^62^ However, in mutant Tau transgenic mice, SUMO‐1 immunoreactivity was not detected in Tau aggregates.^28^, ^62^

#### 2.1.2 Abnormal SUMOylation of the APP in AD

APP is a type I transmembrane glycoprotein with a molecular weight ranging from 100 to 140 kDa.^63^ It belongs to a protein family found in mammals, which includes two similar proteins known as APP‐like protein 1 (APLP1) and APP‐like protein 2 (APLP2). APP695 is predominantly expressed in neurons, while APP751 and APP770 are expressed in most tissues. There are three isoforms of APP, namely APP695, APP751, and APP770, which differ in length and contain 695, 751, and 770 amino acids, respectively.^64^ The APP protein undergoes cleavage by various proteases, resulting in the generation of a wide range of peptides through two distinct and well‐defined processing pathways: the amyloidogenic and nonamyloidogenic pathways. One of these peptides, known as the Aβ peptide, is derived from the amyloidogenic pathway and is considered a significant characteristic of AD. In AD, the cleavage of APP and the subsequent production of Aβ aggregates are primarily mediated by two proteases: β‐secretase (also known as beta‐site APP cleaving enzyme 1 (*BACE1*)) and γ‐secretase.^65^ The amyloidogenic processing of APP involves the cleavage of APP by *BACE1*, resulting in the production of two fragments: a soluble N‐terminal fragment (APPsβ) and a membrane‐bound C‐terminal fragment (C99). Subsequently, C99 is cleaved by γ‐secretase, leading to the generation of Aβ peptides with varying lengths (ranging from 38 to 43 residues) and a cytoplasmic polypeptide called the APP intracellular domain (AICD). AICD functions as a transcriptional factor within the nucleus, while the Aβ peptides are secreted into the extracellular space.^66^, ^67^ Therefore, AICD plays a crucial role in their functionality. The short cytoplasmic domain of AICD contains multiple functional and binding motifs, which collectively influence the trafficking and metabolism of APP.^68^ The APP protein is proposed to have multifaceted physiological roles, encompassing neural migration, cell adhesion, the facilitation of synaptogenesis,^69^ neurogenesis, neuronal differentiation, neurite outgrowth, axonal outgrowth, and the modulation of synaptic neurotransmission.^70^, ^71^ Nevertheless, the exact role of APP under normal conditions remains incompletely understood. In humans, APP has been associated with various pathophysiological processes related to neurodegenerative disorders, such as PD and AD.^72^ Likewise, a reduction or elimination of APP has been observed to correlate with neuronal loss and reduced synaptic activity in both in vitro and in vivo settings. Additionally, the reduction of APP in cultured neurons is associated with diminished neurite outgrowth.^73^ Although APP has been recognized for its potential neuroprotective functions, it has been extensively investigated in the context of AD, primarily because of its role in the generation of Aβ peptides.^65^ The accumulation of Aβ peptides is one of the key features of AD. Specifically, Aβ42 and Aβ43 are toxic due to their strong propensity for aggregation. Moreover, mutations in the APP and presenilin genes often lead to an elevated ratio of Aβ42 to Aβ40, which is a common characteristic observed in early‐onset familial AD (FAD) cases. Abnormal processing of APP by β‐secretase and γ‐secretase can disrupt the equilibrium between the production and clearance of Aβ, leading to the formation of toxic oligomers, fibrils, and SP, especially in the context of AD.^66^ Among the different forms of Aβ, Aβ1–42 has a propensity to rapidly and dynamically oligomerize, eventually forming insoluble fibrils that aggregate into plaques.^74^

APP undergoes various PTMs that can contribute to amyloidogenesis and disease pathogenesis in the context of AD. Some of the known PTMs on APP include palmitoylation, ubiquitination, N‐glycosylation, and phosphorylation.^75^ There is some evidence to suggest that other PTMs may exert protective effects against AD, such as O‐GlcNAcylation, ubiquitylation, and SUMOylation.^76^, ^77^ Studies have highlighted the significant involvement of SUMOylation in the regulation of APP. It has been identified as a potential substrate for this PTM, with particular emphasis on two Lys residues located at positions 587 and 595. These Lys residues are considered key targets for SUMOylation, suggesting that this modification may play a crucial role in modulating the functions and processing of APP. Notably, SUMO‐1 and SUMO‐2 can directly modify these Lys residues, which are located adjacent to the β‐secretase cleavage site. Studies have demonstrated that SUMOylation at these sites can attenuate the production of Aβ. Eliminating the two SUMOylations at Lys residues 587 and 595 has been shown to stimulate the high generation of Aβ aggregates, suggesting that both SUMO‐1 and SUMO‐2 may interfere with the β‐secretase cleavage site and inhibit Aβ aggregate formation. This result raises the possibility that the mutation of Lys‐595 to asparagine in the Swedish (KM‐to‐NL) APP mutant could prevent SUMOylation at this residue and contribute to the production of Aβ in the Swedish APP mutant.^37^, ^78^ The modifications of SUMO‐1 and SUMO‐2 have demonstrated a significant capability of reducing the levels of Aβ aggregates. However, despite their effectiveness, the exact mechanism of this regulation remains elusive. The attachment of the SUMO protein at Lys 587 and 595 may sterically hinder the binding of β‐secretase to APP.^79^ In mouse models of AD, an upregulation of SUMO‐1 expression has been observed in association with high levels of *BACE1*. This increase in *BACE1* expression is enhanced in both sporadic and FAD, as well as in ischemic stroke, which is a significant risk factor for AD. This research demonstrates that SUMO‐1 interacts with *BACE1*, leading to *BACE1* accumulation and provoking the generation of Aβ peptides. Conversely, the depletion of SUMO‐1 in this study resulted in the elimination of BACE1 protein and a reduction in Aβ levels. Furthermore, the available empirical evidence strongly supports the notion that the SUMOylation of BACE1 at the Lys 501 residue is critical for controlling its enzymatic activity and stability, which in turn affects Aβ production and cognitive function in AD mouse models.^80^ These findings highlight the critical role of Lys 501 SUMOylation on *BACE1* in regulating its stability and Aβ‐generating activity. Notably, overexpression of the non‐SUMOylated *BACE1* mutant did not affect memory decline in wild‐type mice and did not hasten the formation of SP, suggesting that SUMOylation of *BACE1* plays a crucial role in AD pathogenesis.^67^, ^80^ In addition to *BACE1*’s involvement in the amyloidogenic pathway, it has been implicated in a variety of physiological processes and key pathophysiological mechanisms of AD. *BACE1* is widely expressed in the brain, with high levels in specific neuronal cell types, oligodendrocytes, and astrocytes. *BACE1* also localizes to various subcellular compartments, such as the plasma membrane and endosomes, indicating its involvement in multiple physiological and pathological processes within the brain.^67^ Moreover, the overexpression of wild‐type *BACE1* facilitates the formation of SP and aggravates cognitive deficits in AD mouse models.

Interestingly, studies have shown that the non‐SUMOylated mutant of BACE1 is susceptible to lysosomal degradation, leading to a decrease in Aβ formation and a reduction in cognitive dysfunction in mouse models of AD.^80^ In contrast to other studies, research on fangchinoline, an alkaloid derived from the traditional Chinese medicine Stephania tetrandra S. Moore, has shown promising therapeutic effects in mouse models of AD. Specifically, fangchinoline has been found to promote the autophagy–lysosomal degradation of BACE1 without affecting its sumoylation. This mechanism contributes to the reduction of amyloidogenic processing of APP and the improvement of cognitive impairment.^81^ Moreover, it has been documented that BACE1 SUMOylation promotes the amyloidogenic processing of APP, leading to the production of Aβ aggregates. In the context of AD mouse models, BACE1 SUMOylation has been shown to suppress its phosphorylation at the S498 site. This observation aligns with studies indicating reduced phosphorylation of BACE1 at S498 in AD brain tissues. Furthermore, BACE1 SUMOylation suppresses its ubiquitination in mouse models of AD, similar to the findings regarding Tau SUMOylation and its impact on ubiquitination, which can contribute to Tau aggregation. Additionally, it has been observed that BACE1 phosphorylation inhibits its SUMOylation, promoting BACE1 degradation in vitro. Consequently, BACE1 SUMOylation reciprocally regulates its phosphorylation and ubiquitination processes.^82^

It should be noted that SUMO‐3 overexpression or facilitating SUMOylation can also modulate APP processing by reducing the amyloidogenic pathway and simultaneously enhancing the nonamyloidogenic pathway. SUMO‐3 is detected and restricted in neuronal soma in brains from AD or Down's syndrome. The overexpression of SUMO‐3 can be associated with the reduction of Aβ levels, although it does not have a direct interaction with APP protein levels.^83^ In this study, both APP and *BACE1* were increased along with the overexpression of SUMO‐3. Hence, in contrast to mono‐SUMOylation, poly‐SUMOylation is responsible for reducing Aβ production.^83^ Nevertheless, overexpression of SUMO3 leads to increased secretion of both Aβ40 and Aβ42 peptides and the upregulation of BACE1 expression. Conversely, it seems that endogenous sumoylation seems to play only an indirect role in modulating the amyloid processing pathway. Notably, overexpression of monomers of SUMO3 noncovalently leads to Aβ peptide production.^84^ Consequently, the precise action of SUMO‐3 in APP processing needs more investigation. Furthermore, SUMOylation of AICD is a protective mechanism in AD by facilitating the degradation of Aβ.^85^ According to all these contradictory data, further investigation is warranted to elucidate the precise mechanisms by which SUMOylation influences APP‐related pathways and its implications for various cellular processes and AD progression.

### 2.2 Effects of abnormal SUMOylation of proteins in PD

PD is a common movement disorder characterized by the degeneration of dopamine‐producing cells in the brain, resulting in a range of motor and nonmotor symptoms. The condition was initially referred to as “shaking palsy” and was first described by James Parkinson, a British physician, in 1817.^86^ PD is the second most prevalent NDD, following AD, in terms of its occurrence.^87^ PD affects more than six million people globally and is the second most common neurological disorder.^88^ One of the main risk factors for PD is age; around 2% of adults over 65 and 4% of adults over 85 have the disease.^89^ In recent years, several proteins, including α‐syn, DJ‐1, and Parkin, have been identified as being associated with PD pathology. These proteins undergo abnormal PTMs, which play crucial roles in altering their physiological functions relevant to the disease. PD is characterized by specific neuropathological features, including the degeneration of neurons in the substantia nigra (SN), leading to a decline in dopaminergic neurons within the nigrostriatal pathway. Among the key proteins implicated in PD, α‐syn holds significant importance in disease pathology. Alterations in the folding of α‐syn critically affect its physiological function. These alterations result in the aggregation of α‐syn into intraneuronal inclusions known as Lewy bodies (LBs). LBs contain aggregated α‐syn along with other components, such as neurofilament proteins. The aggregation of α‐syn is a major pathological hallmark that contributes to the development of PD.^90^ It was observed that both PD and PD with dementia (PDD) consistently displayed elevated levels of insoluble α‐Syn in dopaminergic neurons and the nigrostriatal region. Notably, PDD exhibited higher levels compared with PD.^91^ The accumulation of intracellular proteins, particularly α‐syn, is a contributing factor to PD. This accumulation is facilitated by the process of SUMOylation, which exacerbates the accumulation of α‐syn by counteracting its clearance. Several studies have investigated the role of SUMOylation in the development of PD and have identified a subset of proteins that undergo this PTM. These proteins are believed to play a role in the pathological processes underlying the disease. Among the proteins that undergo SUMOylation, α‐syn, DJ‐1, Parkin, dynamin‐related protein 1 (Drp1), and peroxisome proliferation ‐ activated receptor‐ gama‐ coactivator (PGC‐1α) have been found to exhibit a high frequency of occurrence and are involved in the pathogenic processes of PD.^38^, ^92^

#### 2.2.1 Abnormal SUMOylation of α‐syn protein in PD

The α‐syn protein is encoded by the α‐syn gene, also known as sodium voltage‐gated channel alpha subunit 1 (*SNCA1*). It consists of 140 residues and has a molecular weight of approximately 14 kDa.^28^, ^93^ α‐Syn is part of a protein family called synuclein, which also includes β‐syn and γ‐syn.^93^ These three members of the syn family are widely expressed in neurons and are primarily located at presynaptic terminals. Additionally, α‐syn constitutes approximately 1% of the total cytosolic protein.^93^, ^94^ A subgroup of patients with early‐onset PD had a mutation in the α‐syn gene, SNCA, which led to the association between α‐syn and PD. A53T/E, A30P, E46K, H50Q, and G51D are the six SNCA gene variants that have been connected to PD; SNCA duplication and triplication have also been identified to produce early‐onset familial PD.^95^ α‐Syn is abundantly expressed in various types of neurons in both the peripheral and CNS, including during synapse development. α‐Syn plays a multifaceted role in neuronal function. For instance, α‐syn is a prominent neuronal protein primarily localized within presynaptic terminals. Its central function lies in the regulation of synaptic vesicle (SV) clustering and trafficking, notably without being found inside the vesicles themselves. Moreover, it can inhibit neurotransmitter release when it is overexpressed and interacts with the integral membrane proteins of SVs. Despite its involvement in various cellular processes, the precise physiological function of α‐syn remains unclear.^96^, ^97^ Moreover, α‐syn is typically found in neurons as a membrane‐bound α‐helical state and as an inherently disordered monomer.^98^ When dysregulated, α‐syn may accumulate together to create cross‐β amyloid fibrils through less structured oligomeric intermediates, which seem to be especially harmful to neurons.^99^

α‐Syn is composed of three main domains, each serving a specific function. The first domain is located in the N‐terminal region and is referred to as the amphipathic region. It consists of 60 residues and plays a critical role in membrane binding due to its amphipathic nature. α‐Syn is composed of three main domains, each serving a distinct function. The second domain of α‐syn is called the nonamyloid component (NAC) region, comprising 34 residues. The NAC region is involved in the aggregation and fibrillation of α‐syn, contributing to the formation of pathological aggregates seen in PD. Finally, the C‐terminal region of α‐syn consists of 44 residues. This region is involved in various interactions with other proteins and cellular components, and it also plays a role in regulating α‐syn's physiological function and its aggregation propensity.^100^ The α‐syn protein is classified as an intrinsically disordered protein due to its heat resistance, high solubility, and natural unfolded state.^28^, ^101^ However, structural alterations in α‐syn have been observed in various studies.^93^, ^102^ While the molecular pathways underlying the involvement of α‐syn in diseases like PD are still not fully understood, it is evident that PD is characterized by the aggregation and accumulation of α‐syn.^103^ During this process, α‐syn aggregation leads to the formation of off‐pathway, nonfibrillar, and soluble oligomers. These transient prefibrillar intermediate species, known as oligomers, eventually transform into insoluble fibrillar aggregates that exhibit a specific cross β‐sheet conformation. The abnormal aggregation of α‐syn oligomers plays a crucial role in the development of neurodegenerative conditions, collectively referred to as α‐synucleinopathies. These conditions encompass PD, dementia with LB, and multiple system atrophy (MSA).^104^ In α‐synucleinopathies, a prominent pathological feature is the presence of LBs, which are intraneuronal inclusions. These bodies consist of aggregated α‐syn that is immunoreactive and include protein inhibitors that spread to neighboring neurons.^105^ Furthermore, α‐syn aggregates can transmit their pathological conformation to other α‐syn molecules in nearby cells, similar to the prion‐like spread, resulting in the progression of the disease. In addition to the neurotoxic effects of α‐syn oligomers, α‐syn fibrils also play a crucial role in the propagation and spread of α‐synucleinopathies.^104^, ^106^ The precise mechanisms underlying the emergence of Lewy pathology in transplanted dopamine neurons are not yet fully understood. However, empirical research suggests that the activation of microglia and the subsequent release of cytokines during immune activation may contribute to the misfolding of α‐syn and facilitate its spread.^107^ The misfolding and aggregation of α‐syn involve a complex, multi‐step process that includes various sequential conformational changes. Therefore, to gain a deeper understanding of PD, it is essential to investigate the initial stages of α‐syn aggregation, particularly the process of oligomerization.^93^, ^104^

Multiple PTMs have been reported for α‐syn, including glycation, truncation, acetylation, nitration, phosphorylation, ubiquitination, and SUMOylation.^103^ Abnormal modifications in these PTMs can impact the structure and function of α‐syn, leading to protein dysfunction and potentially contributing to the development of PD.^28^, ^103^ In LBs, various notable modifications have been identified in α‐syn. These modifications include phosphorylation at Ser‐129, ubiquitination at specific Lys residues (12, 21, and 23), and specific truncations at Asp‐115, Asp‐119, Asn‐122, Tyr‐133, and Asp‐135. These distinct PTMs contribute to the unique characteristics of the aggregated α‐syn protein. Interestingly, this pattern of modifications has been consistently observed not only in sporadic PD but also in familial PD and MSA. This suggests that the preferential accumulation of normally produced Ser‐129 phosphorylated α‐syn may be a primary factor in the formation of LBs, a pathological hallmark of PD and related disorders.^108^ Following the process of inclusion formation, ubiquitination of α‐syn may occur subsequently, but it is not a prerequisite for α‐syn fibrillization and inclusion formation.^109^ A study has shown that the level of ubiquitination in the assembled, filamentous form of α‐syn is reduced compared with its soluble form. The major sites of ubiquitination are located at Lys‐6, 10, and 12 in the amino‐terminal region of the α‐syn molecule. These findings suggest that ubiquitination occurs after the formation of α‐syn filaments, and the same may hold for α‐synucleinopathies observed in the brains of affected individuals.^110^ The process of α‐syn SUMOylation serves as a mechanism to counterbalance the effects of ubiquitination, thereby mitigating its degradation through the proteasome. Several studies have suggested that α‐syn SUMOylation is associated with increased steady‐state levels and aggregation, as well as potentially enhancing its exosomal release and nuclear translocation.^103^ Consequently, SUMOylation impedes the degradation of α‐syn and contributes to its accumulation by inhibiting ubiquitination.^111^ α‐Syn possesses two Lys sites, Lys‐96 and Lys‐102, which are more readily targeted by SUMO‐1 than SUMO‐2/3. Several E3 ubiquitin ligases, including protein inhibitors of activated STAT 2 (PIAS2), hPc2, and tripartite motif containing 28 (TRIM28), have been identified to SUMOylate α‐syn, resulting in either mono‐SUMOylation or multi‐monoSUMOylation.^28^, ^103^ Evidence suggests that SUMOylation at the Lys102 residue inhibits the aggregation of α‐syn more effectively than at Lys‐96, and modification via SUMO‐1 is a stronger inhibitor than SUMO‐3.^112^

The elevated levels of SUMOylated α‐syn and SUMOylation machinery components in PD brains indicate a crucial role of SUMOylation in the aggregation of α‐syn and formation of LBs. Consequently, the inhibition of SUMOylation could be a promising strategy for preventing the accumulation, aggregation, and propagation of α‐syn in PD.^111^ Studies have identified PIAS2 as a SUMO E3 ligase responsible for increasing SUMOylation in the α‐syn. Furthermore, ubiquitination of α‐syn typically directs it to the proteasome for degradation. This process is reduced by both the E3 ubiquitin‐ligase seven in absentia homolog (SIAH) and neural precursor cells that express developmentally downregulated 4 (Nedd4) E3 ubiquitin ligases. However, PIAS2‐mediated SUMOylation of α‐syn reduces E3 ubiquitin ligases. As a result, α‐syn accumulates and aggregates within inclusions. These findings suggest that maintaining a balance between α‐syn SUMOylation and ubiquitination is critical for its proper clearance and degradation. Dysregulation of this balance may contribute to the pathogenesis of PD and related disorders.^111^ Additionally, the ubiquitination of α‐syn by SIAH‐1 does not result in its degradation via the proteasome; rather, it promotes the aggregation of α‐syn and enhances its toxicity. This study suggests that SIAH‐1‐mediated α‐syn ubiquitination could significantly influence the development of LBs and the pathogenesis of related conditions.^113^ Furthermore, the SUMOylation of α‐syn by PIAS2 promotes aggregation both directly and indirectly by impeding degradation and facilitating accumulation through the inhibition of ubiquitin‐dependent pathways.^111^ In particular, increased expression of PIAS2 has been detected in the tissues of individuals with PD, providing further evidence of the SUMOylation role in the disease.^103^, ^114^ Therefore, SUMOylation inhibitors may help reduce α‐syn levels and mitigate PD‐related aggregation. As a result, there is a decrease in the accumulation, aggregation, and spreading of intracellular α‐syn in PD.^111^

#### 2.2.2 Abnormal SUMOylation of Parkin protein in PD

**FIGURE 3:**
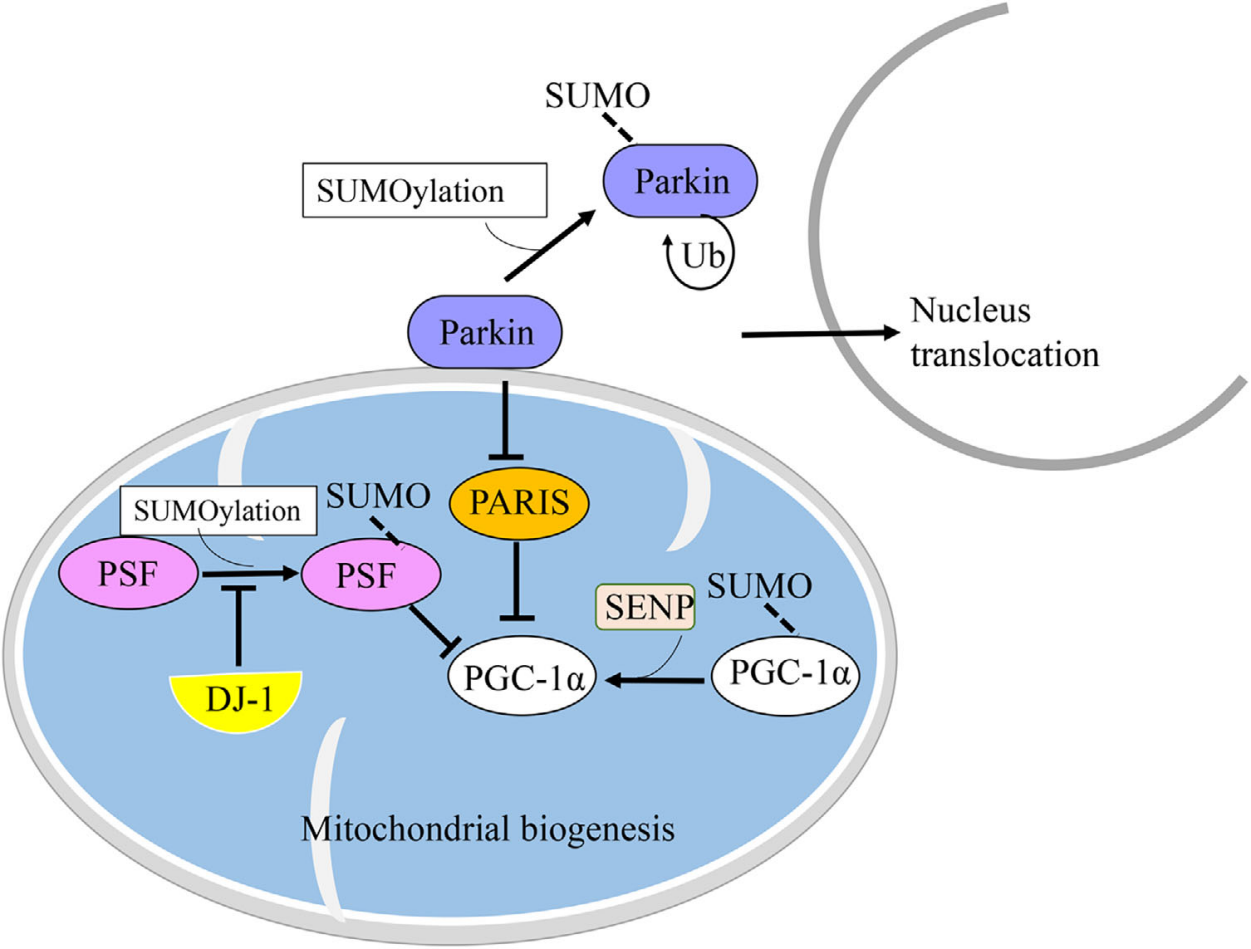
The SUMOylation of parkin triggers its own self‐ubiquitination and prompts it to move into the cell's nucleus. This process consequently reduces the pool of parkin accessible for mitochondrial recruitment. Parkin dysfunction disrupts normal mitochondrial biogenesis due to the accumulation of a specific protein called PARIS, which negatively regulates PGC‐1α expression. While SENP enhances the transcriptional activity of PGC‐1α by removing SUMO tags, the presence of SUMO and PSF inhibits PGC‐1α’s ability to activate genes. DJ‐1, associated with an early‐onset recessive condition, hampers the SUMOylation of PSF, leading to a reduction in the creation of new mitochondria. SENP, sentrin‐specific proteases; PSF, pyrimidine tract‐binding protein‐associated splicing factor.

Parkin (also known as PARK2) is a protein of approximately 52 kDa, encoded by the PARK2 gene.^115^, ^116^ It is primarily expressed in various tissues, with a notable presence in the brain and muscles.^117^ Parkin serves as a unique multifunctional ubiquitin ligase, functioning as an E3 ubiquitin ligase. Its role involves attaching ubiquitin molecules to specific protein substrates. Thereby, it can play a crucial role in the process of ubiquitination and direct the proteins for degradation through the UPS.^118^, ^119^, ^120^ Remarkably, Parkin has diverse functions, especially within neurons, which are believed to be highly beneficial and serve a protective purpose.^120^ Parkin is known to target a diverse array of over 20 different substrates, some of which play a crucial role in neuronal physiology and pathology. These substrates include O‐glycosylated α‐syn, synphilin‐1, CDCrel‐1 (a member of the septin family), Parkin‐associated endothelin receptor‐like receptor, and dopamine transporter. As an E3 ubiquitin ligase, Parkin functions to prevent protein accumulation.^119^, ^121^ However, the loss of Parkin's E3 ligase function can lead to substrate accumulation, contributing to neurotoxicity and the development of pathological conditions in NDDs.^119^, ^121^ Therefore, Parkin plays an essential role in neuronal protection. It acts as a neuroprotective factor by promoting the removal of defective mitochondria, preventing protein accumulation, and shielding cells from excitotoxicity and unfolded protein stress.^118^, ^121^ Furthermore, Parkin's involvement in various aspects of mitochondrial functioning, including mitochondrial fusion/fission, mitochondrial biogenesis, mitochondrial transport, mitophagy, ER–mitochondrial interactions, cell signaling, and apoptosis, is tightly interconnected. The dysfunction of Parkin has been implicated in neurodegenerative disorders.^118^ However, recent research has also characterized Parkin as a tumor suppressor.^122^ Mutations in the Parkin gene cause autosomal recessive PD. It may also be involved in sporadic PD. According to a few studies, patients with Parkin mutations indicate dopaminergic neuronal loss in the SN and noradrenergic neuronal loss in the locus coeruleus, with accompanying gliosis.^115^ Moreover, the pathogenicity of PD appears to be associated with dysfunctional mitochondria, oxidative stress, and protein misfolding and aggregation.^115^, ^121^ It is noteworthy that activation and/or suppression of Parkin can be regulated through various PTMs, including phosphorylation, ubiquitination, nitrosylation, sulfhydration, sulfonation, SUMOylation, and neddylation.^123^ Studies suggest that Parkin plays a critical role in PD, as it has been found in LBs, which are the pathological hallmark of the disease. Hence, Parkin dysfunction has been identified as a crucial factor in the pathogenesis of PD.^103,109^ Furthermore, reduced proteasomal activity leads to the accumulation of Parkin and a subsequent decrease in its ligase activity. This ultimately results in the formation of noncytotoxic inclusions. These findings provide additional evidence supporting the involvement of Parkin dysfunction in the development of PD.^124^, ^125^ For instance, nitrosylation can inhibit Parkin's activity as an E3 ubiquitin ligase.^123^ Additionally, dephosphorylation of Parkin can enhance its activity in cells under ER stress, allowing it to combat unfolded proteins.^126^ Phosphorylation of Parkin has been reported to increase its activity, but the absence of phosphorylation or the presence of an aberrant constitutive form that leads to overactive Parkin can be detrimental.^127^ In addition to these PTMs and their effects, SUMOylation has recently gained attention in drug discovery due to its regulatory role in various processes.^37^, ^76^ Parkin is a substrate for SUMOylation.^128^ It has been observed that SUMO‐1 binds covalently to the Parkin protein in both nonneuronal and neuronal cells.^128^ The SUMOylation of Parkin through SUMO‐1 reduces its availability for mitochondrial recruitment by increasing its self‐ubiquitination (Ub) and facilitating its translocation from the cytoplasm to the nucleus.^128^ Consequently, the interaction of SUMO‐1 with Parkin can alter its intracellular localization and its E3 ubiquitin ligase activity.^128^ Moreover, impaired Parkin function can suppress mitochondrial biogenesis by leading to an accumulation of Parkin interacting substrate (PARIS), subsequently reducing the levels of peroxisomal proliferator‐activated receptor‐gamma coactivator 1‐alpha (PGC‐1α).^128^, ^129^ These findings suggest that SUMOylation, specifically through SUMO‐1, can influence the activity and localization of Parkin.^38^ Given Parkin's involvement in various mitochondrial activities and the clearance of substrate accumulation, SUMOylation of Parkin may have implications for PD (Figure 3).^118^

#### 2.2.3 Abnormal SUMOylation of DJ‐1 protein in PD

DJ‐1, also known as DJ‐1/PfpI, ThiJ/PfpI, or DJ‐1/ThiJ/PfpI, is a small protein that belongs to a superfamily. It is expressed in various human tissues, including the brain, skeletal muscle, liver, kidney, and more.^130^, ^131^, ^132^ In the human brain, DJ‐1 is predominantly expressed within astrocytes and exhibits sensitivity to oxidative stress situations.^133^ DJ‐1 exists in 10 isoforms in the human brain, consisting of six monomeric forms and four dimeric forms.^134^ DJ‐1 is present in both glial and neuronal cells, localized in the cytoplasm and nucleus.^135^ The DJ‐1 protein is encoded by the Parkinsonism‐associated deglycase gene (*PARK7*) and consists of 198 residues, weighing approximately 20 kDa.^130^, ^136^ Mutations in *PARK7* have been implicated in familial PD.^80^ Initially identified in 1997, the *PARK7* gene was recognized as a mitogen‐dependent oncogene associated with a Ras‐related signal transduction pathway. DJ‐1 is a highly conserved intracellular protein.^137^

The DJ‐1 protein serves multiple roles, including functioning as a molecular chaperone, enzyme, and protease. It also regulates mitochondrial homeostasis, transcription pathways, antioxidative stress reactions, and oxidative stress‐induced apoptosis. Additionally, DJ‐1 acts as a stress sensor and is upregulated in response to various stresses, particularly oxidative stress.^138^ In neurons, DJ‐1 not only acts as a sensor and protects neurons against reactive oxygen species (ROS) and oxidative phosphorylation, but it also inhibits the aggregation of α‐syn through chaperone‐mediated autophagy (CAM). Studies have demonstrated that the suppression of DJ‐1 leads to increased levels of aggregated α‐syn in experimental models of PD. Conversely, overexpressing DJ‐1 reduces the levels of α‐syn. These findings highlight the significance of DJ‐1 in maintaining the balance of α‐syn and suggest that it could be a potential therapeutic target for PD treatment.^136^, ^138^ There is substantial evidence linking DJ‐1 to various types of cancer through the activation of signaling pathways, particularly the Wnt/β‐catenin signaling pathway, which promotes proliferation, invasion, and metastasis.^136^, ^139^ However, the precise contribution of DJ‐1 to cell survival in cancer remains unclear.^139^ Notably, research suggests that the DJ‐1 protein could serve as a potential biomarker for the diagnosis and prognosis of certain cancers, including acute leukemia. Its overexpression in cancer cells and its presence in blood, secretory fluids, ascites, and pleural effusion make it a promising candidate for diagnostic purposes.^136^, ^140^ Additionally, DJ‐1 may also be an attractive therapeutic target in cancer treatment. Furthermore, DJ‐1 has been implicated in the onset and pathogenesis of not only sporadic PD but also familial PD, where it can become inactive due to excessive oxidation.^138^ DJ‐1 has been associated with other NDDs, such as HD, amyotrophic lateral sclerosis (ALS), AD, ischemia–reperfusion injury, and autosomal recessive disorders.^141^, ^142^

The main cause of PD is the degeneration of dopaminergic neurons in the SN, leading to a reduction in the levels of a neurotransmitter known as dopamine in the striatum.^143^, ^144^ This deficiency of dopamine is primarily responsible for the manifestation of motor deficits commonly observed in PD. However, it is important to note that dopamine may also play a role in the cognitive impairments observed in some individuals with PD. DJ‐1 protein plays a protective role in preventing neurodegeneration in dopaminergic neurons associated with PD, employing various mechanisms.^143^ It regulates adenosine triphosphate (ATP) synthase protein components by interacting with the ATP synthase β subunit, thereby promoting dopaminergic cell metabolism and growth.^145^ DJ‐1 performs activities like chaperone that can influence α‐syn aggregation. Therefore, DJ‐1 can suppress α‐syn aggregation by taking part in the beginning step of the aggregation process. In vitro experiments suggest that DJ‐1 can directly interact with α‐syn monomers and oligomers. Overexpression of DJ‐1 reduces α‐syn dimerization and significantly lowers α‐syn levels, resulting in increased aggregation. However, mutations in DJ‐1 in familial PD disrupt this process.^146^ Loss of DJ‐1 expression and transcriptional dysfunction are directly associated with impaired dopamine synthesis, which contributes to the development of PD. Patients with DJ‐1 mutations exhibit reduced dopamine uptake, although it remains unclear whether the loss of DJ‐1 function affects the survival of dopaminergic neurons. The primary causes of neurodegeneration in PD are believed to be associated with oxidative stress and mitochondrial malfunction.^138^ Reduced or lost DJ‐1 function triggers the onset of oxidative stress‐related diseases, particularly in PD.^138^

DJ‐1 is used as a substrate for SUMOylation, with Lys‐130 being the main Lys residue for SUMOylation. This residue becomes conjugated to SUMO‐1 through the two E3 SUMO‐1 ligases, namely PIASxa or PIASy.^147^ Mutations in Lys‐130 impair various functions of DJ‐1, including its role in anti‐UV‐induced apoptosis, Ras‐dependent transformation, and cell growth promotion. Exposure of DJ‐1 to UV radiation leads to an increase in its SUMOylation, causing it to undergo a shift toward a more acidic state.^147^ SUMOylation plays a critical role in repressing the transcriptional activity of p53 by DJ‐1. In this regard, the non‐SUMOylated form of DJ‐1, known as DJ‐1 (K130R), undergoes translocation from the nucleus to the cytoplasm, resulting in the loss of its protective function against ultraviolet (UV)‐induced cell death by failing to repress p53 transcriptional activity.^148^ The M26I mutation in DJ‐1 disrupts its interaction with SUMO‐1, leading to erroneous SUMOylation of DJ‐1. As a consequence, it contributes to its insolubility, partial localization in mitochondria, and degradation by the proteasome system.^149^ Despite DJ‐1's homodimeric nature, a mutation on the Leu‐166 residue reduces its stability and causes it to exist as a monomer, thereby interfering with homodimerization.^150^, ^151^ Consequently, the Lys 166 mutation is considered a fundamental cause of early‐onset PD, as it results in the loss of normal DJ‐1 activity and promotes its degradation through the UPS.^150^, ^151^ Improper SUMOylation of the Lys‐166 mutant DJ‐1 renders it insoluble, which may contribute to the onset of PD.^147^

#### 2.2.4 Abnormal SUMOylation of dynamin‐related protein 1 in PD

Dynamin‐related protein 1 (Drp1) is a multifunctional protein encoded by the DNM1L gene, consisting of 736 amino acids.^152^ Drp1 consists of four distinct domains: the middle domain, the variable domain (VR), and the C‐terminal GTPase effector domain.^153^ It is primarily located in the cytoplasm, and it has also been observed in various organelles such as mitochondria, Golgi, and peroxisomes.^154^ Drp1 plays a crucial role in maintaining mitochondrial dynamics, specifically in processes such as fission, distribution, and potentially peroxisomal fragmentation. Notably, impaired mitochondrial dynamics can occur early in the development of NDDs.^155^ This suggests that the proper functioning of Drp1 and mitochondrial dynamics is vital for preserving cellular health and preventing the onset of disease. Generally, a variety of diseases, including neurodegenerative disorders, cardiovascular diseases, and different types of cancer, disrupt mitochondrial dynamics, which play a critical role in sustaining normal cellular function. Therefore, gaining a deeper understanding of the mechanisms governing mitochondrial dynamics and their regulation could pave the way for the development of novel therapies targeting these diseases.^156^, ^157^

Neurons have a high demand for energy production, and their proper functioning heavily depends on the activity of mitochondria. As a result, mitochondria are distributed throughout the cytosol to efficiently generate ATP through oxidative metabolism. Any abnormalities in mitochondrial dynamics, particularly impairments in Drp1‐mediated mitochondrial fission, can have a profound impact on various neurodegenerative disorders, such as AD, PD, and HD.^158^ In mitochondrial dynamics, Drp1 plays a crucial role as a key mediator in the fission processes, and it has a dynamic relationship with mitochondrial fusion.^154^ Fission and fusion processes not only regulate the morphology and size of mitochondria but also play important roles in mitophagy, organelle transport, and cell death pathways. These processes are involved in maintaining the overall health and functionality of the organelles as well as controlling the shape and size of mitochondria within the cell.^158^ During fission, two mitochondria are generated. Several outer mitochondrial membrane proteins, including fission‐1, mitochondrial fission factor, Mid49, and Mid51, act as receptors for Drp1 and recruit it to the site of mitochondrial fission.^154^ Therefore, the loss of Drp1 disrupts mitochondrial fission, leading to the formation of mitochondrial aggregates, which can impair neuronal development.^159^ Additionally, Drp1 is found within the ER lumen, and ER‐associated Drp1 promotes the formation of ER tubules.^159^ Given the crucial role of Drp1 in neuronal activities, investigating the PTMs of Drp1 that affect its function and morphology could be a valuable target in both normal conditions and NDDs.^155^, ^160^ Drp1 activity is tightly regulated through various PTMs, such as phosphorylation, SUMOylation, palmitoylation, ubiquitination, S‐nitrosylation, and O‐GlcNAcylation, as well as its interaction with other mitochondrial proteins.^161^ It appears that the Drp1 protein is the only mitochondrial target of SUMOylation.^162^ Research indicates that when Drp1 is located at the site of fission, SUMO is found to be localized there. This suggests that SUMOylation may affect the translocation of Drp1 from the cytosol to the mitochondria, which in turn induces mitochondrial fission.^37^ SUMOylation and deSUMOylation play a pivotal role in regulating mitochondrial dynamics by affecting Drp1 activity.^159^ Therefore, SUMOylation of Drp1 occurs through the interaction with the SUMO‐conjugating enzyme Ubc9.^155^ Drp1 can be modified by all three SUMO isoforms: SUMO‐1, SUMO‐2, and SUMO‐3. SUMOylation happens at two pairs of Lyss or more, particularly Lys532, 535, 558, and 568, which are located on the highly VR domain.^152^, ^153^ SUMOylation enhances the stability of Drp1, while deSUMOylation reduces its stability.^159^ Hence, SUMO‐1‐mediated SUMOylation of Drp1 promotes the localization of Drp1 on mitochondria and its attachment to mitochondria, the release of cytochrome *C*, and mitochondrial fragmentation. Conversely, SUMO‐2/3‐mediated SUMOylation of Drp1 decreases its localization and relocates it to the cytoplasm, leading to a reduction in mitochondrial fission.^159^ It has been reported that SUMO‐1 promotes the localization of Drp1 on mitochondria, while SUMO‐2 and SUMO‐3 decrease its localization. Furthermore, the fission process plays a role in apoptosis, and Drp1 is required for this condition. While it is known that Drp1 can undergo SUMOylation at multiple Lys residues on its VR domain, the precise consequences of the modification remain incompletely understood, including its impact on the clearance of damaged mitochondria through mitophagy. Further research is needed to fully elucidate the functional consequences of SUMOylation on Drp1 and its role in mitochondrial biology.^37^, ^163^

The inhibition of mitochondrial fission leads to the accumulation of cytochrome *C* within the mitochondria, thereby hindering the progression of apoptosis.^37^ Interestingly, research indicates that the upregulation of SUMO‐1 is associated with an increase in mitochondrial fission, which could potentially contribute to apoptosis.^155^, ^164^ It has been suggested that SUMO‐1 plays a protective role by preventing Drp1 degradation, stabilizing the Drp1 protein, and promoting its binding to mitochondria. This could explain why the upregulation of SUMO‐1 leads to mitochondrial fragmentation and apoptosis.^164^ Studies have shown that under oxidative stress conditions like myocardial ischemia/reperfusion (MI/R), Drp1 undergoes SUMOylation, resulting in its relocation from the cytosol to the mitochondria. This, in turn, triggers abnormal mitochondrial fission, leading to impaired morphology and function. Similarly, in the context of MI/R, the upregulation of SENP3 (a SUMO protease) without altering the overall expression of Drp1 is associated with increased mitochondrial Drp1 levels by targeting SUMO‐2/3. Consequently, this correlation is linked to infarction.^165^ However, other studies demonstrate that oxygen‐glucose deprivation degrades SENP3, resulting in the decline of the binding of Drp1 to mitochondria. Following reperfusion, increased SENP3 leads to the deSUMOylation of Drp1, promoting its binding to mitochondria. This, in turn, causes mitochondrial fragmentation and the release of cytochrome *C*, ultimately leading to neuronal death.^153^ Therefore, Drp1 SUMO modifications play crucial roles in mitochondrial dynamics, and understanding their clear function is essential. In neurodegenerative disorders, extensive mitophagy and dysfunctional mitochondria are consequences of Drp1‐mediated mitochondrial fission. In PD, several proteins associated with the disease, such as PINK1, Parkin, and DJ1, have been observed to directly interact with Drp1 and regulate mitochondrial fission and fusion. Specifically, Parkin stimulates the proteasomal degradation of Drp1, leading to a reduction in mitochondrial fission by decreasing the available amount of Drp1.^152^ Consequently, the loss of Drp1 function contributes to the death of nigrostriatal dopaminergic neurons, which is a characteristic feature of PD. This is because Drp1 is involved in the survival of dopaminergic terminals in the caudate‐putamen region, which is necessary for maintaining their axons.^129^ Furthermore, in patients with AD, there is an increase in Drp1 levels, which interact with Aβ and phosphorylated Tau. This interaction results in the upregulation of mitochondrial fission, leading to neuropathology and cognitive decline.^152^, ^166^ Considering the role of the fission protein Drp1 in mitochondrial function, it is evident that Drp1 plays a crucial role in NDDs.

### 2.3 Effects of abnormal SUMOylation of proteins in HD

HD is a neurodegenerative disorder that causes the progressive degeneration of neurons within the brain, suffering from both movement issues and cognitive decline.^167^ Approximately one in 10,000 individuals suffers from HD, an autosomal dominant neurological disorder.^168^ The beginning of HD usually happens in early adulthood, and the condition is always fatal. It is a hereditary condition caused by a genetic mutation in the huntingtin (Htt) gene. It is characterized by the accumulation of mutant huntingtin proteins (mHtt) within the brain. This pathogenic buildup of mHtt leads to neuronal degeneration, which is the primary pathological feature of HD. Previous studies have indicated this occurs on both Htt and ras‐homolog enriched in the striatum (Rhes) proteins. This SUMOylation has been suggested to contribute to the pathogenesis of HD.^169^, ^170^ While various brain regions are impacted by HD, including the thalamus, cortex, and subthalamic nucleus, the striatum is the most severely afflicted region.^171^ The disease primarily targets the medium spiny projection neurons in the striatum, whereas interneurons are largely unaffected.^172^ Although it has been suggested that transcriptional dysregulation plays a significant role in the pathophysiology of HD, the mechanisms underlying the alteration of gene expression are yet unknown.

#### 2.3.1 Abnormal SUMOylation of Huntington protein in HD

The Huntington protein (Htt protein) in humans is a large protein consisting of 3144 residues (347 kDa) and is involved in many cellular processes.^170^, ^173^ Htt is abundantly expressed in the nervous system as well as other tissues, including the testes.^167^, ^174^ A complete understanding of Htt's function in the brain has not yet been achieved. However, it is believed to play a critical role in regulating neuronal and glial function. Among the known functions of Htt are participation in pre‐ and postsynaptic functions and implications in the regulation of apoptotic signaling, vesicle transport, transcription, and nuclear import.^173^, ^174^, ^175^ Additionally, the Htt protein plays a role in promoting the synthesis and axonal transport of brain‐derived neurotrophic factor (BDNF).^173^, ^175^ BDNF is a critical protein for the growth and survival of neurons, as well as for regulating synaptogenesis and synaptic plasticity, both of which are necessary for learning and memory in the adult CNS.^176^ Htt is probably involved in the formation of cilia, which are tiny, hair‐like structures that assist in regulating the flow of cerebrospinal fluid (CSF) in the brain and contribute to the movement of this fluid within the ventricular system.^175^, ^177^ The Htt protein contains several different domains, including a poly‐Q tract and a proline‐rich region.^178^, ^179^ Despite some knowledge about the structure of the Htt protein, such as that it is mainly composed of alpha‐helices, there is still a lack of understanding of the protein as a whole.^178^, ^179^ One of the crucial regions of the human Htt protein is located at the N‐terminus, which contains a polymorphic glutamine stretch near the proline‐rich region and likely affects turnover by preventing protein aggregation through the regulation of protein–protein interactions (PPI).^174^ Although the solubility of the Htt protein is not fully understood, studies have detected its soluble and aggregated isoforms, such as soluble mutant Htt (excluding exon 1 Htt) and aggregated Htt. These investigations aim to understand how Htt solubility corresponds to the onset and progression of the disease.^180^, ^181^ The formation of toxic, soluble oligomers of Htt during the aggregation process is believed to contribute to HD.^182^

Mutations in the Htt gene have been associated with the development of HD.^167^ Given that HD is an autosomal dominant disease, the loss of function of the Htt protein has long been recognized as a contributing factor to the complex pathology of the disease.^183^ However, the precise molecular mechanisms underlying the pathology of HD are still largely unknown.^184^ The most significant features of HD are the presence of inclusion bodies and the deposition of aggregated protein inside cells. A mutation in the IT15 gene on chromosome 4's short arm causes HD.^185^ Therefore, these protein inclusions primarily consist of N‐terminal fragments of mHtt. Despite being a characteristic feature of the disease, these inclusions are considered to have a protective role rather than being contributors to neurodegeneration. Soluble oligomeric assemblies of huntingtin formed early in the aggregation process are considered potential toxic agents in HD.^186^ The poly‐glutamine moiety of the Htt protein is encoded by the cytosine–adenine–guanine (CAG) trinucleotide repeats in exon 1 of the mutated gene. The variety of CAG repeat lengths in normal people is 7–34. With a length inversely correlated with the age at which the disease first manifests, the CAG repeat is both enlarged and unstable in individuals with HD. HD is produced by repeat lengths greater than 40 glutamines, and juvenile‐onset is always caused by repeat lengths greater than 70 glutamines.^187^ Consequently, as a monogenic disease, one of the key pathogenic causes of HD is associated with the expression of the CAG triplet repeat in the Htt, which leads to the production of mHtt proteins with an expanded poly‐Q domain in the N‐terminus at exon 1.^184^, ^188^ Additionally, there is a significant correlation between the length of the polyQ repeat and the aggregation of protein.^186^ The fragment of exon 1 within the huntingtin protein (Httex1) has been extensively researched and is identified for its occurrence of improper splicing and proteolytic processing. Notably, the first 17 amino acids (Nt17) of exon 1 undergo PTMs that play roles in regulating the cellular functions of the huntingtin protein in both healthy individuals and those with HD.^189^ The length of the poly‐Q tract in Htt has an impact on the protein's PTMs, which in turn affects its subcellular distribution, function, cleavage, and stability.^174^, ^188^ The polyproline (polyP) domain adjacent to the polyQ tract in Htt is responsible for maintaining the protein's solubility, and it is also involved in regulating interactions with vesicle‐bound proteins.^167^

Therefore, the Htt protein undergoes various PTMs, including SUMOylation, ubiquitination, phosphorylation, acetylation, palmitoylation, and proteolytic cleavage, which are believed to have a significant impact on the protein's PPI flexibility.^167^, ^174^ Furthermore, PTMs can impact the subcellular localization of Htt.^174^ While certain PTMs, such as phosphorylation at S13/16 and S42110, may have a protective effect against the toxic impact of mutant Htt, several PTMs are associated with HD pathogenesis and the increased toxicity of mutant Htt. Additionally, myristoylation is another potential protective PTM in Htt, but its levels decrease in the presence of an expanded polyQ, which may serve as a potential protective PTM.^190^ The N‐terminal region of Htt contains a site for both SUMOylation and ubiquitination, adjacent to polyQ and polyproline (polyP) tracts.^167^ When Htt undergoes ubiquitination, it is degraded by proteasomes, whereas the same Lys residues undergo SUMOylation to protect it from degradation.^191^, ^192^ The process of ubiquitination on the pathogenic Htt fragment (Httex1p) inhibits neurodegeneration, whereas the SUMOylation of the pathogenic fragment of the protein is believed to contribute to the exacerbation of neurodegeneration.^182^ The Htt protein contains three important Lys residues, Lys‐6, Lys‐9, and Lys‐15, which are conjugated to SUMO.^174^, ^182^ Both SUMO‐1 and ubiquitin can modify the same Lys residues on Httex1p. Preventing both modifications through Lys mutations reduces HD pathology, suggesting that the role of SUMOylation in HD pathology is more extensive than simply inhibiting the ubiquitination and degradation of Httex1p.^182^ There is evidence suggesting that SUMO‐2 modification plays a role in the pathogenic accumulation of mutant Htt and the progression of HD with age. The accumulation of SUMO‐2‐modified proteins in the insoluble fraction of the postmortem striatum in HD indicates their involvement in the disease. As a result, HD might be influenced in vivo by SUMO‐2‐modified proteins. The striatum, a region significantly affected by HD, has been observed to exhibit an accumulation of SUMO‐2, correlating with the pathological accumulation of Htt.^170^, ^191^ Moreover, Htt undergoes SUMO‐2 modification, and the protein inhibitor of activated STAT 1 (PIAS1) acts as an E3 ligase for Htt SUMOylation. PIAS1, expressed in the brain, specifically enhances SUMO‐1 and SUMO‐2 modifications of Htt, crucial for the aforementioned protein accumulation.^191^ Moreover, when Htt is SUMOylated, it can lead to a toxic accumulation of the protein.^191^ mHtt undergoes SUMOylation via Rhes, resulting in Rhes having a higher efficacy in SUMOylation compared with wild‐type, thereby protecting mHtt from degradation. This process elevates the levels of the soluble cytotoxic form of the protein. Rhes, the small guanine nucleotide‐binding protein (G‐protein), is predominantly found in the striatum.^174^, ^193^ It plays multiple roles in the SUMOylation process, including acting as a SUMO‐E3 ligase, enhancing the efficiency of Ubc9 in SUMOylation mHtt, and promoting cross‐SUMOylation between SAE1, SAE2, and Ubc9.^194^ As mentioned earlier, the interaction between Rhes and mHtt leads to the SUMOylation of mHtt, which has a cytotoxic nature and ultimately results in cytotoxicity. The interaction between Rhes and mHtt triggers the cytotoxic SUMOylation of mHtt, potentially causing cytotoxicity and contributing to the observed localized neuropathology in HD.^193^ The SUMOylation of Htt may lead to a distinct pathway of aggregation that could potentially impact Htt toxicity.^195^ Ubc9, the only identified SUMO‐E2 ligase, SUMOylated mHtt at specific Lys sites (6, 9, 15, and 91).^194^ The SUMOylation of Htt has been suggested to increase the levels of toxic oligomers, and it is also possible that soluble oligomers may have toxic effects.^182^ While most Htt aggregates are found in the cytosol, the accumulation of Htt in the nucleus may enhance its toxic effects. SUMOylation on Htt protein has been found to have paradoxical effects. Subsequently, it reduces the formation of aggregates while its solubility is enhanced.^191^, ^192^ Therefore, the Htt protein can undergo a pathological fragmentation process, leading to the formation of a fragment known as a pathogenic fragment of Htt (Httex1p), which can also be SUMOylated by SUMO‐1. This suggests that SUMOylation might impact the molecular properties and functions of Httex1p. The same Lys residues targeted by SUMOylation might also undergo modification through ubiquitination. The contribution of SUMOylation to HD pathology has been shown to go beyond inhibiting Htt ubiquitination and degradation in a *drosophila* model of HD. SUMOylation of Httex1p intensifies neurodegeneration, whereas ubiquitination of Httex1p appears to counteract it. Additionally, mutations in Lys (K6,9) and arginine (R15) residues, preventing both SUMOylation and ubiquitination of Httex1p, have been shown to improve HD pathology.^182^ Furthermore, research indicates that SUMO‐1 exacerbates the solubility and toxicity of Htt. Considering the information, different outcomes are anticipated depending on which SUMO paralog is involved in Htt conjugation.^170^ Modifying Htt through either SUMO‐1 or SUMO‐2/3 has the potential to regulate HD and provide new opportunities for the development of neuroprotective therapies.^38^, ^170^ Therefore, it is hypothesized that reducing the level of SUMOylated Htt in neurons could be a therapeutic strategy. This reduction could be achieved by lowering the expression of the SUMO‐1 precursor, inhibiting SUMO‐1 ligases, and enhancing isopeptidase activity to facilitate the removal of SUMO‐1.^182^

## 3 EFFECTS OF ABNORMAL PHOSPHORYLATION OF PROTEINS IN NDDs

**FIGURE 4:**
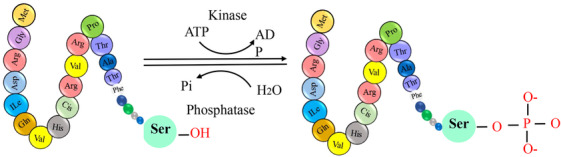
Phosphorylation is a covalent modification that involves the addition of a phosphate group to serine, threonine, tyrosine, and histidine residues by kinase enzymes. Conversely, dephosphorylation, or the removal of a phosphate group, is an enzymatic reaction catalyzed by various phosphatases (PPs).

Phosphorylation is a crucial PTM that significantly influences various essential physiological processes. This complex biochemical process involves a kinase enzyme transferring phosphate groups from molecules like ATP or guanosine‐5'‐triphosphate (GTP) to specific amino acid residues on proteins (see Figure 4). Serine and threonine are the most commonly phosphorylated amino acid residues, with tyrosine phosphorylation being less frequent. Due to their prevalence in protein sequences, serine, and threonine residues are highly susceptible to phosphorylation, which can regulate protein activity, stability, localization, and interactions with other molecules, impacting cellular processes like signal transduction, gene expression, cell cycle progression, and metabolism.^3^, ^196^ Noteworthy is that phosphorylation is a reversible process regulated by phosphatases.^3^ Indeed, the dynamic interplay between phosphorylation and dephosphorylation is a critical mechanism for regulating protein functions. Through phosphorylation, proteins can be switched “on” or “off,” activating or deactivating their functions, respectively.^197^ This process is highly regulated and allows for precise control of cellular processes. In neurons, phosphorylation plays a significant role in various neuronal functions. It is involved in synaptic transmission, regulating the strength and plasticity of neuronal connections. Phosphorylation also influences neuronal growth and development, including axon guidance and dendritic arborization. Furthermore, phosphorylation is crucial for the regulation of microtubule dynamics, which are essential for intracellular transport and structural integrity within neurons.^198^ However, aberrant phosphorylation of proteins is also implicated in the regulation of pathological processes in NDDs. Dysregulation of phosphorylation can contribute to the development and progression of NDDs, including neuroinflammation, which is a hallmark of many NDDs. Abnormal phosphorylation events can lead to the activation of inflammatory signaling pathways and the release of proinflammatory molecules, contributing to neuroinflammation and subsequent neuronal damage.^199^ Understanding the role of phosphorylation and its dysregulation in NDDs is crucial for developing potential therapeutic strategies targeting these processes.

### 3.1 Abnormal phosphorylation of proteins in AD

Phosphorylation plays a significant role in AD. In particular, abnormal phosphorylation is recognized as a crucial PTM implicated in the disease's underlying mechanism.^200^ In AD, the aberrant phosphorylation of essential proteins such as Tau and APP is linked to neurodegeneration. This intricate phosphorylation process plays a crucial role in the progression and onset of the disease. Additionally, proteins other than Tau and APP have been detected in AD. These proteins have been found to directly interact with each other, potentially impacting their aggregation patterns and phosphorylation levels in the disease. The intricate interplay among these proteins, including α‐syn, Tau, and Aβ, could contribute to the development of NDDs. Recent research has uncovered a synergistic interaction among Tau, Aβ, and other proteins associated with AD, providing potential insights into the pathological events linked to the condition. Nevertheless, the exact correlation between Tau, and Aβ in AD remains elusive.^201^, ^202^ For instance, the interplay between Aβ and Tau sets in motion a cascade of events that culminate in neuronal damage. Aβ triggers aberrant phosphorylation of Tau, leading to its p‐Tau and subsequent toxicity within neurons. Likewise, Tau acts as a mediator for the detrimental effects of Aβ toxicity, with their interaction being crucial for the manifestation of harmful consequences. Consequently, the existence of Aβ pathology could instigate alterations in the release and phosphorylation of soluble Tau, ultimately culminating in the aggregation of p‐Tau in an individual's brain over a prolonged duration. This suggests that Tau and Aβ could concurrently exist in pathological locations, underscoring their close association in the progression of the disease.^203^, ^204^ Moreover, the complex interplay between α‐syn, Aβ, and Tau proteins triggers a cascade of events, leading to diverse consequences. This intricate process may be associated with the pathophysiology and onset of cognitive decline. The interaction between α‐syn and Tau significantly enhances Tau phosphorylation through multiple kinases. This suggests that increased α‐syn in AD could accelerate Tau phosphorylation, resulting in elevated levels of phosphorylated Tau and the progression of Tau pathology.^201^, ^202^ These proteins collectively act as biomarkers for AD, highlighting the potential of their interaction as a diagnostic marker for distinguishing AD from other neurodegenerative disorders.^202^

#### 3.1.1 Abnormal phosphorylation of Tau protein in AD

Among the extensively studied PTMs, Tau phosphorylation is a key focus. It greatly influences Tau's solubility, stability, localization, degradation, aggregation, function, and interactions with microtubules. The phosphorylation of Tau can have a dual effect on its function, leading to both beneficial and detrimental outcomes in physiological and pathological conditions, respectively. Therefore, the specific phosphorylation patterns observed at particular residues of Tau carry substantial importance in the realm of tauopathies, including AD.^205^ In its normal state, the Tau protein typically carries 2–3 moles of phosphate per mole, which is essential for its function in microtubule assembly, especially within axons.^206^, ^207^ Consequently, Tau's physiological and pathological roles are intricately linked to the fundamental processes of phosphorylation and dephosphorylation. The complex interplay between Tau's normal and abnormal functions is closely tied to these underlying processes of phosphorylation and dephosphorylation. Preserving the proper functioning of Tau relies on a delicate equilibrium between the enzymes known as kinases and phosphatases, responsible for adding and removing phosphate groups from Tau, respectively. Any disruption in this balance, whether through increased kinase activity or decreased phosphatase activity, culminates in the excessive phosphorylation of Tau. This aberrant phosphorylation subsequently leads to neuropathological consequences in tauopathies.^58^, ^208^ Tau phosphorylation is regulated by three primary classes of kinases (known as Tau kinases) that target its Ser/Thr and tyrosine residues, including proline‐directed kinases (PDPKs), nonproline‐directed kinases (non‐PDPKs), and tyrosine protein kinases.^57^ Tau undergoes phosphorylation by PDPKs at Ser/Thr‐Pro motifs, which refer to Ser/Thr residues positioned immediately before a proline residue. This category encompasses cyclin‐dependent kinase 5 (CDK5), glycogen synthase kinase 3 beta (GSK‐3β), MAPK, and dual specificity tyrosine‐phosphorylation regulated kinase 1A.^57^ Non‐PDPKs are responsible for phosphorylating Tau at motifs containing Ser/Thr‐X, including MAP/microtubule affinity‐regulating kinase 4, casein kinase 1 (CK1), Tau–tubulin kinases, and cyclic AMP‐dependent protein kinase A. Tyrosine protein kinases induce Tau phosphorylation through Fyn, spleen tyrosine kinase, Abl kinases, and Src family kinases. In humans, the longest form of the Tau protein consists of around 85 residues that can undergo phosphorylation. This set of residues comprises 45 serine residues and 35 threonine residues, along with five tyrosine residues, all susceptible to phosphorylation. Significantly, the majority of phosphorylation occurrences take place in distinct domains: residues 172–251 within the proline‐rich domain, residues 244–369 in the MTBR, and residues 368–441 in the C‐terminal tail region.^209^

As the phosphorylation level of Tau increases, its ability to bind microtubules diminishes. While the Tau protein normally stabilizes microtubule bundles, abnormally phosphorylated Tau at specific residues can interfere with its interaction with tubulin. This disruption ultimately leads to microtubule disassembly, resulting in axonal damage and neuronal apoptosis.^210^ The Tau protein's role in these processes highlights its significance in neuronal injury and dysfunction. In addition to disrupting neuronal transport and causing cytotoxicity, p‐Tau can spread to other neurons and different regions of the brain. After becoming hyperphosphorylated, Tau dissociates from microtubules and can be released from their surface. Abnormal phosphorylation of Tau not only disrupts its normal functions but also imparts neurotoxic properties, ultimately leading to neuronal death in AD and other tauopathies. Moreover, abnormally phosphorylated Tau tends to aggregate, potentially forming toxic oligomers and insoluble structures called paired helical filaments (PHFs), eventually contributing to NFts formation.^211^, ^212^ Specifically, the hyperphosphorylation of Tau at certain residues, including pSer‐199, pSer‐202, and pThr‐205, as well as pThr‐212, pThr‐231, pSer‐262, and pSer‐396, has been reported to be implicated in destabilizing microtubules, ultimately leading to neurofibrillary pathology.^213^, ^214^

All six isoforms of Tau exhibit hyperphosphorylation, which leads to microtubule destabilization. Subsequently, they are detected within the structures of PHF and NFt.^215^ It is important to note that 3R and 4R Tau isoforms contribute to the composition of PHFs and NFts, further highlighting their significance in pathological Tau aggregation.^215^ These toxic filaments comprise two smaller filaments, each approximately 10 nm in diameter, intertwining to form periodic structures. The crossover distance between the filaments within the PHFs is typically around 65–80 nm. This pattern of intertwining gives rise to the characteristic appearance of PHFs in AD.^216^ Specific phosphorylation residues on Tau proteins have been found to undergo abnormal phosphorylation during the progression of AD and other tauopathies while remaining unphosphorylated in healthy brains. This process indicates a significant increase (at least threefold) in the phosphorylation of Tau compared with its normal state. Around 50 distinct phosphorylation residues involving serine, threonine, and tyrosine have been detected as abnormally phosphorylated in the AD brain, such as Tyr‐18, Ser‐199, Ser‐202, Thr‐205, Thr‐212, Ser‐214, Thr‐217, Ser‐262, Thr‐231, Ser‐262, Ser‐396 and Ser‐422.^209^, ^217^ Several key residues associated with pathological conditions are prominently located within the MTBR.^209^, ^218^ These residues are predominantly found within PHFs, NFts, and neuropil threads (NTs).^216^ The phosphorylation of specific residues plays a crucial role in influencing the progression and stages of AD. As a result, various site‐specific profiles have been identified in AD, spanning from its early to late stages. Notably, the phosphorylation of Thr‐231, Ser‐396, and Ser‐422 has been recognized as an early event in AD progression. In particular, Ser‐422 may act as a significant indicator for the onset of p‐Tau‐induced pathology and aggregation.^209^, ^217^ In addition, elevated levels of p‐Tau have been observed at key residues, including Ser‐199, Ser‐202/205, and Ser‐422, in the cortex and transentorhinal cortex during advanced Braak stages/VI.^218^ In individuals with AD, the level of phosphorylated Tau at Ser‐396 in the frontal cortex is notably elevated. This increased phosphorylation of Tau at the Ser‐396 residue is likely associated with Aβ and is considered a crucial factor in the progression of AD. Interestingly, the research revealed that pThr‐231 (p‐Tau_231_) displayed substantial correlations with AD pathology, demonstrating a notable increase from early stages to advanced AD and a significant connection with Aβ in the disease.^219^ Interestingly, differences in the levels of p‐Tau at Tyr‐18 or Thr‐231 in the transentorhinal region have been observed to vary across different Braak stages of AD. For example, in Braak stages III/IV, phosphorylation at Tyr‐18 and Thr‐231 is observed in the transentorhinal region, exhibiting a progressive increase. This suggests that Tyr‐18 and Thr‐231 may play a pivotal role in the early stages of AD onset.^218^

Tau protein naturally maintains a stable conformation with minimal tendencies for misfolding. Its structure is comparable to that of phosphorylation at the Thr‐231 paperclip, where both ends of the Tau protein (N‐ and C‐terminal) fold over the MTBR region. This structural configuration acts as a safeguard, helping to inhibit the self‐aggregation of the protein.^220^ Hyperphosphorylation of the Tau protein brings about a conformational change that facilitates its aggregation. Recent research indicates that phosphorylation at Thr‐231 induces the conformational alteration of the Tau protein, leading to a neurotoxic conformation known as cis p‐Tau, which is thought to contribute to the early stages of diverse neurodegenerative disorders, including AD. Studies have shown that cis p‐Tau, distinct from the normal trans p‐Tau conformation, displays exceptional stability and resistance to both microtubule binding and degradation mechanisms.^208^, ^221^ The misfolded p‐Tau, acting as a seed, is associated with specific phosphorylation residues implicated in the pathological seeding of Tau and the progression of AD. Remarkably, Tau seeds exhibit a notable capacity for release from damaged neurons to the extracellular space, utilizing diverse exocytosis mechanisms. This enables them to spread to nearby neurons, displaying prion‐like propagation behavior. For instance, neuronal death and/or the secretion of exosomes serve as the means for the release of Tau seeds. Following their release, these Tau seeds are internalized by other neurons or glial cells via different mechanisms, such as membrane fusion of exosomes.^222^ One crucial role of the Tau seeds lies in their ability to recruit soluble Tau and contribute to the formation of larger abnormal structures.^221^, ^223^ Subsequently, the spread of Tau aggregation correlates with cognitive decline, indicating the worsening of the disease state.^224^ In AD, Tau aggregates are assumed to initially accumulate in the entorhinal cortex and spread to cortical areas during disease progression.^225^ Recent research indicates a substantial accumulation of cis p‐Tau, which initiates and propagates p‐Tau aggregation, leading to a pathological phenomenon known as cistauosis. This process plays a significant role in neuronal death and the advancement of neurodegeneration, particularly in AD and traumatic brain injury (TBI).^208^, ^226^

The abnormal phosphorylation of Tau is closely tied to the progression of insoluble Tau aggregates, which are identified as a hallmark of AD pathology. This interconnection implies that the accumulation rate of insoluble Tau aggregates may indeed play a significant role in the observed cognitive decline in individuals affected by AD.^227^ Abnormal phosphorylation of Tau neurotoxicity contributes to cognitive decline through loss of synaptic function and neuronal death.^228^ Impaired synaptic function is a crucial factor in the cognitive decline seen in AD and may appear early in the disease process.^229^ In healthy human brains, Tau is present in approximately 55 and 70% of presynaptic and postsynaptic compartments, respectively. Its presence actively contributes to regulating the proper functioning of synapses in healthy conditions.^229^ Elevated levels of p‐Tau have been detected in the postmortem tissues of individuals with AD, particularly in the hippocampus and entorhinal cortex. This accumulation significantly disrupts mitochondrial dynamics, causing a decline in dendritic proteins, loss of dendritic spines, and synaptic function. These effects ultimately contribute to impairments in learning and memory that are associated with the hippocampus. Moreover, p‐Tau exhibits a significant propensity to accumulate at synaptic terminals in the hippocampus. This accumulation at synapses can have detrimental effects on synaptic plasticity, eventually resulting in the loss of synapses.^230^, ^231^ The correlation between the abundance of NFts and the decline of cognitive function, coupled with the loss of synapses, suggests a potential causal role for p‐Tau. Additionally, the soluble form of Tau that precedes NFt formation demonstrates a distinct link to synaptic defects, highlighting its ability to interfere with normal synaptic function. Notably, in individuals with AD, elevated pathological Tau could intensify synaptic impairment and accelerate cognitive decline.^231^, ^232^ On the other hand, phosphorylation of Tau at Ser396/404, referred to as PHF‐1, significantly accumulates within mitochondria during the aging process, particularly in synaptic mitochondria in the hippocampal. This accumulation of Tau PHF‐1 inside mitochondria leads to extensive damage, implying a potential correlation with synaptic dysfunction and cognitive decline in the elderly individual. Consequently, the occurrence of phosphorylated Tau PHF‐1 within mitochondria could potentially serve as an early indicator of neurodegeneration in AD.^212^, ^233^

In a physiological state, Tau goes through a continuous cycle of phosphorylation and dephosphorylation to maintain its functionality. However, an imbalance favoring increased phosphorylation diminishes Tau's binding to microtubules, leading to the aggregation of p‐Tau.^221^ Tau undergoes dephosphorylation through the action of several protein phosphatases (PPs). Among these PPs, PP2A is the primary phosphatase responsible for the majority of Tau dephosphorylation. Additionally, other phosphatases such as PP1, PP2B, PP2C, and PP5 also contribute to this process.^58^ In the brains of AD patients, there is a substantial decrease in the activity of PP2A, causing an enhancement of p‐Tau levels and NFt in the brain. The reduced activity of PP2A in AD may be attributed to several factors, including changes in the posttranslational state of its catalytic domain and a decrease in PP2A expression.^234^, ^235^ Peptidyl‐prolyl cis/trans isomerase NIMA‐interacting 1 (Pin1) has been identified as a regulatory factor for PP2A activity, demonstrating its ability to enhance the dephosphorylation of Tau by PP2A. Importantly, Pin1 is actively associated with abnormal phosphorylation patterns observed in AD.^235^, ^236^ As a result, PP2A and Pin1 are recognized as crucial factors that have the potential to inhibit or reverse the hyperphosphorylation and aggregation of Tau, offering promising insights into the understanding of AD and related disorders.

Pin1 plays a crucial role in modulating the conformation of the Tau protein by isomerizing phosphorylated serine/threonine‐proline motifs, particularly Thr‐231. Therefore, Pin1 facilitates the dephosphorylation of Tau by PP2A, thereby regulating its phosphorylation state and restoring its functionality. Its catalytic activity enables the isomerization of Tau at pSer/Thr‐Pro motifs, restoring Tau's microtubule‐binding capability and promoting microtubule assembly. Pin1's catalytic activity facilitates the transition from the conformation of cis p‐Tau231 to trans isoform, enabling the restoration of functional Tau that, by restoring its microtubule‐binding capability, supports neuronal structure and transport while inhibiting the toxic effects associated with cis p‐Tau.^235^, ^237^ Pin1 has also been reported to exhibit a slightly stronger association with phosphorylation at Ser‐202 and Thr‐205.^236^ Pin1's malfunction in AD is attributed to various factors, including phosphorylation and mutations, resulting in the accumulation of p‐Tau231 in a pathogenic cis conformation. Afterwards, this inactivation contributes to the accumulation of p‐Tau231 in the neurotoxic cis conformation, initiating cistauosis and neurodegeneration, occurring even before the formation of tangles in AD.^235^, ^237^ Death‐associated protein kinase 1 (DAPK1) exerts a direct impact on Pin1 by phosphorylating Ser‐71 within its catalytic active site, leading to the inhibition of its enzymatic function.^238^ In addition, DAPK1 also exhibits the capacity to phosphorylate Tau at Thr‐231, Ser‐262, and Ser‐396, which potentially contributes to p‐Tau aggregation and neuronal death. DAPK1 is a crucial player in diverse neuronal injury models, and mounting evidence suggests an association between DAPK1 and the risk of AD. Accordingly, the activation of DAPK1 is implicated in the neurodegenerative processes associated with AD in the brain.^239^

#### 3.1.2 Abnormal phosphorylation of the APP in AD

Phosphorylation of APP occurs continuously in neurons under normal physiological conditions. APP undergoes phosphorylation at specific residues, involving threonine, serine, and tyrosine, situated across its cytoplasmic and extracellular domains of the protein.^240^ Phosphorylation plays a regulatory role in modulating the function and localization of APP.^241^ Furthermore, phosphorylation plays a pivotal role in modulating the proteolytic processing of APP and the associated enzymes, which is closely associated with the pathogenesis of AD.^241^, ^242^ This finding emphasizes the importance of phosphorylation in influencing the regulation of Aβ generation. It not only affects the subcellular distribution of APP but also modulates the enzymatic activities of the relevant secretases involved in its processing.^242^ Therefore, the modification of APP processing is considered to be a crucial factor in the development of AD pathology.^243^ Among the 10 phosphorylated residues recognized in APP695, the cytoplasmic domain exhibits phosphorylation at Tyr‐653, Thr‐654, Ser‐655, Thr‐668, Ser‐675, Tyr‐682, Thr‐686, and Tyr‐687, while the remaining two phosphorylated residues are located in the ectodomain, including Ser‐198 and Ser‐206. In its physiological state, Ser‐198 stands out as the predominant phosphorylation site in the ectodomain of APP, exhibiting a higher phosphorylation level in comparison with Ser206 site.^68^, ^75^, ^242^ The abnormal processing of APP, leading to the accumulation of Aβ peptides, contributes to neurodegeneration and cognitive decline in AD. Numerous phosphorylation sites on APP have been reported to be associated with the development of AD, such as Ser‐26, Ser‐675, Thr‐668, and Tyr‐682.^75^, ^244^, ^245^ Fyn tyrosine kinase (Fyn TK) has been found to phosphorylate APP at Tyr‐682 in AD neurons, indicating its involvement in the phosphorylation process. Following phosphorylation at Tyr‐682, APP undergoes relocation into acidic neuronal compartments, leading to the generation of neurotoxic peptides of Aβ. Notably, there is an enhanced interaction between Fyn TK and the Tyr‐682 residue of APP. Increased phosphorylation of Tyr‐682 has been observed in the neurons of AD patients, and those with elevated phosphorylation levels also exhibit heightened Fyn TK activity.^240^ The enzymatic noncanonical cleavage of APP through meprin β produces truncated Aβ2–40/42 peptides with a strong tendency to aggregate, suggesting that increased activity of this enzyme might play a role in the Aβ generation in AD. Phosphorylation of APP at Ser675 disrupts APP processing, promoting meprin β and reducing α‐secretase activity during APP processing.^243^

The significance of phosphorylation at Thr668 becomes particularly noteworthy when observed in models and brain samples of AD. Phosphorylation at Thr668 occurs exclusively in the brain and is regulated by various kinases, including JNK, CDK5, and GSK‐3β.^242^, ^246^ The activation of DAPK1 has also been found to be correlated with the phosphorylation of APP at T668. Heightened levels of DAPK1 exhibited a clear association with the augmentation of APP phosphorylation. Taken collectively, these findings indicate that DAPK1 contributes to the amplification of APP phosphorylation and the amyloidogenic alteration of APP.^247^ This phosphorylation event is critical for the interaction between AICD and Fe65, suggesting its contribution to neurodegeneration in AD. Fe65 modulates APP processing and contributes to the generation of Aβ peptides. Phosphorylation on residue Thr668 of APP is believed to be crucial for regulating APP's neuronal‐related activities, potentially influencing APP metabolism.^244^, ^246^ The remarkable significance of the phosphorylation of Thr668 residue on APP has garnered significant attention, owing to its prevalent presence in AD and its influential role in modulating the cleavage of APP, highlighting its essential contribution to APP's functionality.^242^, ^244^ Furthermore, the brains of individuals with AD have been found to have significant upregulation of phosphorylated C‐terminal fragments of APP (APP‐CTFs) at Thr668, as well as elevated levels of GSK‐3β and Tau phosphorylation in the brain.^242^ Evidence also suggests that Thr‐668 phosphorylation plays a regulatory role in APP cleavage by BACE1.^244^ On the contrary, the phosphorylation of certain residues within some forms of Aβ has been detected in AD. For instance, there is a variant of Aβ that undergoes phosphorylation at the Ser‐26 residue. This variant is primarily found within intraneuronal deposits during the initial stages of AD. The phosphorylated Aβ at Ser‐26 forms a distinctive oligomeric structure that accumulates within specific intracellular compartments associated with granulovacuolar degeneration. It is worth noting that phosphorylated Ser‐26 oligomers display heightened neurotoxicity compared with other forms of Aβ.^248^

### 3.2 Effects of abnormal phosphorylation of proteins in PD

Phosphorylation is a significant PTM that plays a vital role in PD. Hence, abnormal phosphorylation has been observed in several key proteins linked to PD, including α‐syn, Tau, ubiquitin, Rab10, 14‐3‐3 proteins, and Drp1. The phosphorylation of these proteins plays a crucial role in the progression of the disease (Table 1).^249^, ^250^ This intricate interplay between different proteins’ pathologies highlights the complexity underlying the clinical presentation and progression of PD and related neurodegenerative conditions. Among these proteins, α‐syn and Tau are particularly significant, as their phosphorylation is closely associated with the formation of LBs and NFts, the hallmark features of PD. The presence of phosphorylated α‐syn fibrils in LBs and Lewy neurites is a defining characteristic observed in the histology of PD.^251^, ^252^, ^253^ However, in a healthy brain, the majority of α‐syn exists in its unphosphorylated form, which is considered essential for its optimal function.^254^ On the other hand, emerging evidence suggests that NFts could have significant implications for the pathogenesis of PD. Research indicates that p‐Tau and the formation of NFts may contribute to the development and progression of PD. Furthermore, studies have revealed a potential synergistic effect of α‐syn pathology and p‐Tau in influencing the clinical characteristics of PDD.^253^, ^255^ Moreover, the interplay between α‐syn phosphorylation and Tau pathogenicity is intricate and impactful within neurodegeneration in PD. Notably, these two proteins mutually influence each other's aggregation and solubility, forming a detrimental feed‐forward loop. This interaction is believed to play a crucial role in the initiation and progression of PD.^250^

**TABLE 1:** A list of proteins with abnormal phosphorylated in Parkinson disease.

Protein name	The protein normal function	Kinases	The residue abnormal phosphorylation	The phosphorylated protein abnormal function	References
Ubiquitin	Ubiquitin, present in the outer mitochondrial membrane, conjugates to substrate proteins on a lysine residue through the coordinated actions of E1, E2, and E3 enzymes.	PINK1	Ser‐65	The interaction between Ser65‐phosphorylated ubiquitin and parkin initiates a significant conformational change in parkin. These ubiquitin chains, when phosphorylated at Ser65, exhibit resistance to deubiquitination through various enzymes. Deficiencies in ubiquitin phosphorylation‐dependent mitophagy are increasingly linked to the pathogenesis of PD.	^272^, ^273^
Rab10	Rab10 serves various functions in axons, dendrites, and synapses. It plays a crucial role in neuronal polarization, dendritic branching, and vesicle transport in axons and presynaptic regions.	LRRK2	Thr‐73	The phosphorylation of Rab10 at the Thr‐73 residue (pThr73–Rab10) can hinder ciliogenesis in holinergic interneurons. pRab10 is primarily found in abundance at the presynaptic terminal. In addition, higher levels of pT73–Rab10 are probably linked to more severe PD progression.	^249^, ^274^
14‐3‐3 proteins	The 14‐3‐3 proteins (have seven isoforms) constitute a remarkably conserved protein family intricately involved in numerous pivotal cellular processes, including transcription, neuroprotection, regulation of apoptosis, and metabolism.	CK1 and CK2	Ser‐232	Phosphorylation at ser‐232 in 14‐3‐3 reduces its protective effects. This phosphorylation is elevated in the brains of individuals with PD and contributes to neurodegeneration in the disease. In addition, they found LBs in PD.	^275^
Drp1	As a mitochondrial fission protein, Drp1 plays a crucial role in mitochondrial dynamics.	CDK5	Ser‐616	Phosphorylation of Drp1 at Ser‐616 is implicated in PD and contributes to mitochondrial dysfunction, a key feature of PD pathogenesis. This phosphorylation probably induces excessive mitochondrial fission, leading to the loss of dopaminergic neurons within the substantia nigra in PD.	^276^, ^277^, ^278^

Abbreviations: PINK1, PTEN‐induced putative kinase 1; LRRK2, leucine‐rich repeat kinase 2; CK1 and CK2, casein kinases 1 and 2.

#### 3.2.1 Abnormal phosphorylation of the α‐syn protein in PD

The phosphorylation of α‐syn stands out as the predominant and pivotal PTM in PD, among the various PTMs identified for the protein. Consequently, it assumes a crucial role in synucleinopathies, specifically PD, influencing the folding of α‐syn and leading to aggregation. For instance, around 90% of phosphorylated α‐syn was detected within LBs. However, in normal neuronal conditions, only a small portion (under 5%) of soluble α‐syn is phosphorylated.^256^ Recent studies have suggested a strong association between PD and later stages and a notable elevation in the levels of phosphorylated α‐syn. These findings highlight the critical significance of phosphorylated α‐syn as a potential biomarker for the advanced stages of PD.^257^ α‐Syn is phosphorylated at the C‐terminal tail, with specific phosphorylation sites identified at serine residues (Ser‐87 and Ser‐129) as well as tyrosine residues (Tyr‐125, Tyr‐133, and Tyr‐136). Among these sites, Ser‐129 is extensively phosphorylated in synucleinopathies, exhibiting a specific association with PD.^258^, ^259^ Multiple types of kinases, such as polo‐like kinase 2 (PLK2), CK1 and CK2, and the G‐protein‐coupled receptor kinase 5, are known to phosphorylate α‐syn at the Ser129 residue. Among these kinases, PLK2 has been identified as a key kinase in Ser‐129 phosphorylation in neurons. Although, the precise involvement of all these kinases in the pathogenesis of α‐synucleinopathy and their connection to cellular toxicity resulting from phosphorylation remain incompletely comprehended.^100^, ^260^ The phosphorylation of Ser129 significantly impacts the conformational structure of α‐syn. This modification induces noteworthy changes in the unstructured C‐terminus, primarily resulting from the formation of notable salt bridges. These salt bridges connect pS129, which carries a negative charge, with Lys residues carrying a positive charge present in the relevant region.^258^ Hence, numerous studies have consistently demonstrated a significant correlation between the phosphorylation of α‐syn at the serine 129 residue and the formation of LBs. These investigations have unveiled a remarkable increase in phosphorylation levels of Ser‐129 (pSer129), rising from approximately 4% under normal physiological conditions to a substantial 90% within the LBs identified in PD. This compelling evidence strongly suggests that pSer129 plays a crucial role in the pathogenesis of the disease and underscores its association with the pathological state.^258^, ^261^ While the exact connection between Ser‐129 phosphorylation and LB formation remains uncertain, various research findings have presented conflicting views on its role in PD. Consequently, studies have shown that phosphorylation at Ser‐129 can both impede and promote α‐syn aggregation. Phosphorylation at Ser‐129 is suggested to possess the capability to enhance the formation of inclusions. Nevertheless, recent research suggests that pSer129 may have the capability to diminish seed aggregation and impede the aggregation process leading to the formation of amyloid structures.^261^, ^262^ While the majority of phosphorylation sites in α‐syn are typically found in the C‐terminal region, recent research indicates that a tyrosine residue (Tyr39) located at the N‐terminus may also undergo phosphorylation. This phosphorylation event is phosphorylated by the c‐Abl protein TK. Phosphorylation of Tyr‐39 (pTyr39) has been shown to probably intensify α‐syn aggregation, contributing to the pathological process in PD.^251^, ^263^ Although, the phosphorylation of Tyr‐39 residue via c‐Abl may have a protective effect on α‐syn and potentially protect α‐syn from degradation through the autophagy and proteasome pathways within cortical neurons in PD brains.^251^, ^264^ pTyr39 restructures the fibril core of α‐syn by engaging the entire N terminus, leading to pathological fibril formation. Consequently, this alteration modifies the electrostatic characteristics of the N terminus, which in turn stabilizes several Lys residues and creates a hydrophilic channel within the core.^251^

#### 3.2.2 Abnormal phosphorylation of Tau protein in PD

Tau abnormal phosphorylation is indeed a significant contributing factor to neurodegeneration in PD. The neurodegeneration of nigrostriatal dopaminergic cells is a key feature in PD, and the onset of this degeneration may be linked to the aggregation of p‐Tau. Therefore, p‐Tau could potentially play a crucial role in both the early and late stages of PD development.^265^, ^266^ As a result, numerous studies have established a correlation between p‐Tau and neuronal damage, suggesting its involvement in the mechanisms of degeneration in PD. Accumulating evidence also demonstrates the involvement of p‐Tau and the formation of NFts in the postmortem brain samples of individuals with PD. Furthermore, in PD, the extent of Tau pathology has been associated with cognitive decline. NFts have been noted to manifest varying occurrences in postmortem examinations of individuals diagnosed with PD. It is crucial to emphasize that not all examined samples exhibited NFts. Consequently, the presence of NFts has been possibly correlated with the onset of cognitive impairment and dementia in certain cases.^265^ Extensive research suggests that phosphorylated Tau, characterized by prion‐like features, exhibits a broader distribution across multiple regions of the brain compared with α‐syn. This observation implies that pathogenic Tau may play a pivotal role in steering the overall neurodegeneration observed in PD.^267^

Hyperphosphorylated Tau proteins not only contribute to protein aggregation but also exhibit a tendency to interact with α‐syn. This interaction leads to mutual fibrillization, eventually resulting in the formation of LBs. Therefore, the presence of hyperphosphorylated Tau is closely associated with the pathological development of LB formation.^268^ Hence, the accumulation of phosphorylated‐Tau has gained recognition as a significant and possibly initial pathogenic determinant in PD, playing an essential role in its early stages and overall development.^266^ Multiple studies have detected a few specific residues on the Tau protein phosphorylated in postmortem samples from individuals with PD, particularly Ser‐202, Ser‐262, Ser‐396, and Ser‐404, as well as Thr‐205. Moreover, compelling research indicates a significant enhancement in hyperphosphorylated Tau at Ser‐202, Ser‐262, and Ser‐396/404 levels in the striatum of individuals affected by PD. Furthermore, earlier investigations have established the presence of hyperphosphorylated Tau at Ser‐396 in synaptic‐enriched fractions in the fractions in the frontal cortex of PD samples.^91^, ^269^ Recent evidence suggests that there is a significant increase in the concentration of hyperphosphorylated Tau at Thr‐181 (p‐Tau181) in the plasma of individuals diagnosed with PD. This observation indicates that the elevated levels of p‐Tau181 have the potential to serve as a valuable biomarker for cognitive decline in PD. Moreover, it shows promise as a noninvasive and reliable way to track the progression of cognitive impairment in PD patients. Nonetheless, these initial results provide a foundation for future investigations and potential therapeutic interventions.^270^, ^271^ Although abnormal Tau phosphorylation is commonly recognized as a key phosphorylated protein implicated in the pathology of PD, other essential proteins such as ubiquitin, Rab10, 14‐3‐3 proteins, and Drp1 have also been identified in diverse research investigations (refer to Table 1 for further details). Therefore, understanding the mechanisms underlying the impact of p‐Tau and its interaction with other pathological proteins, as well as the phosphorylation of other proteins associated with PD, could provide valuable insights into PD pathology.

### 3.3 Effects of abnormal phosphorylation of proteins in HD

Recent studies have shed light on the intricate abnormal phosphorylation occurring in HD. One noteworthy finding is the discovery of abnormal phosphorylation involving the Htt protein and the Tau protein in the brains of individuals with HD. Phosphorylation has been consistently observed to occur in the N‐terminal region of Htt. Therefore, this likely implicates the complex involvement of phosphorylation in the disease pathology. Furthermore, research has revealed an increase in Tau phosphorylation in HD brains, highlighting its potential involvement in disease progression.^279^, ^280^ Additionally, current investigations have unveiled extensive pathological inclusions in HD samples, exhibiting abnormal phosphorylation of Tau. Notably, some of these inclusions colocalize with mHtt, suggesting a potential involvement of these two pathogenic factors in the disease progression.^281^ These findings draw attention to the critical role of phosphorylation events in understanding the mechanisms underlying disease progression.

#### 3.3.1 Abnormal phosphorylation of the Huntingtin protein in HD

Multiple phosphorylation sites have been detected within the Htt protein, such as Thr‐3, Ser‐13, Ser1‐6, Ser‐116, Ser‐421, Ser‐536, Ser‐434, Ser‐1181, and Ser‐1201.^173^, ^282^, ^283^ Some of the phosphorylation sites have a beneficial role in protecting cells from the harmful effects of expanded polyQ‐induced toxicity and modulating neuronal toxicity. However, any disturbance in the phosphorylation pattern of these sites triggers the aggregation of mHtt, thereby inducing alterations in phosphorylation patterns associated with the disease. The phosphorylation of the Httex1 region has been shown to play crucial regulatory roles.^283^ Extensive research into the Nt17 domain of Httex1 has yielded compelling evidence highlighting phosphorylation events at specific serine and threonine residues. Particularly noteworthy are Thr‐3, Ser‐13, and Ser‐16, which have been identified as pivotal phosphorylation sites within this domain. Phosphorylation at Ser13 and Ser16 not only affects the structure of the protein but also plays a crucial role in influencing its aggregation propensity and subcellular localization. These phosphorylation events hold considerable importance in the context of the disease.^279^ Recent studies have focused on investigating the effects of phosphorylation at positions 13 and 16 on the Httex1 region. These investigations have consistently revealed compelling evidence that phosphorylated serine residues at positions 13 and 16 actively mitigate protein aggregation. Moreover, the phosphorylation of these serine residues, either individually or in combination, leads to mutant Httex1 fibrils exhibiting a heightened propensity for localization within the nuclear compartment.^279^, ^284^ Studies on clinical samples of HD have revealed a significant decrease in the levels of phosphorylation at residue Thr‐3 in mHtt. When Thr‐3 is phosphorylated, it has a stabilizing effect on the α‐helical conformation of Nt17. Research indicates that phosphorylated Thr‐3 predominantly occurs on full‐length Htt, influencing its aggregation tendencies and pathogenic characteristics. In other words, phosphorylation at Thr‐3 actively inhibits the aggregation of mHttex1. Nevertheless, this suggests that phosphorylation at Thr‐3 plays a significant role in reducing these pathological protein structure processes in Httex1.^285^, ^286^

The IκB complex (IKK) is detected as a crucial kinase in Htt phosphorylation. Specifically, IKK is responsible for directly phosphorylating the Ser‐13 residue of Htt, which could potentially lead to the subsequent phosphorylation of the Ser‐16 residue via IKK. This suggests a potential cross‐talk between these residues’ phosphorylation, indicating their probable interconnected roles in the regulation of Htt pathology. Upon phosphorylation of these residues, a cascade of events is initiated, including acetylation of nearby Lys residues, ultimately facilitating the clearance of Htt via both the proteasome and lysosome.^282^, ^286^ However, the expansion of the polyQ repeat length in mHtt adversely affects the efficiency of phosphorylation at Ser‐13, which is associated with HD. This reduced efficiency could have significant implications for PPI and directly contribute to the accumulation of mHtt.^285^, ^286^

#### 3.3.2 Abnormal phosphorylation of Tau protein in HD

Nowadays, HD could potentially be categorized as a secondary tauopathy, as evidenced by the identification of essential tauopathic features in the postmortem brain tissue of HD patients. These features include misfolding, hyperphosphorylation, and the presence of NFts and NTs. Notably, the advanced stages of HD show the additional presence of p‐Tau insoluble aggregation.^280^, ^287^ Specifically, the presence of sarkosyl‐insoluble Tau and its abnormal phosphorylation patterns in HD displayed a strikingly different isoform.^281^, ^288^ Recent studies have revealed notable increases in Tau phosphorylation throughout the progression of HD. This abnormal phosphorylation of Tau has been observed in postmortem samples of individuals with HD, specifically at sites including Ser199, Thr205, and Ser‐396/Ser‐404. Interestingly, some of these phosphorylation sites are also found to be implicated in AD.^289^ In recent studies, investigations into HD postmortem samples have revealed a noteworthy observation despite: the presence of p‐Tau abnormalities, the level of phosphorylation in HD appears to be relatively weak compared with the extensive phosphorylation typically observed in AD.^280^, ^289^. In the context of HD, it has been observed that Tau pathology in transplanted neural tissue exhibits impaired Tau splicing in exons 2, 3, and 10. Specifically, impairment in Tau splicing in exon 2 was detected in the putamen region of HD brain samples. These abnormal splicing events, along with hyperphosphorylation of Tau, occur early in the disease and may influence the aggregation of mHtt.^281^, ^288^, ^290^ During a study, it was observed that Tau pathology could appear in previously healthy neural tissue transplanted into the brains of individuals with HD. Specifically, two patients with HD exhibited the presence of hyperphosphorylated Tau in the transplanted tissue, specifically at the phosphorylation sites of Ser‐202 and Thr‐205 residues. It is interesting to note that hyperphosphorylated Tau was found to spread to healthy neurons.^289^, ^291^ Hence, Tau may have a high propensity to spread to other regions of the brain. The localization of phosphorylated Tau oligomers displayed distinct patterns in HD brains. Tau hyperphosphorylation was detected in specific brain regions, including the putamen, striatum, cortex, and hippocampus, within HD. Notably, a significant elevation of p‐Tau was specifically observed in the putamen.^287^, ^289^

## 4 EFFECTS OF ABNORMAL ACETYLATION OF PROTEINS IN NDDs

**FIGURE 5:**
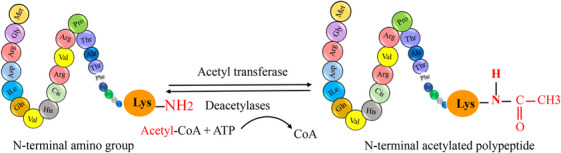
Acetylation is a reversible modification that is regulated by acetyltransferase enzymes. Acetyltransferases utilize acetyl CoA as a cofactor to add an acetyl group (COCH_3_) to the ε‐amino group of lysine side chains, while deacetylases remove acetyl groups from lysine side chains.

Acetylation is a crucial biological process that modifies the structure of proteins by adding acetyl groups, thereby regulating the viability and function of all mammalian cells. Various chemical groups, including hydroxyl, thiol, or amino groups, can undergo acetylation.^292^ Although acetylation was first observed as a modification on histone proteins in the nucleus, it also occurs on several nonhistone proteins, playing a critical role in governing how cells respond and communicate to various internal and external stimuli. During translation, approximately 80–90% of proteins undergo N‐terminal acetylation at the polypeptide chain's N‐terminus.^293^ Enzymes called N‐terminal acetyltransferases (NATs) facilitate the transfer of an acetyl group from acetyl‐coenzyme A to the α‐amino acid of proteins, leading to extensive and irreversible N‐terminal acetylation (refer to Figure 5).

These modifications could affect the protein's half‐life, the process by which proteins attain their tertiary structure, or their intracellular localization.^294^ Alterations in acetylation have been associated with disruptions in protein clearance pathways in NDDs, such as autophagy and the proteasome.^295^ For example, research has shown that elevated p300/CBP enzyme activity changed autophagic flow, leading to excessive Tau protein release in transgenic AD mice.^296^ Additionally, it was demonstrated that the HDAC inhibitor 4b, which primarily inhibits the HDAC1 and HDAC3 enzymes, enhances cognitive performance in a transgenic HD mouse model by removing Huntingtin protein via lysosome and proteasome pathways. Protein aggregation, a pathophysiological characteristic of neurodegenerative disorders, can also lead to altered proteostasis.^297^ Various factors, including mutations, oxidative stress, or modified PTM, can induce protein misfolding and the aggregation of aggregate‐prone proteins. It is believed that these aggregates affect the anatomical and functional functions of neurons, which helps to promote the pathogenic process of diseases such as AD, PD, and HD.^297^ Since most of these disease‐related proteins can become acetylated, changes to acetylation may play a role in protein misfolding and the formation of protein aggregates in NDDs. Nonetheless, most research focusing on the relationship between abnormal phosphorylation and protein aggregation has emphasized this aspect.^298^ Here, we highlight our understanding of the changes in acetylation that occur in some important proteins that assemble in NDDs.

### 4.1 Effects of abnormal acetylation proteins in AD

AD is the most prevalent kind of senile dementia. It is a neurodegenerative illness that is typified by permanent memory loss and cognitive decline. Aβ plaques and Tau NFts are the major pathological components of AD.^88^ Epigenomic dysregulation has emerged as a potentially significant link between neurodegeneration, disease pathological characteristics, and aging, based on advances in our understanding of the dynamics of chromatin architecture during normal aging and during neurodegeneration.^299^, ^300^ AD can be classified into two main types based on the symptom onset. Early‐onset AD (EOAD) is an autosomal dominant form of AD that occurs due to mutations in genes such as APP, presenilin 1 (P1), or presenilin 2 (PSEN2), which are involved in A‐peptide synthesis.^301^ On the other hand, late‐onset AD (LOAD) is the most prevalent form, accounting for over 95% of cases. Although certain genetic variations have been associated with an increased risk of developing LOAD, there is currently no known causative gene for this type of AD.^301^ The exact cause of LOAD is still not fully understood. However, it has been suggested that a combination of epigenetic and environmental factors plays a significant role in the development of the disease. These factors interact with various loci and may collectively increase the risk of developing LOAD.^302^ Dysregulation of these processes has been observed in both aging models and NDDs such as AD. Histone modifications, in particular, have been associated with important cognitive functions, such as learning memory, and synaptic plasticity. As a result, extensive research efforts have been focused on understanding the changes in histone modifications in AD and exploring potential therapeutic approaches that target these modifications and the enzymes associated with them to address the pathophysiology of AD.^303^ Histone acetylation plays a crucial role in altering the interaction between DNA and histone by neutralizing the positive charge of Lys residues. This process weakens the association between DNA and histones, facilitating the access of the transcriptional machinery to gene promoters.^304^ Additionally, transcription factors (TFs) are recruited by histone acetylation, which also makes it easier for them to bind to promoter regions.^305^ Histone acetylation is essential for several processes in the human brain, such as synaptic plasticity, memory consolidation, and memory creation.^306^ Notably, histone acetylation has been shown to play a role in the development of excitatory and long‐term memory synapses in the hippocampal regions, which are essential for many types of synaptic plasticity, such as long‐term potentiation (LTP).^307^ Several studies utilizing animal models have explored the role of histone acetylation in learning, memory, and synaptic plasticity. Following contextual fear conditioning, which is associated with impaired learning and memory, increased levels of H3 acetylation have been identified at the Bdnf promoter region in the hippocampal regions of adult mice.^308^ Furthermore, in genes associated with memory consolidation, elevated histone acetylation has been observed in regions targeted by the TF nuclear factor κB (NF‐κB).^309^ Moreover, studies have shown that adult mice expressing a mutant form of cAMP response element‐binding protein (CREB) binding (CBP) with reduced histone acetyltransferase (HAT) activity exhibit impaired long‐term memory consolidation. Intriguingly, when HAT activity is restored or HDAC inhibitors (HDACi) are utilized, the memory deficits are reversed. Additionally, CBP mutant mice demonstrate reduced long‐term memory formation and impaired LTP, highlighting the critical role of histone acetylation in memory and synaptic plasticity processes.^310^ Recent therapeutic use of HDACi has shown promising results in the treatment of cognitive disorders.^311^

### 4.2 Effects of abnormal acetylation proteins in PD

α‐Syn can undergo acetylation at its N‐terminus.^312^ Tryptic digestion and liquid chromatography with tandem MS were used to analyze cytosolic and pathologically deposited α‐syn from patients with dementia with LBs and PD, specifically postmortem hippocampal, temporal, cingulate, and prefrontal cortical gray matter regions. The results demonstrated that this α‐syn is constitutively N‐terminally acetylated. NAT are responsible for the common PTM known as N‐terminal acetylation, which occurs in approximately 85% of eukaryotic proteins.^313^ Acetylation of histone H3 decreases when α‐syn expression is increased, and α‐syn‐induced DNA damage is reversed by sodium butyrate (NaB), an HDAC inhibitor.^314^ Moreover, α‐syn decreases H3 acetylation, and the PD‐associated mutations A30P and A53T in α‐syn may exacerbate this effect. Both cellular and transgenic drosophila models are protected against α‐syn‐mediated neurotoxicity by the HDAC inhibitors NaB and SAHA.^315^ In rat models of PD, it has been shown that the HDAC inhibitor VPA protects against α‐syn‐induced neurotoxicity.^316^ Notably, α‐syn and H3 do not directly correlate, suggesting that histone masking may be responsible for the α‐syn‐induced decrease in histone acetylation.^317^ Nuclear α‐syn operates through a similar mechanism by binding to the gene promoter region of peroxisome PGC‐1α, a TF of the mitochondria that, when dysfunctional, leads to oxidative stress and mitochondrial dysfunction in the pathophysiology of PD; in addition, it inhibits histone acetylation and suppresses PGC‐1α expression. Additionally, α‐syn diminishes histone acetylation, likely by reducing p300 levels and its HAT activity.^318^

#### 4.2.1 Acetylation of histones in the tissues of Parkinson patients

Histone acetylation, alone or in conjunction with other PTMs, plays a crucial role in governing gene expression and regulating other genomic activities by attracting or repelling chromatin regulatory protein complexes. This significantly effects on some critical cellular processes and the progression of illnesses.^319^ KATs normally acetylate histones to decrease their binding to DNA, relax chromatin, and switch on gene transcription; HDACs deacetylate histones to cause chromatin condensation and inhibit gene transcription.^320^ Furthermore, histone acetylation permits it to interact with TFs and proteins containing the bromodomain, which increases the number of regulatory factors.^321^ A new investigation compared two sets of fibroblasts from individuals with hereditary PDLRRK2 G2109S and idiopathic PD.^322^ G2109S and R1441G mutations in the LRRK2 gene are responsible for an autosomal dominant form of familial PD.^89^ Histone acetylation is reduced in idiopathic PD fibroblasts as opposed to controls, despite an increase in the acetylation levels of total proteins. Anacardic acid (AA), a HAT inhibitor, is advantageous for idiopathic PD fibroblasts, whereas the nonspecific HDAC inhibitor trichostatin A is detrimental.^322^ Idiopathic PD cells may have hyperacetylation of proteins due to impaired mitophagy and defective mitochondrial accumulation, which leads to reduced nicotinamide adenine dinucleotide (NAD^+^) synthesis and subsequently lowers SIRT activity. Upon SIRT inhibition, class I and II KDACs such as HDAC2, HDAC3, and HDAC4 become more active; overall HDAC activity, particularly HDAC6 activity, is reduced. Therefore, to restore the balance of histone acetylation levels and reduce cell damage in PD, HAT inhibitors or selective inhibitors targeting HDAC2, HDAC3, or HDAC4 are preferable to nonspecific HDAC inhibitors.^323^

#### 4.2.2 PD‐related neurotoxins and acetylation of histones

When various PD‐related neurotoxins such as 1‐methyl‐4‐phenyl‐1, 2, 3, 6‐tetrahydropyridine/1‐methyl‐4‐phenylpyridinium (MPTP/MPP+), dieldrin, rotenone, and paraquat are applied to DA neurons, one of the main epigenetic changes that occur is hyperacetylation of histones H3 or H4. For example, treatment with MPTP in mice or MPP+ in neuronal cells leads to increased Lys acetylation of histones, accompanied by reductions in the levels of HDAC1, HDAC2, HDAC4, HDAC6, and SIRT1.^324^ MPP+ treatment reduces SIRT1 expression, increases H3K14 acetylation, and triggers transcription of the hypoxia‐inducible factor‐1 promoter.^325^ Mice treated with MPTP also exhibit increased H3 acetylation in the striatum, consistent with the study's findings. Dieldrin, a toxin linked to the pathophysiology of PD, induces time‐dependent hyperacetylation of H3 and H4 via CBP accumulation, with hyperacetylation serving as a precursor to dieldrin‐induced neurotoxicity. Additionally, the HAT inhibitor AA significantly reduces dieldrin‐mediated DA neuronal death.^326^ Paraquat induces histone acetylation by downregulating HDAC4 and HDAC7, leading to increased H3, rather than H4, hyperacetylation, and subsequent death of DA neuronal cells.^327^ Rotenone triggers neurodegeneration by boosting p53 expression and elevating H3K9 acetylation through decreased SIRT1 levels. Resveratrol, a SIRT1 activator, mitigates p53 production and cell damage induced by rotenone.^328^ HAT inhibitors such as curcumin, AA, and garcinol have been shown to alleviate l‐DOPA‐induced dyskinesia in 6‐hydroxydopamine‐induced PD animal models. This suggests that a combination of l‐DOPA with a HAT inhibitor may hold therapeutic potential for managing l‐DOPA‐induced dyskinesia in PD patients.^329^

### 4.3 Effects of abnormal acetylation proteins in HD

CBP is a protein that is widely expressed in various tissues. It functions as an acetyltransferase and serves as a transcriptional coactivator for several TFs, including the CREB. CBP binds to specific genes and enhances their transcriptional activity. In addition to its role in gene regulation, CBP is involved in multiple neural functions, such as the stress response, modulation of synaptic plasticity, and facilitation of synaptic communication. Moreover, CBP is implicated in various other biological processes.^330^, ^331^ CBP exerts its gene transcription activation capability by utilizing its inherent HAT activity. This enzymatic function enables CBP to add an acetyl group to specific Lys residues, such as H4K8ac or H3K27ac, thereby modulating the accessibility of DNA to the transcriptional machinery. When CBP function is impaired, it hampers the process of transcription by hindering the recruitment of the basal transcription machinery to the promoter region. Additionally, it disrupts the proper acetylation levels of histones and alters the chromatin structure within neurons. Emerging evidence indicates that abnormal transcriptional regulation contributes to the pathogenesis of HD. In this context, the sequestration of CBP has been implicated in the disruption of CRE‐mediated transcription, as observed in HD transgenic model R6/2. Studies have demonstrated the aggregation of CBP in this model. Interestingly, separate investigations have shown that reducing CBP aggregation leads to an improvement in the condition of mice in the same HD model.^330^ These findings highlight the potential significance of CBP and its aggregation in the development and progression of HD, providing a basis for further research and potential therapeutic interventions. It has been widely recognized for over a decade that the sequestration of CBP by mHtt results in significant neuronal transcriptional dysfunction. The poly‐Q stretches present in mHtt physically interact with CBP, effectively impairing its transcriptional coactivator function and its intrinsic HAT activity. Consequently, the sequestration of CBP by mHtt expression leads to the hypermethylation and hypoacetylation of histone proteins, resulting in the subsequent transcriptional dysfunction observed in neurons affected by HD. These specific interactions and transcriptional dysfunctions can be attributed to pathological epigenetic modifications that contribute to the disease pathology of HD.^331^

#### 4.3.1 Transcriptional dysregulation in HD

Strong evidence from recent research indicates that transcriptional dysregulation is a key underlying mechanism in the pathophysiology of HD.^332^, ^333^ Throughout several HD models, transcriptional dysregulation is a key early event in HD pathogenesis.^334^ The structure of the Htt protein is comparable to that of known TFs because of the poly‐Q repeats in its N‐terminal region. The expansion of these repeats causes abnormal cleavage by caspases. The fragments penetrate the nucleus and create nuclear aggregates that could interfere with transcription. Since mutant Htt interacts with many TFs, including p5327, TATA‐binding protein (TBP), CBP, and Sp1, Htt nuclear aggregates may sequester TFs and cause transcriptional dysregulation.^335^

#### 4.3.2 HDAC inhibitors in HD

Histone‐modifying activity is possessed by various Htt‐interacting proteins. CBP is a coactivator at different promoters and carries an acetyltransferase domain. It has been demonstrated that CBP and other TFs are confined to Htt aggregates in the brains of HD patients and transgenic mice.^336^ Moreover, CBP overexpression reduces cell mortality caused by poly‐Q.^337^ It was shown by Steffan and colleagues that the acetyltransferase domain of CBP and p300/CBP‐associated factor are directly bound by Htt exon 1 with 51 glutamines that contain the poly proline domain (Httex1p 51Q).^337^ The acetylation of histone H4 is reduced in the presence of mutant Htt, according to in vitro research, indicating that Htt's direct interaction with the acetyltransferase domains suppresses the acetylation of histone proteins. Gardian et al.^338^ treated another transgenic mouse strain, HD‐N171‐82Q, with phenylbutyrate, an HDAC inhibitor, when symptoms appeared. When given to 75‐day‐old HD‐N171‐82Q mice, phenylbutyrate boosted survival and reduced ventricular hypertrophy and striatal atrophy. On motor function, weight loss, or the generation of Htt aggregates, there was no difference. After being treated with phenylbutyrate, the striatum showed an increase in histone H3 and H4 acetylation and a decrease in histone H3 methylation. The HD‐N171‐82Q striatum treated with phenylbutyrate was subjected to microarray analysis, which revealed that certain genes were elevated and others were downregulated. Given that treatment was started after symptoms appeared, it is encouraging that transgenic mice displayed an overall improvement in their condition, even if phenylbutyrate did not increase the expression of mutant‐Htt‐downregulated genes. It is unclear how HDAC inhibitors work therapeutically. The most straightforward explanation that can be imagined is one in which the downregulation of particular genes brought on by mHtt is rectified by the injection of HDAC inhibitors. However, limited research using HDAC inhibitors on transgenic mice suggests that this is not the case. Alternatively, the advantages of HDAC inhibitors could come from higher global gene expression. If so, HDAC inhibitors may have advantages not only for HD but also for other NDDs. HDAC inhibitors have also demonstrated promise in AML transgenic animal models, schizophrenia, and ischemia.^339^

## 5 EFFECTS OF ABNORMAL UBIQUITINATION OF PROTEINS IN NDDs

**FIGURE 6:**
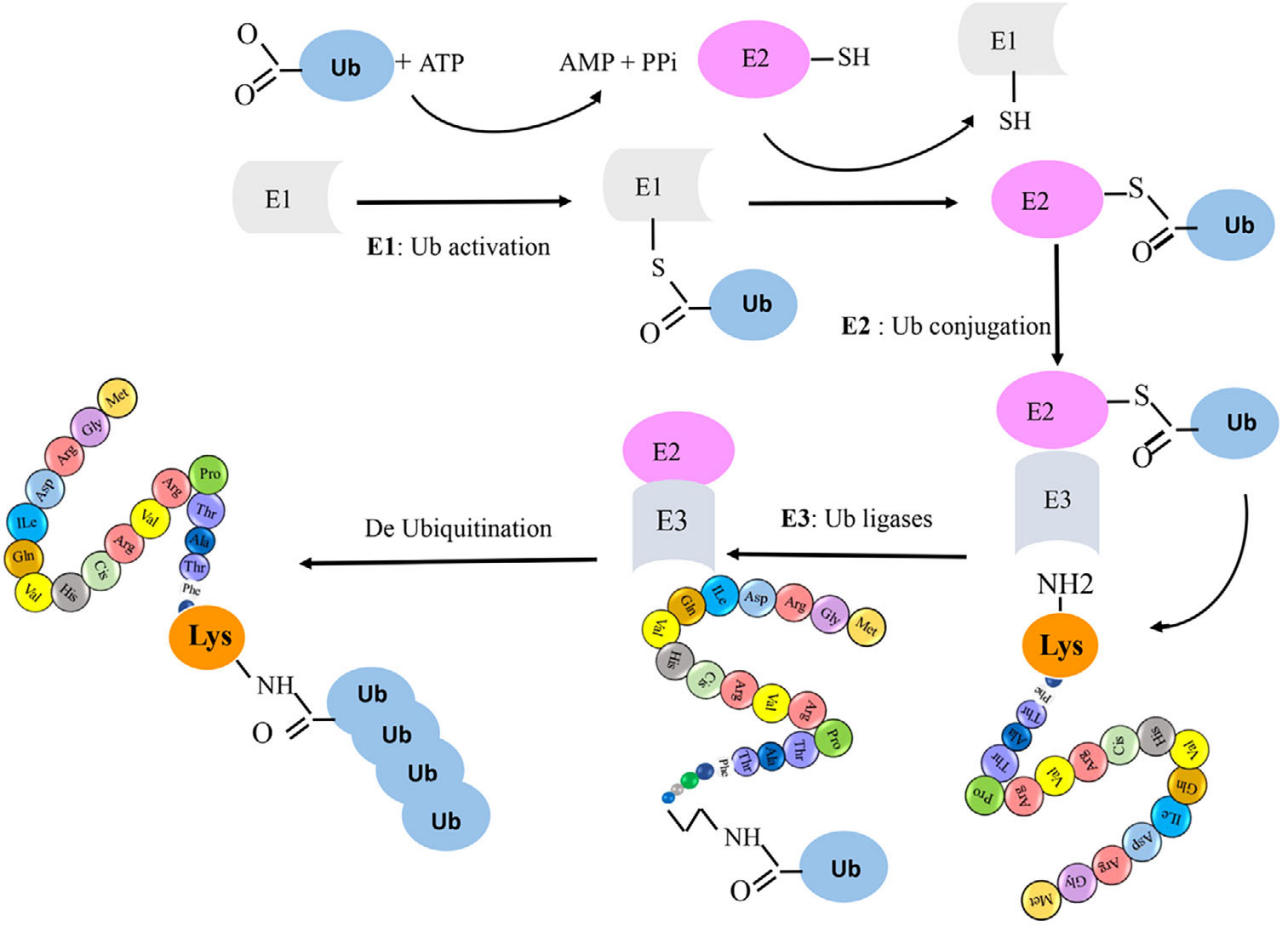
Ubiquitination is one type of reversible posttranslational modification (PTM) of proteins. Ubiquitin is covalently attached to proteins through a peptide bond between its C‐terminal glycine and the Nε lysine residues of target proteins. The process involves three enzymes: an activating enzyme (E1), a conjugating enzyme (E2), and an ubiquitin ligase (E3). Ubiquitin can be removed from the protein by deubiquitinating enzymes (DUBs).

Ubiquitination is a pivotal PTM that exerts significant control over protein function, stability, localization, PPIs, and protein degradation. This intricate process involves the covalent attachment of ubiquitin, a 76‐amino acid protein, to specific Lys residues in target proteins. Moreover, ubiquitination can also occur on the side chains of serine, threonine, and cysteine residues. It is crucial to note that ubiquitination is a reversible process and relies on a cascade of enzymes, including the ubiquitin‐activating enzyme (E1), the ubiquitin‐conjugating enzyme (E2), and the ubiquitin‐protein ligase (E3).^3^, ^340^ Initially, ubiquitin is activated by the E1 enzyme through ATP hydrolysis. This involves adenylation of the carboxyl group at the C‐terminus of ubiquitin and its subsequent transfer to a cysteinyl residue on the E1 enzyme. Following this activation step, ubiquitin is conjugated to a cysteinyl residue on the E2 enzyme. The final step in ubiquitin transfer involves the formation of an isopeptide bond between ubiquitin and a Lys residue on the substrate protein. This transfer can occur directly from the E2 enzyme or after the formation of a thioester bond between ubiquitin and the E3 ligase enzyme. These three main steps, activation, conjugation, and ligation, collectively form the process of ubiquitination (Figure 6). Among these steps, E3 ligases play a critical role, as they are diverse enzymes responsible for specifically recognizing target proteins. Through bioinformatics analysis, it has been revealed that the human genome encodes more than 600 E3 ligases.^86^

Furthermore, it is important to note that ubiquitin can also bind to various sites and undergo modifications by other PTM processes such as acetylation and phosphorylation. In this context, specific E3 ligases and deubiquitinating enzymes (DUBs) assume crucial regulatory roles analogous to “pens” and “erasers,” respectively. E3 ligases control the structure and characteristics of ubiquitin chains, while DUBs regulate the removal of ubiquitin modifications. Together, they form complex and diverse signal transduction systems that intricately regulate essential biological processes within living organisms.^86^ Indeed, the close association between E3 ligases, DUBs, and protein degradation pathways, including proteasomal degradation, autophagy, and ER phagocytosis, suggests that any dysfunction in these enzymes may contribute to the development of NDDs. E3 ligases and DUBs also play a pivotal role in conveying intricate biological signals that regulate various cellular processes associated with the pathology of NDDs. For instance, they are involved in the regulation of mitochondrial function, which is crucial for maintaining cellular energy homeostasis and preventing neuronal damage. Additionally, E3 ligases and DUBs influence processes like excitotoxicity, which refers to the excessive stimulation of neurons leading to cell death, and immune inflammation, which plays a significant role in the neuroinflammatory response observed in NDDs.^86^ Understanding the intricate interplay between E3 ligases, DUBs, and the associated cellular processes holds great promise for unraveling the mechanisms underlying NDDs. This knowledge may pave the way for the development of novel therapeutic strategies to combat these debilitating conditions.

Indeed, ubiquitination serves as a critical crossroad between cell survival and death, owing to its potent ability to clear malfunctioning or cytotoxic proteins, thereby enabling cells to overcome proteotoxic stress.^341^ Ubiquitin molecules can be attached to a substrate protein either as a single ubiquitin (monoubiquitination) or as a chain of ubiquitin molecules (polyubiquitination). The PTM of a protein through monoubiquitination plays a crucial role in modulating various cellular events. On the other hand, poly‐ubiquitination involves the addition of multiple ubiquitin moieties to previously conjugated ubiquitin molecules. This process ultimately leads to protein degradation through the UPS. Polyubiquitination marks the targeted proteins for recognition and subsequent degradation by the proteasome, a vital cellular machinery responsible for protein turnover and clearance.^342^ Indeed, the removal of ubiquitination is primarily facilitated by DUBs. These enzymes play a crucial role in maintaining the balance and dynamics of ubiquitin signaling within cells. By cleaving the ubiquitin moieties from target proteins, DUBs effectively reverse the ubiquitination process. This coordinated interplay between Ub‐modifying enzymes and DUBs is essential for maintaining normal cellular activities and the proper functioning of various cellular processes.^343^, ^344^

Ubiquitination is a highly specific and regulated process that is essential for cellular homeostasis. By controlling the addition and removal of ubiquitin molecules, these enzymes contribute to the fine‐tuning of protein stability, localization, interactions, and degradation. This delicate regulation of ubiquitin signaling ultimately impacts critical cellular pathways, including protein quality control, immune responses, DNA repair, and cell cycle progression.^341^, ^345^ NDDs are frequently associated with proteinopathies, disease‐related gene mutations, or disrupted protein homeostasis. The UPS and the autophagy–lysosomal pathway play crucial roles in degrading misfolded proteins to prevent their buildup within cells. Both of these degradation pathways rely on the modification of specific targets with ubiquitin.^346^ The UPS plays a crucial role in maintaining protein quality control and cellular homeostasis. It serves as the primary pathway for the degradation of misfolded proteins, and dysfunctions in the UPS have been associated with intracellular protein aggregation, cytotoxicity, and cell death. In NDDs such as AD, PD, and HD, the UPS can play a significant role in the accumulation of aggregated misfolded proteins and the formation of inclusion bodies, contributing to the pathogenesis of these disorders.^342^ During the aging process, the delicate balance between ubiquitination and deubiquitination plays a critical role in maintaining neuronal homeostasis and influencing neuronal survival. As aging progresses, there is a general decline in proteasomal degradation and autophagy, which are the key mechanisms for clearing misfolded proteins and maintaining cellular health. Consequently, this age‐related decline in protein clearance mechanisms leads to the accumulation of potentially neurotoxic protein aggregates. In NDDs such as AD and PD, the aging process contributes to an increased accumulation of Aβ and Tau proteins in AD and α‐syn in PD. Similarly, in HD, there is an accumulation of superoxide dismutase 1 and mHtt, while in ALS, TAR DNA binding protein‐43 aggregates are observed. These protein aggregates are associated with neuronal dysfunction and cell death, further emphasizing the impact of aging on the development and progression of NDDs.^86^, ^346^

Abnormal ubiquitination proteins have been observed in NDDs, suggesting that dysfunction of the UPS plays a role in the development of these disorders. Several specific proteins involved in the UPS have been implicated in the pathogenesis of NDDs. In AD, abnormalities in Parkin (an E3 ligase), ubiquitin C‐terminal hydrolase L1 (UCH‐L1) (a DUB), HRD1 (an E3 ligase), and the C‐terminus of Hsc70‐interacting protein (CHIP) (an E3 ligase) have been reported. Similarly, in PD, abnormalities in Parkin (E3 ligase), UCHL1 (DUB), CHIP (E3 ligase), and TRAF6 (E3 ligase) have been observed (Tables 2, 3). These abnormalities suggest that dysfunctions in the UPS contribute to the accumulation of disease‐associated proteins and the pathogenesis of PD. Furthermore, it has been reported that abnormalities in CHIP (E3 ligase) play an important role in HD. CHIP dysfunction may lead to impaired protein quality control and clearance mechanisms, contributing to the accumulation of misfolded proteins and the progression of HD. Overall, the dysregulation of specific UPS components in NDDs highlights the significance of the UPS in maintaining protein homeostasis and underscores its involvement in the pathogenesis of these neurodegenerative disorders.^341^ The UPS is a critical pathway for protein degradation, responsible for the breakdown of approximately 80% of intracellular proteins in eukaryotic cells. In many NDDs, there is a common pathological feature characterized by the presence of intracellular ubiquitin‐positive inclusions formed by aggregate‐prone and neurotoxic proteins. The dysregulation of ubiquitin‐dependent processes extends beyond a single UPS system and encompasses various aspects of cellular function. This includes alterations in gene transcription and translation that are influenced by ubiquitin‐mediated signaling pathways. Dysregulated ubiquitination can also affect protein quality control and maturation, protein trafficking, the turnover of mitochondria, and the regulation of PPIs.^341^, ^347^

**TABLE 2:** E3s in neurodegenerative diseases.

Disease	E3 ligase	Interactor/substrate	Functional implications of the E3 ligase	References
PD	CHIP	α‐Syn	CHIP is a cochaperone that has been implicated in PD due to its involvement in mediating the degradation of α‐syn aggregates. α‐Syn is a protein associated with the formation of LBs, which are characteristic pathological features of PD. CHIP has been shown to facilitate the clearance of α‐syn aggregates in vivo, indicating its potential therapeutic significance in PD. However, it is worth noting that CHIP's involvement in PD extends beyond α‐syn degradation. There is evidence suggesting that CHIP may also be involved in the degradation of tyrosine hydroxylase, an enzyme required for the synthesis of dopamine, which is significantly affected in PD. This dual role of CHIP raises considerations regarding its applicability as a therapeutic target in PD, as targeting CHIP for α‐syn degradation could potentially compromise the levels of tyrosine hydroxylase and further disrupt dopamine production.	^348^
PD	CHIP	G2385R LRRK2	The G2385R variant of LRRK2 in PD has significant implications. Studies have shown that this variant inhibits LRRK2 dimerization, destabilizes the protein, and enhances its kinase activity. Moreover, the G2385R variant promotes LRRK2 protein turnover by increasing binding affinity to Hsc70 and CHIP, leading to enhanced intracellular degradation of LRRK2.	^349^
PD	Nedd4	α‐Syn	Nedd4 promotes the degradation of α‐syn by ubiquitination, which is essential for preventing the accumulation of α‐syn in intraneuronal inclusions characteristic of PD.	^350^
PD	Parkin	NEMO	NEMO is a protein that functions in the NF‐κB signaling pathway and plays a crucial role in the regulation of inflammation and immune responses. The interaction between Parkin and NEMO suggests that Parkin may have a role in regulating cellular stress responses and maintaining mitochondrial quality control.	^351^
PD	Parkin	RIPK1	Parkin affects RIPK1 in Parkinson's disease by mediating site‐specific ubiquitination of RIPK1, which influences signaling pathways like NF‐κB and MAPK and plays a role in regulating cell death processes associated with PD. Furthermore, Parkin prevents the formation of the RIPK1–RIPK3 complex by promoting polyubiquitination of RIPK3, thereby negatively regulating necroptosis in inflammation and tumorigenesis.	^352^, ^353^
AD	CHIP	BACE1	BACE1 is regulated by the E3‐ligase CHIP. CHIP stabilizes APP by promoting the ubiquitination and proteasomal degradation of BACE1, thereby reducing Aβ production BACE1, a key enzyme involved in the production of Aβ in AD, which is regulated by the E3‐ligase CHIP. CHIP stabilizes APP by promoting the ubiquitination and proteasomal degradation of BACE1, thereby reducing Aβ production.	^354^, ^355^
AD	CHIP	Tau	CHIP and Hsp70 play crucial roles in regulating Tau ubiquitination, degradation, and aggregation. CHIP acts as an ubiquitin ligase for Tau and is involved in Tau degradation, while Hsp70 can reduce the accumulation of insoluble Tau aggregates. The balance between CHIP and Hsp70 levels is essential for maintaining normal Tau physiology and preventing Tau aggregation and toxicity in AD.	^356^
AD	CRL	BRI2/BRI3	BRI2 and BRI3 are substrates for a CRL that targets them for degradation, which inhibits APP processing and Aβ oligomerization in AD.	^86^, ^357^
AD	Itchy	Tap73	The E3 ubiquitin ligase Itch plays a crucial role in regulating the proteasome‐dependent degradation of TAp73, a protein involved in cell proliferation and apoptosis.	^358^
AD	Mdm2	Cav1.2	Mdm2 plays a crucial role in the ubiquitination and degradation of Cav1.2. Overexpression of Mdm2 has been shown to significantly decrease Cav1.2 protein levels and enhance its ubiquitination, leading to the degradation of Cav1.2.	^359^, ^360^
AD	PIAS1	Endogenous APP	Endogenous APP serves as a substrate that can be SUMO‐modified by PIAS1 in the hippocampus, affecting various processes related to AD. The interaction between PIAS1 and APP fragments like C83 and C99, which contain the AICD, influences Aβ degradation and pathological symptoms in AD.	^85^
HD	CHIP	mHtt	The soluble mHtt protein is associated with various ubiquitin‐modifying enzymes, affecting its interaction with deubiquitinating enzymes and subsequent degradation pathways. Specifically, ubiquitination of mHtt has been linked to reduced toxicity and increased clearance by the proteasome. Enzymes like UBE2W are involved in ubiquitinating mHtt at its N‐terminus, potentially working in conjunction with E3 ligases like CHIP to target mHtt for degradation. CHIP, a U‐box E3 ligase, can ubiquitinate polyQ expanding proteins such as mHtt, inhibiting aggregation and cell death, especially when coexpressed with the Hsc70 chaperone.	^361^
HD	FBXW7	HSF1	In HD, elevated expression of the protein kinase CK2α and the E3 ligase component FBXW7 promotes the phosphorylation‐dependent degradation of HSF1. FBXW7 depletion has been shown to suppress increased invasion, indicating that HSF1 is a critical FBXW7 substrate involved in this process.	^362^
HD	HACE1	NRF2	HACE1 plays a significant role in regulating NRF2, a key TF involved in antioxidant responses. Research indicates that HACE1 promotes NRF2 accumulation by supporting its protein synthesis independently of its E3 ligase activity. Additionally, decreased HACE1 expression in HD brains leads to Nrf2 degradation and inhibits Nrf2 activity, impacting redox balance and antioxidant response mechanisms. HACE1 is essential for astrocyte mitochondrial function and promotes the stability of NRF2, highlighting its importance in maintaining cellular redox homeostasis.	^363^, ^364^
HD	HOIP	mHtt	In HD, the protein HOIP interacts with mHtt by promoting linear polyubiquitin formation on mHtt. Silencing HOIP reduces mHtt ubiquitination, increasing interaction between mHtt and the transcription factor Sp1, resulting in transcriptional dysregulation, aggregation, and proteotoxicity. This effect is counteracted by silencing OTULIN, a deubiquitinase that hydrolyzes linear polyubiquitin. The polyQ expansion in mHtt influences its ubiquitination, affecting degradation and aggregation.	^365^
HD	PIAS1	Htt	PIAS1 affects the accumulation of mHtt in HD by modulating the levels of SUMO‐modified proteins, including Htt. PIAS1 appears to function at the protein homeostasis level rather than modulating Htt gene expression directly, influencing PTMs of proteins like Htt, Tau, and α‐syn, which are linked to regulating their abundance and association with cellular clearance networks.	^366^
HD	UBE3A	mHtt	UBE3A is an E3 ligase that plays a critical role in the ubiquitination and degradation of the mHtt protein in HD. Overexpression of UBE3A reduces mHtt aggregation and promotes mHtt degradation via the proteasome through its role in K48‐linked ubiquitination.	^361^, ^365^

Abbreviations: LRRK2, leucine‐rich repeat kinase 2; NEMO, NF‐kappa‐B essential modulator; RIPK1, receptor‐interacting serine/threonine‐protein kinase 1; CRLs, cullin‐RING E3 ubiquitin ligases; Nedd4, neural precursor cell‐expressed developmentally downregulated protein 4; MDM2, mouse double minute 2; Cav1.2, cardiac l‐type calcium channel; HSF1, heat shock factor 1; FBXW7, F‐box/WD repeat‐containing protein 7; HSF1, heat shock transcription factor 1; HACE1, HECT domain and ankyrin repeat‐containing E3 ubiquitin protein ligase 1; HOIP, HOIL‐1‐interacting protein; UBE3A, ubiquitin‐protein ligase E3A.

**TABLE 3:** DUBs in neurodegenerative diseases.

Disease	DUB	Interactor/substrate	Functional implications of the DUB	References
PD	USP8	α‐Syn	α‐Syn is a substrate for the deubiquitinase Usp8, which interacts with and deconjugates K63‐linked ubiquitin chains on α‐syn, prolonging its half‐life and increasing its toxicity. Usp8 is upregulated in neurons with Lewy body pathology, and its increased expression can lead to the accumulation of α‐syn, which is associated with PD.	^367^
PD	USP10	P62	USP10 plays a significant role in PD by interacting with p62. Research indicates that USP10 has dual functions in promoting protein aggregation and aggresome formation. It interacts with p62, enhancing p62‐induced protein aggregation.	^368^
PD	USP24	ULK1	Specifically, USP24 has been found to negatively regulate ubiquitination levels and stability of the ULK1 protein, thereby reducing autophagy flux. This interaction between USP24 and ULK1 is significant in the context of PD, where defects in autophagy have been linked to dopaminergic neuron degeneration.	^369^, ^370^
PD	USP33	Parkin	Parkin, a protein involved in mitophagy, is regulated by USP33, a deubiquitinase enzyme. USP33 deubiquitinates Parkin, specifically targeting Lys435‐linked ubiquitin chains on Parkin, leading to its deubiquitination. This process affects Parkin's stability and translocation to damaged mitochondria, influencing mitophagy, and can lead to inhibiting mitophagy.	^371^
AD	OTUB1	Tau	OTUB1 is a key player in modulating Tau aggregation, stability, and ubiquitination processes related to NDDs like PD. Furthermore, Otub1 has been implicated in promoting the progression of PD through the deubiquitylation of Tau protein.	^86^, ^372^
AD	USP46	AMPARs	AMPARs are a substrate for the deubiquitinating enzyme USP46. USP46 regulates AMPAR protein stability by deubiquitinating AMPARs, which protects them from degradation and maintains their surface expression at synapses in neurons. Knockdown of USP46 leads to elevated AMPAR ubiquitination and a reduction in surface AMPARs at synapses. USP46 is also involved in regulating AMPAR‐mediated excitatory synaptic transmission.	^373^, ^374^
HD	ATXN3	Beclin‐1	Beclin‐1 is a protein that plays a role in autophagy, and in the context of HD, a mutation in the gene encoding ataxin‐3 (ATXN3) has been implicated in the pathogenesis of the disease. Beclin‐1 may be involved in the clearance of mutant ataxin‐3, and ataxin‐3 may play a role in the regulation of ubiquitination and autophagy.	^375^

Abbreviations: USP24, ubiquitin specific peptidase 24; USP10, ubiquitin‐specific protease 10; USP10, ubiquitin specific peptidase 10; AMPARs, alpha‐amino‐3‐hydroxy‐5‐methyl‐4‐isoxazolepropionic acid receptors; DUB, ubiquitin‐specific protease 8 (USP8); USP33, ubiquitin specific peptidase 33; ULK1, Unc‐51 like autophagy activating kinase 1; OTUB1, ubiquitin thioesterase; USP46, ubiquitin specific peptidase 46; USP46, ubiquitin specific peptidase 46; AMPARs, α‐amino‐3‐hydroxy‐5‐methyl‐4‐isoxazolepropionic acid receptor; ATXN3, ataxin‐3.

## 6 EFFECTS OF ABNORMAL GLYCOSYLATION OF PROTEINS IN NDD

**FIGURE 7:**
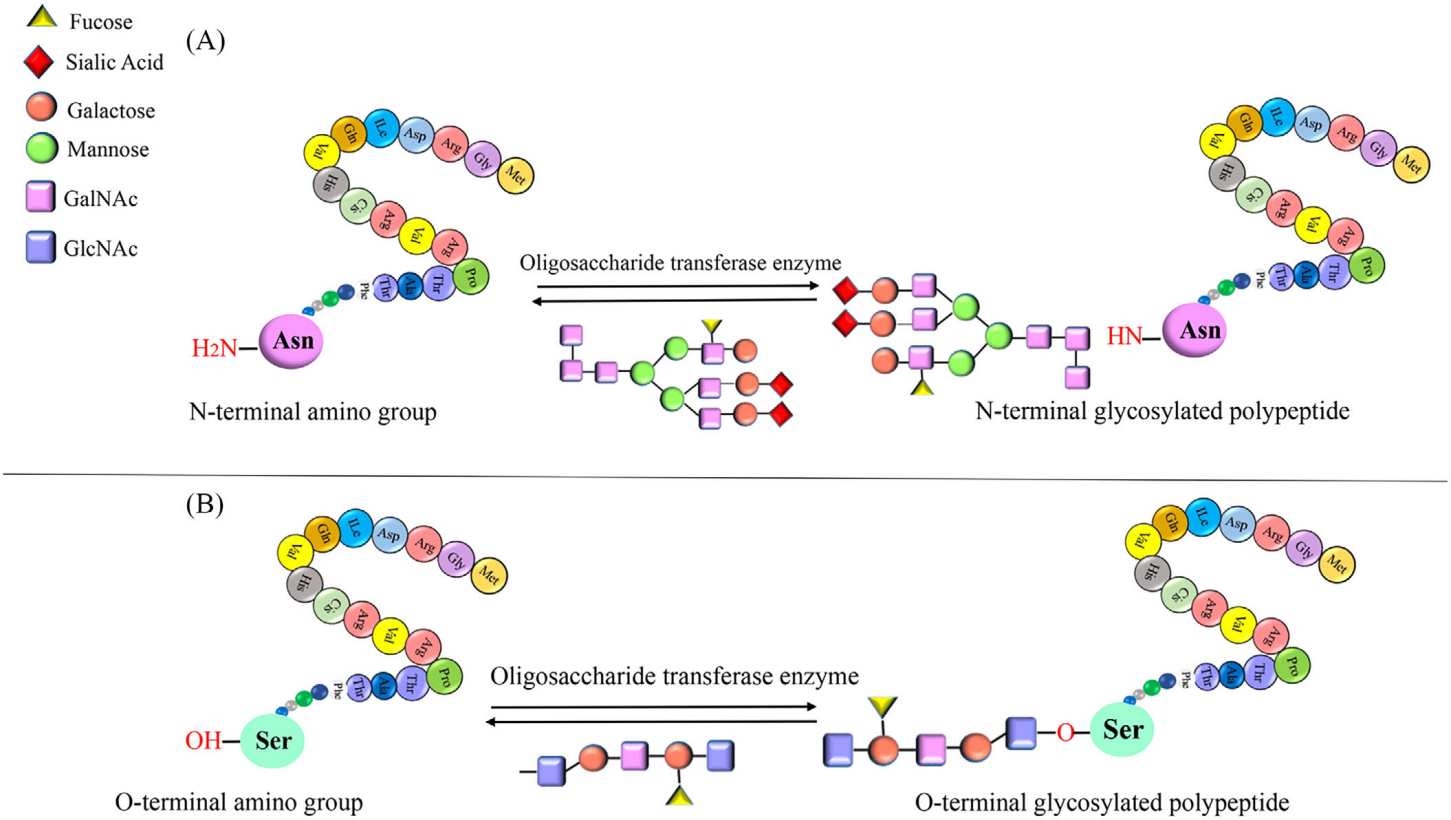
Glycosylation is an enzymatically reversible covalent modification involving glycosyltransferases (GTs) and glycosidases. Glycosylation entails the addition of carbohydrate units to serine, threonine, asparagine, and tryptophan residues in proteins and lipoproteins. (A) N‐linked glycosylation involves the attachment of oligosaccharides to asparagine residues. (B) O‐linked glycosylation consists of adding a sugar to the hydroxyl group (OH) of serine or threonine residues.

Glycosylation is a critical PTM process that involves the covalent attachment of glycans or carbohydrates to proteins, lipids, and/or other glycans. This complex mechanism has a profound effect on cellular function. Glycosylation can take various forms, characterized by the type of linkage and the molecule to which the glycan attaches. Notably, glycosylation is a fundamental process, with nearly 50% of mammalian proteins undergoing this modification. This highlights its essential role in cellular operations, particularly through the function of the glycocalyx. The glycocalyx, composed of glycoproteins and glycolipids on the cell surface, serves as a crucial interface between cells and their environment, mediating important cellular processes such as cell adhesion, signaling, and recognition.^376^ In human cells, there is a complex and dynamic layer known as the glycocalyx. This layer is composed of various glycoconjugates, including glycoproteins. The glycocalyx coats all cells and plays a significant role in interactions between cells, the extracellular matrix, and pathogens. Furthermore, there are two main forms of protein glycosylation in eukaryotic cells: N‐glycosylation (or N‐linked glycosylation) and O‐glycosylation (or O‐linked glycosylation). N‐glycosylation takes place within the ER and Golgi apparatus. The Golgi apparatus is mainly responsible for synthesizing complex O‐glycans. On the other hand, O‐GlcNAcylation is predominantly observed in the cytoplasm, nucleus, and mitochondria. The process of glycosylation heavily depends on two crucial enzymes: glucosidases and glycosyltransferases (GTs) (Figure 7).^377^ Brain glycoproteins are proteins with a high degree of glycosylation, and they play a crucial role in cell differentiation and development within the CNS. These glycoproteins are involved in various processes, including synaptic connectivity, neuron differentiation, and neuron–astrocyte interactions. In addition, brain glycoproteins contain cell adhesion molecules from the plasma membrane, such as the neural cell adhesion molecule or the L1 cell adhesion molecule.^378^ Furthermore, many of the transporters and receptors found in the plasma membrane of brain cells are highly glycosylated. Examples include the N‐methyl‐d‐aspartate (NMDA) receptor (NMDAR) and the excitatory amino acid transporters 1 (EAAT1) and 2 (EAAT2), which are involved in glutamate transport. The glycan structures attached to these proteins play a vital role in regulating their function and activity.^379^

Recent studies utilizing glycoproteomic approaches have provided valuable insights into the significant role of glycosylation in NDDs.^380^, ^381^ Alterations in glycosylation have been associated with different NDDs, such as AD and PD.^376^ Furthermore, O‐glycosylation has been found to play a crucial role in influencing protein structure. Specifically, mucin‐type O‐glycosylation, a subtype of O‐glycosylation, has been identified as having a significant effect on these diseases. Additionally, multiple polypeptide N‐acetylgalactosaminyltransferases (GALNTs) have been observed to transfer N‐acetylgalactosamine to APP. Studies have shown that samples obtained from the brains of AD patients exhibit significantly higher levels of GALNT mRNA expression, particularly GALNT4, GALNT6, and GALNT10. This suggests a potential correlation between the progression of AD and mucin‐type O‐glycosylation.^382^ On the other hand, an additional in vitro study demonstrated that excessive O‐glycosylation of APP by GALNT6 led to a significant reduction in the generation of Aβ40, a variant of Aβ associated with AD pathology. While growing evidence suggests the involvement of glycosylation in NDDs, our understanding of its precise role in the CNS and its effect on behavioral abnormalities in brain diseases is still limited. Therefore, further research is necessary to unravel the significance of glycosylation in these conditions and to gain a more comprehensive understanding of its mechanisms and implications.^383^

### 6.1 Effects of abnormal glycosylation in AD

Recent studies have provided evidence supporting the involvement of glycosylation in the pathophysiology of AD through various pathways, which can affect protein function and lifespan. Glycosylation of specific proteins associated with AD has been reported to play a role in disease pathology. While the glycosylation of Tau APP is well documented in the context of AD, several other important proteins have also been consistently identified in various research studies. These proteins include transferrin, triggering receptor expressed on myeloid cells‐2 (TREM2), BACE1, APOE, and Reelin. Further details regarding the specific findings can be referred to in Table 4 of the respective research studies. The progress of research on abnormal protein glycosylation in AD has been summarized in several publications, providing a broad perspective on the effect of glycosylation at different sites on proteins and the potential applications of glycosylated proteins in the diagnosis and treatment of AD. The function of neurons and the occurrence of neurotoxicity are profoundly affected by Aβ, a hallmark protein in AD. The presence of thinning plaques in brain regions such as the hippocampus, amygdala, and cortex contributes to synaptic loss, damage, and cognitive impairment in AD. These pathological changes are associated with the progression of the disease and the manifestation of cognitive deficits.^384^ The glycosylation patterns of certain proteins have been observed to undergo alterations in neurons derived from samples of patients with AD. These findings indicate that these proteins might play a pivotal role in the progression of the disease. Moreover, research has revealed abnormal expression of several genes associated with glycosylation in these neurons. This includes a subset of genes that could potentially either worsen or alleviate symptoms related to tauopathy.^385^ An analysis of CSF from patients with AD has shown a notable inclination towards elevated endogenous peptide O‐glycosylation and reduced fucosylation.^386^ Additionally, distinct changes in the glycosylation patterns of certain N‐glycoproteins have been observed. Among these proteins are α−1‐antichymotrypsin, ephrin‐A3, and carnosinase CN1.^387^ Furthermore, in neurons derived from female patients with AD, researchers have noted an increased presence of fucosylated and bisecting GlcNAc structures. Additionally, recent studies have uncovered altered glycosylation patterns in low‐abundance proteins such as α‐1‐acid glycoprotein and C1‐inhibitor within AD neurons.^388^ Indeed, research suggests that protein N‐glycosylation is associated with various dysregulated pathways and processes in the AD brain. These include disrupted cell adhesion, neuroinflammation, dysfunction in the extracellular matrix, dysregulation of endocytic trafficking, ER dysfunction, and aberrant cell signaling. The alterations in protein N‐glycosylation have been implicated in contributing to the pathogenesis and progression of AD by affecting these critical cellular mechanisms.^389^ As previously mentioned, extensive research consistently demonstrates impaired glycosylation of crucial proteins in AD, notably Tau and APP. Both Tau and APP possess potential sites for O‐glycosylation and N‐glycosylation. The modulation of APP's glycosylation profile plays a crucial role in influencing the array of secretases involved in its processing and hydrolysis. Additionally, glycosylation has an impact on β‐secretase activity and γ‐secretase function, further highlighting the intricate influence of glycosylation on AD‐related processes. Indeed, O‐glycosylation has been demonstrated to effectively reduce the secretion of Aβ peptides. Additionally, elevated levels of Tyr10‐glycosylated Aβ peptides have been identified in CSF samples from individuals with AD, suggesting a potential impact of sialylated O‐glycans on APP processing. Furthermore, N‐glycosylation plays a role in several molecules associated with Aβ, including BACE1, APP, Nicastrin, ADAM10, Neprilysin, and TREM2. Disruptions in the N‐glycosylation of these proteins can affect their folding, maturation, and downstream pathways, potentially contributing to the development of AD. However, the exact mechanisms underlying the effects of abnormal glycosylation on these proteins and how they influence AD pathogenesis are not yet fully understood. Further research is required to elucidate these mechanisms and gain a more comprehensive understanding of the role of glycosylation in AD.^376^

**TABLE 4:** A list of proteins with abnormal glycosylated in Alzheimer disease.

Name of the protein	The protein normal function	Types of glycosylation	The impact of a protein abnormal glycosylation in AD	References
Tf	Tf is a glycoprotein that is known as an iron transport mediator	N‐glycosylation	Changes in Tf glycosylation impact iron transport dynamics, with its levels altered in AD. These alterations may indirectly regulate iron homeostasis and Tf's lifespan.	^380^, ^391^
TREM2	TREM2 regulates phagocytosis and inflammation while also involving protein degradation.	N‐glycosylation	TREM2 expression level is enhanced in AD. Changes in TREM2 glycosylation likely impact its ligand binding properties. The patterns of TREM2's glycosylation can influence the cell signaling functions of TREM2. TREM2 is an important immune receptor expressed on microglia and other myeloid cells in the brain. Alterations in TREM2 glycosylation likely lead to the disruption of key immune functions mediated by TREM2. This could trigger excessive brain inflammation, which is a hallmark of PD pathology.	^380^, ^391^
BACE1	BACE1 is known as a key enzyme involved in the production of Aβ	N‐glycosylation	BACE1 glycosylation, particularly with bisecting GlcNAc, significantly contributes to AD pathogenesis by increasing Aβ peptide production. Moreover, during oxidative stress conditions, elevated levels of bisecting GlcNAc on BACE1 can stabilize it, which is detected as a hallmark of AD.	^380^, ^391^
APOE	APOE serves diverse functions, including facilitating lipid transport within the brain and acting as a ligand for TREM2.	O‐glycosylation	Changes in the glycosylation pattern of APO might be directly associated with an elevation in the level of CSF Aβ_42_.	^380^, ^391^
Reelin	Reelin, known as a glycoprotein in the extracellular matrix, participates in maintaining synaptic plasticity and can reverse Aβ‐induced synaptic impairment.	None	The patterns of Reelin's glycosylation influence cell signaling.	^380^, ^391^

Research indicates a potential correlation between alterations in brain plasticity and the differential engagement of O‐GTs and sialyltransferases. A study examining samples from both normal and AD brain tissues has revealed significant changes in the O‐glycosylation patterns of proteins associated with neuronal plasticity in the AD samples. These findings suggest that the abnormal incorporation of O‐acetyl‐sialic acid onto O‐glycosidically linked proteins might contribute to the neuritic sprouting observed in AD samples. This altered sialylation pattern not only has the potential to initiate the degeneration of early neurofibrillary structures but also to reinforce the rigidity of glycosylated components. Consequently, it may impact the function of Tau protein and interfere with microtubule depolymerization processes. These findings shed light on the potential role of abnormal glycosylation in the pathogenesis of AD and its impact on neuronal processes related to plasticity and Tau pathology.^390^ The diverse patterns of aberrant glycosylation observed in the Tau protein could potentially serve as one of the mechanisms triggering the pathological features of AD. Notably, abnormal N‐glycosylation in the Tau protein has been detected during the early stages of AD. Subsequently, studies have demonstrated that this N‐glycosylation may contribute to later aberrant phosphorylation of the Tau protein. Furthermore, additional research has shown that the levels of phosphorylation in Tau with mutations in the N‐glycosylation region (N167Q, N359Q, and N410Q) are regulated in a site‐dependent manner. This indicates that early N‐glycosylation exerts a positive regulatory influence on Tau phosphorylation.^390^ Furthermore, the Tau protein has exhibited unexpected N‐glycosylation in AD at the N410 site on the Tau 2N4R isoform. This abnormal N‐glycosylation occurs before the abnormal phosphorylation of Tau and has been proposed as an early biomarker for AD and a marker of disease progression. In a specific N‐glycoproteomics analysis focusing on AD, researchers reported 137 proteins with differential N‐glycosylation (hyperglycosylated and hypoglycosylated) and identified 178 abnormal N‐glycosylation sites. Varied levels of glycan expression were observed in the cortex and hippocampus of AD‐affected brains compared with normal brains. Additionally, distinct glycan profiles were detected in the CSF of AD patients compared with healthy individuals. These findings suggest that alterations in N‐glycosylation have potential implications for understanding and diagnosing AD. The identification of abnormal N‐glycosylation patterns and associated proteins may provide valuable insights into the underlying mechanisms of the disease and offer potential targets for diagnostic and therapeutic approaches. However, further research is necessary to fully comprehend the significance and functional consequences of these N‐glycosylation alterations in AD.^376^

### 6.2 Effects of abnormal glycosylation in PD

Since the majority of PD cases are sporadic, a multitude of environmental factors probably play a role in the onset and progression of the disease. Epidemiological studies have established associations between PD incidence and exposure to pesticides, herbicides, and heavy metals. Moreover, metabolism and dietary factors may contribute to an individual's risk of developing PD.^392^ Therefore, a primary effect of diabetes is an imbalance in glucose metabolism, leading to hyperglycemia and other metabolic abnormalities that can potentially trigger or exacerbate the progression of PD.^393^ In fact, studies have demonstrated that individuals with diabetes who develop PD at an earlier age are more likely to experience more severe motor and nonmotor symptoms, as well as a faster progression of the disease.^393^ Consequently, impaired glucose tolerance and hyperglycemia have been identified in the majority of PD cases.^394^ The Maillard reaction can be utilized by glucose and its byproducts to react with amino groups, resulting in the formation of advanced glycation end products (AGEs), which have a detrimental impact on the function of target proteins.^395^ Proteins can accumulate and aggregate due to the cross‐linking between AGE‐modified peptides during the time‐dependent and irreversible process known as AGEs production. It is noteworthy that oxidative stress and inflammation induced by mitochondria are associated with AGE‐modified cell proteins and abnormally glycated mitochondrial proteins. Furthermore, oxidative stress itself seems to exacerbate AGE formation, creating a positive feedback loop of oxidative damage in the brain. Overall, these pathways dependent on AGEs might contribute to the neurodegenerative processes observed in PD.^396^, ^398^

### 6.3 Effects of abnormal glycosylation in HD

In the context of HD, which is primarily inherited dominantly, the key question is not whether increased production of AGEs and disrupted glucose metabolism contribute to the likelihood of HD onset. Instead, the focus is on how factors influencing glycation, such as changes in carbohydrate metabolism and heightened AGE production, may influence the age of onset and progression of HD. The exact role of AGE production in HD pathogenesis is still unknown, despite significant progress in understanding the roles of AGEs in neurodegenerative disorders. While some studies suggest that altered glucose metabolism may be an important characteristic of HD, others have found no correlation. Interestingly, epidemiological studies have reported a higher incidence of diabetes in HD patients.^397^ A proteomic investigation revealed differential expression of three glycolysis‐related proteins between HD and control brains.^399^ HD patients often rely on conventional medication treatments to manage some of their symptoms, despite the absence of available disease‐modifying medications for condition.^400^ The relationship between HD and alterations in glucose metabolism has been explored as a potential target for various strategies, including the use of different hypoglycemic medications.^401^ Previous investigations in both human subjects and animal models have examined substances such as Exendin‐4, resveratrol, glibenclamide, rosiglitazone, insulin, and the combination of glucagon‐like peptide 1 and a nonglycosylated human transferrin form (GLP‐1Tf).^402^ Administration of glibenclamide, exendin‐4, GLP‐1Tf, and resveratrol resulted in decreased blood glucose levels in animal models. Interestingly, mice responded to glibenclamide, which stimulates insulin exocytosis, but not to rosiglitazone, which enhances insulin sensitivity. This observation supports the hypothesis that, instead of insulin insensitivity, the development of diabetes mellitus in the HD mouse model could be attributed to a malfunction in insulin release. Exendin‐4 was the only medication that demonstrated the ability to improve insulin sensitivity.^403^

## 7 EFFECTS OF ABNORMAL PALMITOYLATION AND NDDs

**FIGURE 8:**
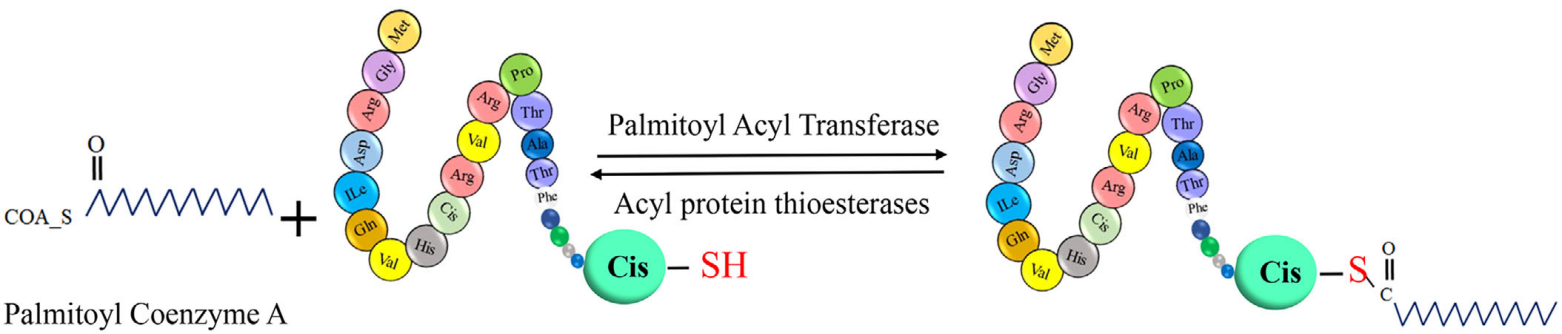
Palmitoylation is the covalent attachment of fatty acids, like palmitic acid, to the cysteine residues of proteins. Palmitoyl‐CoA is attached to the target protein by palmitoyltransferases (PATs) and removed via acyl protein thioesterases (APTs).

Palmitoylation was first reported in 1979. Since then, extensive research has identified over 3,500 palmitoylated proteins, with more than 9000 specific palmitoylation sites. S‐palmitoylation is a type of PTMs that involves the reversible attachment of a palmitate molecule to a cysteine residue of a protein via a thioester bond. The process of palmitoylation is regulated by enzymes, including palmitoyl acyltransferases (PATs), which add the palmitate. Indeed, PATs typically employ a two‐step catalytic mechanism known as the “ping‐pong” mechanism to palmitoylate their substrates. This mechanism involves the transfer of palmitoyl groups from a donor molecule, typically palmitoyl‐CoA, to the target protein.^404^ The removal of palmitate from proteins is facilitated by several classes of serine hydrolases, including acyl protein thioesterases (APTs), protein palmitoyl thioesterases (PPTs), and α/β‐hydrolase domain (ABHD) proteins (Figure 8). These enzymes play a crucial role in the dynamic regulation of protein palmitoylation by catalyzing the hydrolysis of thioester bonds between palmitate and cysteine residues in proteins. APTs, PPTs, and ABHD proteins contribute to the reversible nature of protein palmitoylation and help maintain the balance and homeostasis of this modification in cells.^405^ While 23 PATs have been identified in mammals, the understanding of depalmitoylating enzymes remains limited.^405^, ^406^ Conversely, both O‐palmitoylation and N‐palmitoylation appear to be irreversible. PATs typically feature conserved DHHC (aspartate–histidine–histidine–cysteine) tetrapeptides and zinc finger structural domains. These characteristics classify them as members of the zDHHC protein family. Each member of the zDHHC protein family exhibits distinct self‐acylation abilities and varying levels of catalytic efficiency. This variability arises from differences in their amino acid sequences, especially within the zDHHC motif and surrounding regions. In recent years, researchers have investigated the association between zDHHC family proteins and neurological disorders such as intellectual disability, HD, and schizophrenia (SCZ). Studies have utilized cell culture systems, animal models, and zDHHC‐deficient mice to explore these relationships.^404^

Protein S‐palmitoylation plays a crucial role in regulating the trafficking, stability, and enzymatic activity of proteins. Dysregulation of this lipid modification has been implicated in various human diseases, including cancer, metabolic syndrome, and infections. In the context of the nervous system, alterations in S‐palmitoylation have been linked to conditions such as AD, PD, and HD.^405^, ^407^ At the synapse, different proteins and receptors are localized to the postsynapse and presynapse regions. Recent studies compiling data from 15 palmitoyl‐proteome studies have found that palmitoylation is enriched in the synaptic proteome. It is now estimated that nearly 50% of synaptic proteins undergo palmitoylation. The reversible nature of palmitoylation enables neurons to dynamically establish and maintain specific protein localization at the synapse. This rapid reversibility of palmitoylation and its crucial role in regulating protein localization make it an intriguing mechanism for functional regulation in NDDs. The rapid reversibility of palmitoylation and its pivotal role in governing protein localization make it an intriguing mechanism for functional regulation in NDDs. Protein palmitoylation plays a critical role in regulating the differentiation of neural progenitors into neurons and facilitates axonal and dendritic growth, which are essential processes for proper neuronal development. In mature neurons, palmitoylation dynamically modulates protein localization and facilitates the movement of proteins between the synaptic membrane and intracellular compartments, including the Golgi body and ER, particularly during synaptic plasticity. Given that zDHHCs serve as key enzymes responsible for palmitoylation, their distribution within neurons is closely associated with their functional role and can profoundly impact synaptic function.^404^, ^405^ Numerous neural signaling proteins, ion channels, cell adhesion molecules, soluble N‐ethylmaleimide‐sensitive factor activating protein receptor proteins, vesicular trafficking proteins, and G‐protein receptors have been identified as being palmitoylated. Furthermore, this lipid modification has been closely associated with neuronal development and synaptic plasticity. Overall, S‐palmitoylation serves as a crucial regulatory mechanism that affects protein function and cellular processes in both normal physiological conditions and disease states. Understanding the intricate role of S‐palmitoylation in various biological systems can provide valuable insights into the pathogenesis of neurological disorders and offer potential therapeutic opportunities.

### 7.1 Roles and contributions of specific PATs in AD

A recent study has provided evidence suggesting the involvement of palmitoylation in the pathogenesis of AD. The study demonstrated that APP is palmitoylated both in vitro and in vivo, and this PTM plays a role in regulating the amyloidogenic process. APP is palmitoylated at cysteine residues C186 and C187 in its N‐terminal region. This palmitoylation regulates the trafficking of APP to the cell membrane. Mutants of APP that are deficient in palmitoylation are retained in the ER. The palmitoylation of APP is mediated by PATs zDHHC7 and zDHHC21. Overexpression of these PATs leads to increased production of Aβ and enhanced palmitoylation of APP. Recent studies have implicated zDHHC12 in regulating the palmitoylation of key proteins involved in AD. Initial findings demonstrated that zDHHC12 inhibits APP metabolism and reduces the production of Aβ in mouse‐derived neuroblastoma cells (N2A cells). This suggests a potential role for zDHHC12 in modulating the development of Aβ plaques, a hallmark of AD. Furthermore, a genome‐wide association study revealed a significant association between zDHHC12 and structural brain connectivity in a comparison of control individuals, those with mild cognitive impairment, and AD patients. These findings suggest that zDHHC12 may play a role in the alterations in brain segregation and integration observed in AD. However, further research is necessary to fully understand the underlying mechanisms and functional implications of zDHHC12‐mediated palmitoylation in AD.^404^, ^405^ In addition to the formation of amyloid plaques through the aggregation of Aβ, another neuropathological characteristic of AD involves the development of intracellular NFts comprising hyperphosphorylated Tau protein. The hyperphosphorylation of Tau is mediated by Fyn kinase. zDHHC21 serves as the PAT for Fyn. Recent studies investigating FAD using exome sequencing have identified a gene variant called zDHHC21 p.T209S. Mutations in zDHHC21 significantly enhance Fyn palmitoylation, leading to the excessive activation of GluN2B‐containing NMDARs, which, in turn, results in synaptic dysfunction and neuronal loss.^404^

Additionally, another study reports that the neuron‐specific membrane‐associated enzyme BACE1, which is involved in APP cleavage and Aβ production, may undergo palmitoylation at several cysteine residues (C478, C474, C482, and C485). Different PATs (DHHC3, DHHC4, DHHC7, DHHC15, and DHHC20) are responsible for the palmitoylation of BACE1. Aberrant palmitoylation of BACE1 may promote its localization within lipid rafts and increase Aβ production. Interestingly, palmitoylation‐deficient mutants of BACE1 did not affect APP metabolism, suggesting that BACE1 palmitoylation is not directly involved in Aβ production. It is noteworthy that drugs inhibiting PAT activity have been shown to decrease Aβ production, supporting the idea that the palmitoylation of enzymes involved in Aβ production may be necessary for Aβ accumulation. However, the role of protein palmitoylation in the onset and progression of AD is still not fully understood. Moreover, aberrant palmitoylation might also impact neurodegeneration by affecting proteins other than APP, BACE1, and the canonical Aβ synthesis pathway. Further research is needed to fully elucidate the role of protein palmitoylation in AD and its potential implications for neurodegeneration.^405^, ^407^

Nicastrin and APH‐1 are lipid raft‐associating subunits of the γ‐secretase complex and have been found to undergo palmitoylation. Nicastrin is palmitoylated at the transmembrane C689 residue, while APH‐1 is palmitoylated at the cytosolic C182 and C245 sites. Although mutations that impair the palmitoylation of these proteins do not affect γ‐secretase assembly and function, studies have demonstrated that palmitoylation is crucial for their nascent stability and association with lipid rafts. This suggests that palmitoylation plays a role in protein stability and lipid raft localization. In transgenic mouse models of AD, expressing palmitoylation‐deficient forms of APH1aL (an isoform of APH‐1) and Nicastrin resulted in significantly reduced deposition of Aβ aggregates in the frontal cortex compared with mice expressing wild‐type forms of these subunits. This indicates that the palmitoylation status of these proteins can influence the pathology of AD. These findings highlight the importance of palmitoylation in regulating the stability and localization of Nicastrin and APH‐1 in lipid rafts, and suggest that modulating their palmitoylation status could have implications for the development of therapeutic interventions targeting Aβ aggregation and AD pathology. However, further research is needed to fully understand the mechanisms underlying the effects of palmitoylation on these proteins and to explore their potential as therapeutic targets.^405^

### 7.2 Roles and contributions of specific PATs in PD

Juan et al. aimed to understand the relationship between PD and palmitoylation, specifically exploring the role of protein palmitoylation in PD and its associated pathways. The researchers analyzed the palmitoyl proteome (referred to as the palmitome) in the cerebral cortex of PD patients, comparing it with a control group consisting of four individuals. The analysis revealed the presence of 150 proteins with altered palmitoylation in PD patients compared with controls. The study provided valuable insights into the biological pathways and potential targets that could be influenced by these palmitoylation changes. Furthermore, the researchers examined the overlap between the differential palmitome identified in their study and the protein interactomes of PD‐associated proteins, such as α‐syn, LRRK2, DJ‐1, PINK1, GBA, and UCHL1. Overall, the study shed light on the altered palmitome in the cortex of PD patients, suggesting potential impacts on the cytoskeleton, mitochondrial function, fibrinogen, and cell survival. These findings indicate a potential role for protein palmitoylation in the pathophysiology of PD. The study underscores the significance of comprehensive palmitoyl‐proteomics as a valuable approach for uncovering novel cellular pathways involved in this NDD.^396^

### 7.3 Roles and contributions of specific PATs in HD

Indeed, the Htt protein can undergo palmitoylation at cysteine 214 (C214) by two PATs, zDHHC17 (also known as HIP14) and zDHHC13. However, in the context of HD, the interaction between Htt and its PATs becomes impaired due to the presence of the mHtt protein. The impaired interaction between mHtt and zDHHC17 leads to a significant reduction in the palmitoylation of mHtt compared with the wild‐type form, which is linked to increased insoluble mHtt and decreased zDHHC17 activity. In addition, studies have shown that Htt can modulate the activity of zDHHC17.^405^ However, in the presence of the HD mutation, the activity of zDHHC17 is compromised, resulting in decreased palmitoylation of its substrates. This reduction in palmitoylation affects various proteins that are targeted by zDHHC17, ultimately leading to neuronal toxicity. These findings suggest that the altered interactions between PATs, such as zDHHC17 and zDHHC13, and mutant Htt result in reduced palmitoylation of Htt and other PAT substrates. Consequently, the mislocalization of Htt and other palmitoylated proteins occurs, contributing to the pathogenesis of HD.^407^ Increasing brain palmitoylation in mouse models of HD has shown promise in rescuing the disease phenotype and is considered a potential therapeutic approach. Strategies, such as modulating PAT activity and using small molecules to enhance palmitoylation, have been explored. These approaches aim to restore palmitoylated proteins’ proper localization and function, including Htt, which is central to HD development. While the therapeutic potential is encouraging, further research is needed to optimize the approach, evaluate long‐term effects, and ensure safety and efficacy in human trials. Nevertheless, these findings lay the groundwork for developing new therapeutic strategies targeting protein palmitoylation in HD and other NDDs.^405^ Additionally, recent studies have identified SPRED1 and SPRED3 as novel substrates of zDHHC17‐mediated protein palmitoylation. These findings suggest that these proteins may play a significant role in the pathogenesis of HD associated with alterations in zDHHC17‐regulated palmitoylation. The precise relationship between palmitoylation and HD is not yet fully understood. However, current hypotheses propose that dysregulated palmitoylation of GluN2B, facilitated by zDHHC13, may contribute to the apoptosis of medium spiny neurons, ultimately leading to the development of HD.^404^ Targeting the dysregulated palmitoylation of Htt, the protein implicated in HD, has gained attention as a potential clinical treatment for the disease. It has been observed that reducing mHtt levels in the brains of HD mice does not restore abnormal palmitoylation. However, in HD patient cells, it has been demonstrated that the palmitoylation of mHtt can be normalized using APT. Promoting palmitoylation has also shown the ability to reduce mHtt aggregation and cytotoxicity in laboratory experiments. The restoration of normal palmitoylation levels through the use of an APT1 inhibitor called ML348 has shown promising results in alleviating neuropathology, movement disorders, and anxiety‐depressive behaviors associated with HD. This highlights the importance of modulating palmitoylation as a therapeutic target for HD, with a particular focus on Htt palmitoylation. Furthermore, another potential avenue for therapeutic intervention in HD involves the S‐palmitoylation of TRPC5 channels. Modulating the palmitoylation of these channels may have an impact on their expression and activity, providing novel insights into therapeutic strategies for HD.^404^

## 8 EFFECTS OF ABNORMAL METHYLATION AND NDDs

**FIGURE 9:**
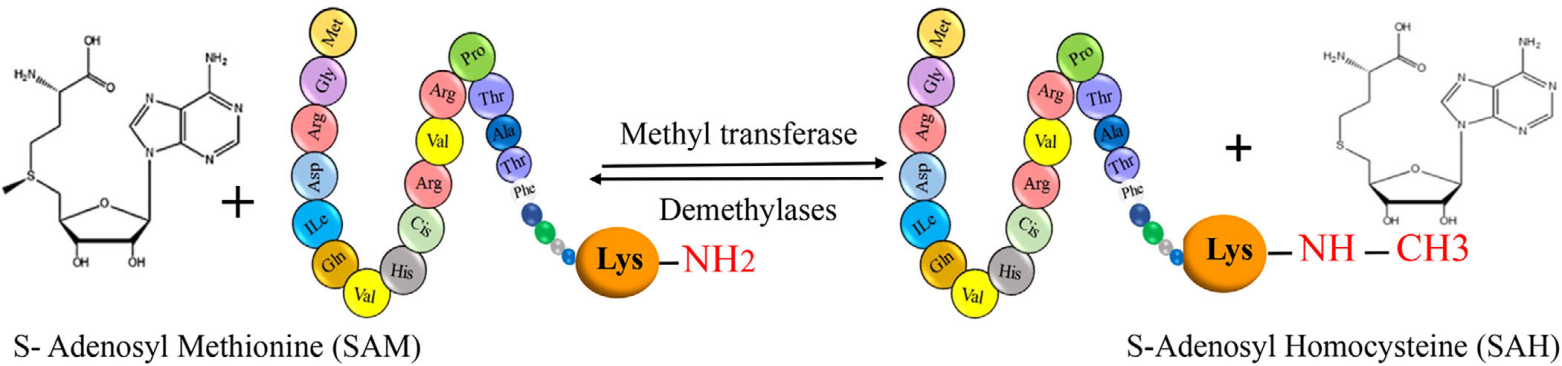
Methylation, as a reversible modification, transfers the methyl group from the donor S‐adenosylmethionine (SAM) to target residues via methyltransferases, eliminating the methyl group by the demethylase enzymes.

Methylation is a crucial epigenetic regulatory mechanism that plays diverse roles in controlling various cellular functions, including gene regulation, signal transduction, and cellular signaling pathways.^3^ This modification involves the transfer of methyl groups from S‐adenosyl methionine by methyltransferases to substrates such as DNA, RNA, and proteins, particularly to arginine and Lys residues (see Figure 9).^408^ Additionally, protein methylation plays a significant role in modulating cellular signaling pathways, thereby influencing the function of regulatory proteins involved in various signaling cascades like MAPK, WNT, BMP, and Hippo pathways. This dynamic modification can regulate PPIs, enzymatic activities, and subcellular localization, ultimately impacting the downstream effects of signaling pathways. Overall, methylation is a versatile and essential mechanism contributing to the intricate regulation of cellular processes and signaling networks. Its impact on DNA, RNA, and protein substrates, along with its involvement in cellular signaling, highlights its significance in coordinating various aspects of cellular function.^408^ Methylation occurs not only on DNA, RNA, and histones, but also on nonhistone proteins, impacting chromatin structure and gene expression. Specifically, histone methylation involves the addition of methyl groups to histone proteins by enzymes called histone methyltransferases. The consequences of histone methylation can vary depending on the specific amino acid being methylated and can either activate or repress transcription. This process plays a vital role in regulating gene expression and influences various cellular processes such as DNA repair, replication, growth, and proliferation.^6^.DNA methylation is indeed pivotal for the proper development and functioning of the human brain. It plays a significant role in a multitude of processes, including the proliferation and differentiation of neural stem cells, synaptic plasticity, neuronal repair, as well as learning and memory. While DNA itself remains physically stable, DNA methylation acts as a crucial epigenetic modification, distinct from protein methylation.^409^ Abnormal DNA methylation patterns have indeed been linked to NDDs. However, the primary focus of this review article is to delve into the impact of abnormal protein methylation in NDDs. Previous studies have suggested that abnormal methylation of the Tau protein may play a role in the development of AD.^410^

Methylation is one of the important PTMs observed in the Tau protein. This modification specifically entails the addition of methyl groups to Lys or arginine residues, leading to either mono‐methylation or di‐methylation. The enzymatic catalysts responsible for this process are Lys methyltransferases and arginine methyltransferases, specialized in targeting Lys and arginine residues, respectively, on the protein. The findings indicate that Tau, both in its physiological and pathological states, is susceptible to methylation. This intriguing observation suggests that methylation of Tau may hold functional implications not only in regular physiological processes but also in the development and progression of neurodegenerative disorders characterized by Tau pathology. Consequently, comprehensive investigations regarding Tau methylation in both normal and pathological contexts assume paramount importance in unraveling the intricate role this modification plays in NDDs.^411^ Indeed, within methylation, Tau can undergo mono‐methylation or di‐methylation on its Lys or arginine residues. These modifications are catalyzed by Lys methyltransferases or arginine methyltransferases, respectively. It has been reported that Tau can undergo methylation in both its normal and pathological states. This suggests that methylation of Tau may have functional implications in normal physiological processes as well as in the development and progression of neurodegenerative disorders where Tau pathology is observed. Studying Tau methylation in both normal and pathological contexts is crucial for understanding its role in NDDs.^412^ Indeed, specific mono‐methylation sites of Tau that are associated with PHFs have been identified in AD brain tissues. These methylation sites are primarily located in the microtubule‐binding and projection regions of Tau. Notably, the Lys‐254 site on PHFs exhibits mono‐methylation in both methylated and ubiquitylated forms. It is important to highlight that the level of methylation is higher than that of ubiquitination at this site. This imbalance in PTMs can impede the function of the UPS, leading to impaired clearance of Tau aggregation.^413^ That recent study utilizing Tau methyl mimetics sounds intriguing. It focused on investigating the effects of five Lys methylation sites, namely Lys‐174, Lys‐180, Lys‐254, Lys‐267, and Lys‐369, on the interaction between Tau and microtubules, as well as Tau aggregation. These methylation sites are distributed throughout different regions of Tau, including the proline‐rich region and the microtubule‐binding region. The findings from this study shed light on the fact that methylation at various sites within Tau can have an impact on its interaction with microtubules. This suggests that methylation plays a role in regulating Tau's functional properties and its aggregation propensity. It further emphasizes the importance of understanding the influence of different methylation sites on Tau's behavior and its implications for NDDs.^411^, ^413^ Moreover, it was observed that methylation sites within the same region do not necessarily exert the same impact on microtubule interactions. Within the MTBR, both Lys267 and Lys364, as well as the nearby Lys‐174 within the proline‐rich region, have been found to decrease the interaction between Tau and microtubules. These modifications contribute to the development of Tau pathology. On the other hand, Lys‐254 and Lys‐180, which are also located in these regions, do not seem to impact microtubule‐binding. Indeed, the regulation of microtubule‐binding in Tau is dependent on specific methylated sites. Furthermore, it has been observed that individual Tau methylmimetics alone are insufficient to trigger Tau aggregation. However, in the presence of the P301L Tau mutation, they promote Tau aggregation. Hence, the combination of these single methylated sites and the P301L Tau mutation contributes to the overall effects on Tau–microtubule interactions and subsequent aggregation. It suggests that these single methylated sites are not the initiator of Tau misfolding but contributes to Tau aggregation after its initiation.^411^, ^413^ Furthermore, an additional study has revealed that Lys methylation, similar to phosphorylation, is a prevalent PTM on Tau. This study has even uncovered novel Lys mono‐methylation sites, including Lys‐130, Lys‐150, Lys‐294, Lys‐298, Lys‐343, and Lys‐438, within soluble Tau from human brains. To investigate these mono‐methylated sites, researchers utilized site‐specific meK‐Tau antibodies, which allowed for the recognition and detection of these modifications. This study has provided valuable insights into the diverse landscape of Tau modifications and their potential relevance in Tau‐related pathologies. Notably, it has been discovered that the accumulation of meK130 (methylated Lys‐130) and meK132 (methylated Lys‐132)‐modified Tau increases with Braak stage in human brains, specifically in the soluble fraction rather than the insoluble aggregates. Additionally, the study identified histone‐Lys N‐methyltransferase (SETD7) as a Lys methyltransferase responsible for methylating Tau at Lys‐132, which seems to facilitate the methylation of Lys‐130. Interestingly, these two modifications exhibit an age‐related increase in the soluble fraction in a mouse model of tauopathy. These findings shed light on the dynamic nature of Tau methylation and its potential involvement in the progression of Tau‐related disorders across different stages of disease and aging.^414^ Additionally, studies Tau methylation at Lys sites have been detected that mono‐methylation at Lys residues is not only observed in soluble Tau extracted from healthy human brains but also in samples from individuals with AD. The similar levels of Lys methylation in both conditions suggest that Lys methylation is a physiological modification of Tau. However, it has been observed that this modification changes from di‐methylation to mono‐methylation during aging and in the context of AD. This implies that Lys mono‐methylation of Tau may play a role in Tau pathology, and increasing Tau methylation levels could potentially act as a contributing factor in reducing Tau aggregation. Furthermore, it has been documented that Lys methylation of Tau is a physiological modification that exerts a neuroprotective role by reducing Tau's propensity for aggregation and promoting tubulin assembly.^415^, ^416^ The recent study reveals that mono‐methylation of Tau at Lys‐317 reduces Tau solubility and promotes the formation of Tau assemblies. Interestingly, the effect of Lys‐317 methylation on Tau solubility appears to be independent of phosphorylation. Additionally, the study highlights that methylation of Lys‐317 in Tau can prevent the Tau‐dependent proliferation of Hela cells. These findings shed light on the complex nature of Tau methylation and its diverse effects on Tau solubility and cellular processes. Further research is necessary to fully understand the mechanisms underlying these observations and their implications in the context of NDDs.^417^

## 9 EFFECTS OF ABNORMAL ADPR OF PROTEINS IN NDDs

**FIGURE 10:**
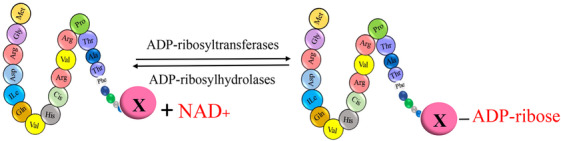
ADP‐ribosylation involves the covalent attachment of one or more ADP‐ribose moieties from NAD^+^ to specific residues on target proteins, such as cysteine, arginine, glutamic acid, aspartic acid, and serine (x). This modification is catalyzed by a family of enzymes called ADP‐ribosyltransferases (ARTs). The ADP‐ribosylation of proteins is a reversible process, as specialized enzymes called ADP‐ribosylhydrolases can remove the ADP‐ribose moieties.

ADPR was discovered over 60 years ago. Since then, ADPR has emerged as a crucial PTM due to its involvement in diverse cellular processes and reversible nature. This modification plays a crucial role in various essential processes, including DNA damage repair, transcriptional regulation, cell growth, differentiation, stress responses, and apoptosis.^418^ Multiple studies have provided evidence that ADPR modification occurs on specific amino acid residues, including arginine, serine, Lys, glutamate, cysteine, and aspartate. This process is facilitated by a family of enzymes known as ADP‐ribosyltransferases (ARTs) or poly ADP‐ribose polymerases (PARPs) (Figure 10). This group of enzymes consists of distinct members, each characterized by unique structures and functions. These enzymes covalently attach ADP‐ribose units to specific residues on substrate proteins.^418^ ARTs play a crucial role in catalyzing the mono ADPR of proteins, which occurs either extracellularly or at the cell surface. This modification plays an instrumental role in governing intercellular communication dynamics and initiating signaling transduction pathways. Moreover, this modification can involve the attachment of one or multiple ADP‐ribose units to a protein. This process can occur as a single ADP‐ribose unit, known as mono (ADP‐ribose) or MAR, or as the assembly of multiple ADP‐ribose units, referred to as poly (ADP‐ribose) or PAR (PARylation).^419^ ARTs can catalyze the transfer of the ADP‐ribose unit from the NAD^+^ to the other unit in a specific target protein, forming glycosidic bonds between the units. This process leads to the formation of a linear chain of PAR.^419^

The ARTs superfamily consists of 17 proteins that are distinguished by their specific enzymatic functions. The PARP family members are further classified into “mono” and “poly” enzymes. Among them, 11 enzymes function as mono (ADP‐ribose) transferases. These include PARP‐3, ‐4, ‐6, ‐7, ‐8, ‐10, ‐11, ‐12, ‐14, ‐15, and ‐16, which attach a single ADP‐ribose unit to target proteins. These enzymes are referred to as monoenzymes. Conversely, PARP‐1, ‐2, ‐5a, and ‐5b have the ability to sequentially attach ADP‐ribose subunits, catalyzing the formation of ADP‐ribose chains through α (1 → 2) O‐glycosidic bonds. This process leads to the formation of poly (ADP‐ribose) chains, known as polyenzymes. It should be noted that some studies have reported a lack of discernible enzymatic activity for PARP‐9 and ‐13.^420^ Additionally, PARP‐1 is recognized as the main enzyme responsible for over 90% of cellular PARP activity, distinguishing it as a key player among the PARP family members. In contrast, PAR glycohydrolase (PARG) plays a crucial role in the removal of ADP‐ribose units from poly (ADP‐ribose) polymers. PARG's function is to enzymatically cleave the glycosidic bonds within PAR, thereby reversing the PARylation process.^421^

Emerging research suggests that the interplay between PARylation and PAR‐binding significantly influences the aggregation tendencies of proteins involved in NDDs like AD and PD.^421^ Some important NDDs‐associated proteins, such as Aβ and α‐syn, have been reported to contribute to the activation of PARP‐1 through the generation of ROS and/or reactive nitrogen species (RNS). As a result, these NDD‐associated proteins undergo PARylation, which enhances their toxic effects and exacerbates neuronal death.^422^ Therefore, excessive activation of PARP‐1 has been identified in both NDDs and aging. This hyperactivation leads to a series of detrimental effects, including neuroinflammation and mitochondrial dysfunction. Other studies have observed elevated expression levels of PARP‐1 in glial cells and neurons within brain samples from certain NDDs. Furthermore, it has been discovered that neuronal loss is attributed to PAR‐dependent neuronal death.^422^ Although apoptosis continues to be a central area of study in cellular biology, the concept of PAR‐dependent cell death, referred to as “parthanatos,” has gained recognition as a significant contributor to different forms of neuronal death. Parthanatos is a distinct mechanism of necrotic cell death that occurs in response to various stressors, such as DNA damage and oxidative stress, triggered by the hyperactivation of PARP‐1. Consequently, excessive PARP activity can lead to cell death by depleting NAD^+^ levels in response to oxidative DNA damage. This phenomenon is considered a potential mechanism underlying neuronal death in NDDs. Studies have demonstrated that the PAR polymer itself can exert direct toxicity on neurons. However, the degradation of PAR polymers by PARG serves as a protective mechanism, preventing cell death induced by PAR polymer accumulation.^423^ This process and the activation of PARP‐1 serve as crucial points in the pathogenesis of NDDs, underscoring its potential as a therapeutic target. Furthermore, neuroinflammation is a prominent characteristic of NDDs. PARP‐1 contributes to inflammation by enhancing the activity of inflammatory genes such as NF‐κB, a TF involved in immune responses, and the aminoacyl‐tRNA synthetase complex‐interacting multifunctional protein‐2 (AIMP2). These molecular interactions further highlight the involvement of PARP‐1 in the inflammatory processes associated with NDDs.^422^ Indeed, comprehending the diverse roles of PARP‐1 offers valuable insights into the intricate interplay of molecular pathways implicated in neurodegeneration. This understanding is crucial for developing innovative treatment approaches aimed at inhibiting PARP‐1 abnormal activity in the NDDs.

### 9.1 Effects of abnormal PARP‐1 hyperactivation in AD

Approximately 20 years ago, hyperactivation of PARP was detected in samples from the brains of AD patients. The study specifically investigated the levels of PARP and PAR in the frontal and temporal lobes of brain samples obtained from individuals with AD. Indeed, the findings from the study revealed a significant increase in both PARP and PAR levels in the frontal and temporal lobes of patients with AD compared with those of healthy controls. Consequently, an increased level of PARylation in nuclear proteins was detected in the brains of these samples. These observations strongly suggest that dysregulated PARylation, characterized by increased PARP activity and PAR accumulation, may play a role in the pathogenesis of AD. Moreover, it is worth mentioning that within these samples, there was a remarkable concentration of PARylation observed in neurons, especially in small pyramidal neurons. On the other hand, astrocytes displayed a relatively lesser degree of PAR accumulation, while no discernible signs of PAR accumulation were noted in microglia. This finding laid the foundation for subsequent investigations aimed at understanding the specific cellular and molecular mechanisms underlying PARP‐mediated pathology in various NDDs.^421^ Furthermore, studies have provided evidence of an association between the activation of PARP‐1 and AD, with increased PARP‐1 activity observed in samples from AD patients. The initiation of PARP‐1 activation in response to oxidative stress is thought to be a critical factor in the pathogenesis of AD. Furthermore, the oligomerization of Aβ triggers a series of events that lead to the generation of free radicals and subsequent oxidative stress in the disease. In particular, it has been reported that the presence of Aβ peptides can promote PARP‐1 activity. Consequently, the activation of PARP‐1 by Aβ can result in the release of apoptosis‐inducing factor, an oxidoreductase found in mitochondria, from the mitochondria into the cytoplasm. This process ultimately contributes to the induction of cell death. Microglia cells also have diverse functions in AD, including neuroinflammation and neurodegeneration. In response to the accumulation of Aβ plaques, microglia become activated and release proinflammatory cytokines. As a result, this chronic neuroinflammatory condition is a characteristic feature of AD. Research findings highlight the significance of PARP‐1 in microglial activation induced by Aβ, and suggest that these effects may be related to the interactions between PARP‐1 and NF‐κB. Therefore, suppressing PARP‐1 activity could potentially inhibit microglial activation, offering therapeutic advantages for AD by reducing neuroinflammation. Overall, these findings emphasize the multifaceted roles of PARP‐1 in AD pathogenesis, including its involvement in apoptotic pathways, neuroinflammation, and microglial activation. Targeting PARP‐1 activity may hold therapeutic potential in mitigating the detrimental effects of Aβ accumulation and neuroinflammation in AD.^424^

### 9.2 Effects of abnormal PARP‐1 hyperactivation in PD

Emerging research robustly indicates a significant link between PARP‐1 and PAR in neuronal death. Furthermore, investigations have revealed an association between α‐syn toxicity, the predominant protein implicated in PD, and PARP‐1. Extensive research has been conducted on the interaction between α‐syn and PARP. α‐syn present in extracellular vesicles and the activity of PARP‐1 have been identified as contributing factors in the progression and pathogenesis of PD. Based on in vitro experiments, it has been observed that α‐syn preformed fibrils (α‐syn PFF), derived from purified recombinant human wild‐type α‐syn, directly induce the activation of PARP. This activation is closely linked to the parthanatos pathway, recognized as a pivotal factor in neuronal death and the progression of neurodegeneration, notably within the context of PD. These studies provide substantial evidence indicating the significant role of PARP in PD and likely in other types of synucleinopathies. Parthanatos is initiated by the hyperactivation of PARP‐1 following direct and/or indirect DNA damage. Additionally, α‐syn PFF triggers the activation of neuronal NOS (nNOS), leading to NO production and potentially causing an elevation in NO levels. NO reacts with superoxide (O^2−^) inside mitochondria, resulting in the production of peroxynitrite (ONOO^−^). Therefore, α‐syn aggregation can directly impact PARP‐1 activity. The aggregation of α‐syn protein leads to a subsequent cascade, initiating the activation of NOS and inducing NOS synthesis. This process leads to DNA damage and subsequently activates PARP‐1, inducing the nuclear synthesis of poly (ADP‐ribose). In pathological states, the production of PAR sets off a series of events whereby it translocates into the cytosol and interacts with α‐syn, ultimately enhancing the formation of fibrils and the misfolding of cytotoxic α‐syn. Consequently, the accumulation of α‐syn leads to neuronal death through the parthanatos pathway. The activation of PARP‐1 occurs not only in response to direct DNA damage induced by ROS and exposure to UV radiation but also through its direct interaction with AIMP2, independently of DNA damage. As a result, the excessive activation of PARP‐1 triggers the synthesis and accumulation of poly(ADP‐ribose) polymers.^425^, ^426^ Recent investigations have revealed that the conversion of pathologic α‐syn to a more toxic form can occur through PAR. Elevated levels of PAR have been observed in the CSF and brains of individuals with PD, indicating the probable involvement of PARP activation in the development of the disease. Furthermore, α‐Syn and PARP‐1 have been detected in the plasma samples of PD patients. Overall, the activation of PARP‐1 by pathologic α‐syn leads to the acceleration of PAR production, thereby exacerbating neuronal death through parthanatos. Research suggests that monitoring the concentrations of α‐syn and PARP‐1 in patients' plasma samples could serve as valuable biomarkers, offering a noninvasive approach to diagnosing PD and probably its severity. Strategies involving the use of PARP inhibitors to suppress PARP‐1 activity have shown effectiveness in PD therapy. These findings emphasize the potential of targeting PARP‐1 activation as a therapeutic avenue to mitigate the damage to dopamine neurons in PD.^426^, ^427^

## 10 EFFECTS OF ABNORMAL S‐NITROSYLATION OF PROTEINS IN NDDs

**FIGURE 11:**
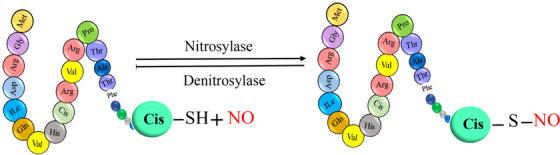
S‐nitrosylation is indeed a reversible modification involving the covalent interaction of a nitric oxide group (‐NO) with the thiol group of cysteine residues (‐S‐) on a target protein. This modification is catalyzed by nitrosylases and can be removed by denitrosylase enzymes.

S‐nitrosylation is a PTM that regulates protein function by covalently reacting NO‐related species with cysteine thiol groups on target proteins. This PTM has a significant impact on protein function as it regulates protein conformation, enzymatic activity, PPIs, and cellular localization. Similar to phosphorylation, S‐nitrosylation serves as an important modulator of signal transduction pathways under physiological conditions. It specifically modifies cysteine residues and reflects both the pathological and physiological impacts of NO on cellular processes (Figure 11). While NO is involved in normal neuronal function and survival, excessive generation of NO resulting from a toxic environment or the normal aging process can lead to neuronal and synaptic abnormalities. Abnormal S‐nitrosylation reactions can occur and affect various cellular processes, including protein misfolding, mitochondrial fragmentation, synaptic function, apoptosis, or autophagy. Proteins that experience aberrant S‐nitrosylation play a crucial role in the pathogenesis of NDDs such as AD and PD.^428^, ^429^

These observations suggest that targeting S‐nitrosylation may hold therapeutic potential for the treatment of NDDs. By modulating S‐nitrosylation, it may be possible to intervene in the disease processes associated with NDDs and mitigate their pathological effects. Such interventions could potentially lead to the development of novel treatments for NDDs.^428^, ^429^ In brain cells, the production of NO is highly dependent on a family of three NOS enzymes: nNOS or NOS1, inducible NOS (iNOS or NOS2), and endothelial NOS (eNOS or NOS3). These enzymes, whose necessary cofactors are oxygen, NADPH, and tetrahydrobiopterin, utilize l‐arginine and generate NO. In the nervous system, the activation of NMDA‐type glutamate receptors is a well‐known mechanism for NO generation. When NMDARs are stimulated by the neurotransmitter glutamate, it leads to the influx of calcium ions through receptor‐associated ion channels.^429^ This increase in calcium concentration activates nNOS, resulting in the generation of NO. In the context of AD, the presence of Aβ oligomers, which are implicated in synaptic dysfunction, leads to the induction of NO at a pathological level through the hyperstimulation of extrasynaptic NMDARs. This excessive activation of NMDARs plays a critical role in various NDDs by promoting the pathological generation of ROS and RNS. These reactive molecules can cause oxidative damage and contribute to the progression of NDDs.^429^

### 10.1 Effects of abnormal S‐nitrosylation in AD

Based on previous studies, it can be suggested that abnormal nitrosylation of the NMDAR and Drp1 protein is implicated in the development of AD. It is well established that protein S‐nitrosylation plays a crucial role in the disrupted function of the NMDA receptor. Under normal physiological conditions, the NMDA receptor is involved in the regular production of NO, which contributes to the proper functioning of neurons. However, the abnormal accumulation of proteins, such as Aβ oligomerization and α‐syn aggregation, or the activation of other stimulators, leads to the extrasynaptic activity of NMDA receptors, resulting in elevated levels of RNS. Consequently, this aberrant RNS production causes mitochondrial impairment, abnormalities in gene transcription, and protein aggregation.^430^ It has been documented that five cysteine residues of the NMDA receptor, including Cys‐744 and Cys‐798 of the GluN1 subunit, and Cys‐87, Cys‐320, and Cys399 of the GluN2A subunit, are involved in S‐nitrosylation, all of which play a neuroprotective role by reducing the receptor's activity. While Cys‐744, Cys‐798, Cys‐87, and Cys‐320 residues of NMDA receptors are prone to form disulfide bonds in highly oxidizing conditions, low oxidizing conditions, such as low brain oxygen in a physiological state, favor free thiol groups over sulfide. Additionally, Cys‐744 and Cys‐798 are considered to be particularly susceptible to S‐nitrosylation once a reduction occurs in the disulfide bond between them. This is consistent with the notion that Cys‐744 and Cys‐798 develop their S‐nitrosylation under hypoxic conditions. Furthermore, the S‐nitrosylation of Cys‐744 and Cys‐798 residues seems to facilitate the S‐nitrosylation of Cys‐399 by inducing a conformational change. Interestingly, inhibiting the S‐nitrosylation of these cysteine residues, such as through cysteine to alanine mutation, can eliminate the inhibitory impact of S‐nitrosylation on NMDA receptor function. Therefore, the S‐nitrosylation of the NMDA receptor may potentially prevent its extrasynaptic activity in pathological conditions like AD.^430^

Drp1, a GTP enzyme, plays a crucial role in mitochondrial division, which contributes to neuronal function. In a recent study, it was found that under physiological S‐nitrosocysteine (SNOC), S‐nitrosylated ubiquitin C‐terminal hydrolase isozyme‐L1 (Uch‐L1) is generated in SY5Y neurons. This finding was further supported by experiments conducted on human embryonic kidney (HEK) cells using endogenous NO, produced by neuronal NO synthase (nNOS). In addition to wild‐type mice, S‐nitrosylation of Uch‐L1 has also been identified in AD mouse models. Indeed, the S‐nitrosylation of Uch‐L1 has been observed not only in wild‐type mice but also in mouse models of AD. Furthermore, postmortem analysis of AD patient brains has revealed elevated levels of S‐nitrosylated Uch‐L1 compared with control groups. This suggests that the increased formation of S‐nitrosylated Uch‐L1 occurs under conditions of nitrosative stress and in pathological conditions like AD. Evaluation of Uch‐L1 indicates that its Cys152 residue is a potential site of S‐nitrosylation. Following S‐nitrosylation, Uch‐L1's ability to interact with ubiquitin is reduced, resulting in a subsequent decrease in its deubiquitinylation function. In fact, both the brains of patients with AD and AD mouse models have shown diminished levels of activated Uch‐L1, indicating a negative effect of S‐nitrosylation on Uch‐L1 activity.^431^ Furthermore, when primary cerebrocortical neurons were treated with Aβ oligomers, S‐nitrosylation of Uch‐L1 was observed, resulting in the loss of spine density. In vivo models of AD that overexpress Aβ oligomers have also demonstrated neuronal synapse loss, suggesting that S‐nitrosylated Uch‐L1 contributes to synapse loss in mouse models of AD. It has been found that CDK5, a substrate of S‐nitrosylation, is involved in the S‐nitrosylation of Drp1. Research reports that exposure of HEK293 cells to SNOC (a nitrosative stress inducer) stimulates S‐nitrosylation of Uch‐L1, CDK5, and Drp1. However, depletion of Uch‐L1 reduces both S‐nitrosylation of CDK5 and Drp1. These results suggest that, under SNOC treatment, Uch‐L1 may regulate the S‐nitrosylation of both CDK5 and Drp1 by transferring its NO group to CDK5 and subsequently to Drp1.^431^ Nevertheless, Uch‐L1 depletion studies have shown that the levels of SNO‐CDK5 and SNO‐Drp1 are not completely diminished, indicating that SNOC may transfer its NO nonenzymatically. This suggests the possibility of downstream transnitrosylation reactions facilitated by S‐nitrosylation of Uch‐L1. Additionally, since there is a delay in the production of SNO‐Drp1 compared with SNO‐CDK5, it is also possible that the NO group may be transferred from CDK5 to Drp1. Supporting these findings, further experiments have provided evidence that SNO‐CDK5 transnitrosylates Drp1. Consistent with these results, the research has documented a transnitrosylation pathway from SNO‐Uch‐L1 to CDK5, and subsequently to Drp1 in cells expressing HA‐CDK5 or V5‐Uch‐L1. This suggests that S‐nitrosylation establishes a transnitrosylation network among SNO‐Uch‐L1, CDK5, and Drp1, which contributes to the pathogenicity of neuronal synapse.^431^

It has been documented that the enzymes involved in the tricarboxylic acid (TCA) cycle can undergo S‐nitrosylation. In the brains of both male and female patients with AD, an increase in the levels of S‐nitrosylated TCA cycle enzymes has been observed. This includes enzymes such as aconitase, dihydrolipoyl dehydrogenase (DLD), various dehydrogenases (including pyruvate dehydrogenase), and mitochondrial malate dehydrogenase (MDH). In line with AD patients, high levels of S‐nitrosylation have also been observed in the 𝛼KGDH, DLD, and IDH 𝛼‐subunit in P1 (PSEN1 or PS1) mutant AD‐hiN compared with isogenic WT/controls. Among these enzymes, SNO‐IDH 𝛼‐subunit exhibits a particularly significant increase. Furthermore, AD‐hiN has been shown to decrease the levels of the citrate (Cit)/𝛼KG ratio. Specifically, while 𝛼KG levels are elevated, there is no significant change in the level of Cit in AD‐hiN.^432^ It is worth noting that l‐NG‐nitro arginine methyl ester (l‐NAME) is an inhibitor of NO synthesis. The data indicate that when exposed to l‐NAME, the Cit/𝛼KG ratio is reduced in WT/Control‐hiN, thereby alleviating the basal inhibition caused by S‐nitrosylation at the level of aconitase/isocitrate dehydrogenase (Aco/IDH). However, it is interesting to note that l‐NAME causes a further decrease in the Cit/α‐ketoglutarate (KG) ratio and alleviates the lower block at the level of aconitase/isocitrate dehydrogenase (Aco/IDH) in AD‐hiN compared with WT/Controls. This suggests that SNOC (S‐nitrosylating species) contributes to the reduction in the Cit/𝛼KG ratio, indicating that the elevated baseline levels of NO, along with NO donors, lead to increased S‐nitrosylation. This, subsequently, can cause a block upstream from Aco/IDH and ultimately restricts Cit production. In addition to the findings mentioned, SNOC (S‐nitrosylating species) also increases the levels of 𝛼KGDH/succinyl coenzyme‐A synthetase (SCS), which results in the suppression of 𝛼KGDH/SCS activity due to the presence of NO‐related species.^432^ This elevation in protein S‐nitrosylation leads to the inactivation of the enzyme. Furthermore, in both AD patients and controls, mitochondrial MDH undergoes S‐nitrosylation at cysteine residue 93 within the TCA cycle. Intriguingly, while there is a decrease in S‐nitrosylation of the cysteine 93 residue, S‐nitrosylation of cysteine 212 occurs in AD patients. However, the research suggests that these modifications do not have a significant impact on enzymatic activity in cell‐based models. According to the study, under conditions of mitochondrial stress, AD‐hiN exhibits a reduction in the maximal rate of respiratory capacity, while WT/isogenic‐hiN shows higher levels of glycolysis capacity. Importantly, when AD‐hiN is treated with dimethyl succinate, not only does it improve synapse density, but it also contributes to the production of NADH. This finding aligns with the idea that by circumventing the inhibitory step at the 𝛼KGDH/SCS stage, which is where NADH is generated, additional NADH is produced further downstream in the TCA cycle. For example, this can occur at the malate to oxaloacetate conversion step mediated by MDH.^432^

### 10.2 Effects of abnormal S‐nitrosylation in PD

PINK1 is a Ser/Thrprotein kinase consisting of 581 amino acids. Similar to Parkin, PINK1 plays a crucial role in regulating mitophagy and maintaining mitochondrial quality control. Mutations in both the pink1 (PARK6) and parkin (PARK2) genes are associated with the early onset of PD, which is characterized by mitochondrial impairment. PINK1 is composed of various structural domains, including a N‐terminal mitochondrial targeting motif, a transmembrane domain, an outer‐mitochondrial membrane localization signal, a highly conserved protein Ser/Thr kinase domain, and a C‐terminal autoregulatory sequence. In its normal state, endogenous PINK1 exists as a full‐length precursor protein that is constitutively produced in the cytosol. Upon generation, PINK1 is imported into the mitochondria and undergoes proteolytic cleavage, resulting in the generation of mature PINK1.^433^, ^434^

Subsequently, translocation of mature PINK1 to the cytosol leads to rapid turnover and low steady‐state concentration. However, mounting evidence suggests that PINK1 is directed to mitochondria in response to mitochondrial stress. Once in the mitochondria, PINK1 phosphorylates parkin and ubiquitin, recruiting them and resulting in the parkin‐dependent ubiquitination of mitochondrial proteins. Damaged mitochondria subsequently recruit autophagy adaptor proteins, including p62/SQSTM1, and are eventually removed by lysosomes. Thus, PINK1 acts as a mitochondrial protein kinase that is involved in mitophagy, a process critical for numerous cellular functions.^435^ Evidence also indicates that PINK1 can be a substrate for S‐nitrosylation. Exposure to the physiological NO donor SNOC reveals that PINK1 can be S‐nitrosylated in the SH‐SY5Y dopaminergic human‐derived neural cell line.^433^ Additionally, NO produced from nNOS amplifies the S‐nitrosylation of PINK1. PINK1 contains several cysteine residues, namely Cys‐92, Cys‐166, Cys‐564, and Cys568, which are located within potential S‐nitrosylation motifs.^433^, ^436^ These residues have been extensively studied to determine the primary site of S‐nitrosylation on PINK1. Interestingly, Cys568 has been identified as the critical site of S‐nitrosylation on PINK1. This cysteine residue is situated in the C‐terminus of both the full‐length and cleaved forms of PINK1. Notably, it is worth mentioning that PD mutations that impair the enzymatic activity of PINK1 are also found in the C‐terminus of the protein.

In addition to these findings, S‐nitrosylated PINK1 has also been observed in a‐Syn transgenic mouse models of PD. Importantly, the S‐nitrosylation of PINK1 has been shown to suppress its kinase activity. Treatment with SNOC leads to a decrease in the levels of auto‐phosphorylated PINK1, both in the presence and absence of the mitochondrial depolarizing agent carbonyl cyanide 3‐chlorophenylhydrazone (CCCP). CCCP is known to promote the localization of full‐length PINK1 to damaged mitochondria, thereby inducing mitophagy. Consistent with these findings, it has been observed that Parkin, being a substrate for PINK1's enzymatic activity during mitophagy, undergoes reduced phosphorylation by PINK1 when subjected to SNOC treatment. Thus, S‐nitrosylated PINK1 not only loses its auto‐phosphorylation ability but also the ability to phosphorylate other substrates. In alignment with this result, SNOC treatment reduces the levels of full‐length PINK1. Nevertheless, while the levels of cleaved PINK1 initially show a transient elevation, subsequently they show a decline. Interestingly, the research indicates that the low levels of PINK1 expression resulting from SNOC treatment are independent of SNO‐PINK1 formation. The translocation of Parkin to mitochondria contributes to the induction of mitophagy. However, S‐nitrosylation of PINK1 has a negative impact on this mechanism in neuronal cells. This highlights the notion that S‐nitrosylated Parkin impedes its phosphorylation and subsequent recruitment to damaged mitochondria.^436^, ^437^ Subsequently, in line with this concept, it has been documented that S‐nitrosylation of PINK1 has a potential role in hindering PINK1/Parkin‐mediated mitophagy. Valinomycin, an ionophore that contributes to the induction of mitophagy, has been shown to sustain the levels of full‐length PINK1 and enhance ubiquitin phosphorylation in hiPSC‐DA neurons. Valinomycin also depolarizes mitochondria, leading to increased uptake of l‐arginine, which serves as a substrate for NOS and promotes the production of NO. In the human context (specifically, hiPSC‐derived dopaminergic neurons), research indicates that valinomycin induces endogenous NO, and high levels of endogenous NO block mitophagy triggered by valinomycin. Additionally, valinomycin plays a role in the stimulation of endogenous SNO‐PINK1, which is present at a pathophysiologically relevant level. Further experiments have demonstrated that SNO‐PINK1 suppresses mitophagy induced by valinomycin through attenuating PINK1/Parkin‐dependent mitophagy. These results suggest that S‐nitrosylation of PINK1 could potentially serve as a novel therapeutic strategy not only for PD but also for other NDDs.^436^

Parkin, as an E3 ubiquitin ligase, plays a crucial role in counteracting excessive intracellular free radicals, thereby contributing to cell survival. Under conditions of nitrosative stress (such as GSNO treatment), the E3 ligase activity of parkin initially increases but subsequently decreases over time. Additional experiments have demonstrated the S‐nitrosylation of parkin in in vivo models, as well as samples from PD and those with diffuse LB disease (DLBD). In this research, the level of ubiquitinated parkin has been measured to assess the ubiquitin E3 ligase activity of parkin, as parkin can induce autoubiquitination.^433^ Furthermore, the monitoring of Parkin‐mediated ubiquitination of synphilin‐1 has been utilized as an indicator of parkin activity. Subsequent research has revealed a notable reduction in the E3 ligase activity of parkin, which can be restored by the administration of an NOS inhibitor called nitro‐l‐arginine. Additionally, S‐nitrosylated parkin loses its protective function against stress‐induced apoptosis, including toxicity induced by α‐syn and synphilin‐1, when exposed to a proteasomal inhibitor called MG132. Another study has provided evidence for the presence of S‐nitrosylated parkin in in vitro models, as well as samples from mice treated with S‐nitrosylating agents, and temporal cortex brain samples from patients with sporadic PD and DLBD. However, the research suggests that parkin activity initially increases but then declines due to auto‐ubiquitination. Furthermore, peptide mass fingerprinting and MS analysis have identified specific target residues of S‐nitrosylation, including seven cysteines within the RING1 domain and five cysteines within the IBR‐RING2 domain. However, the precise impact of S‐nitrosylation on each site remains unknown.^433^ In this context, it has been reported that S‐nitrosylation of the Cys‐323 residue of Parkin can enhance mitochondrial quality control; however, this modification is restricted under conditions of excessive NO generation. Furthermore, it has been observed that S‐nitrosylated residues of Parkin, including the Cys‐323 residue involved in zinc coordination, can lead to irreversible conformational disruption and inactivate Parkin.^438^ Investigations have also documented that in SH‐SY5Y cells transfected with Parkin, both oxidized NO and S‐nitrosylated Parkin levels are elevated following treatment with S‐nitrosoglutathione (GSNO) and MPP+. Under these conditions, divalent metal transporter 1 (DMT1), which is responsible for iron uptake, and cell death significantly increased as well. DMT1 is an iron import protein that is expressed in mammalian cells. Animal models of PD have shown an increased expression of DMT1. DMT1 serves as a substrate for ubiquitination by parkin, but S‐nitrosylation of parkin prevents its ubiquitination. Furthermore, inhibition of S‐nitrosylation of parkin reduces the levels of DMT1. Consistent with these findings, S‐nitrosylated parkin enhances iron uptake, which can lead to cellular death. The hallmark characteristic of PD is the loss of dopaminergic neurons in the SN. Excessive production of RNS resulting from nitrosative stress can be detrimental to dopaminergic neurons in the SN. Interestingly, both S‐nitrosylated parkin and DMT1 show a significant increase in the SN of PD mice, while there is no alteration in their expression in the striatum.^439^

Indeed, DJ1 is a modulator that plays a crucial role in mediating the antioxidant response and regulating mitochondrial function. Evidence suggests that the deletion of DJ1 leads to an increase in neuronal cell death. Interestingly, DJ1 also plays a significant role in the S‐nitrosylation of parkin. Depletion of DJ1 prevents the S‐nitrosylation of endogenous parkin and results in the upregulation of parkin. Both DJ1 depletion and mutations in the cysteine residues of parkin that prevent S‐nitrosylation have been shown to increase neuronal cell death, resembling the phenotypes observed in cases of mitochondrial depolarization and dysfunction. Indeed, these findings are in line with previous studies indicating that S‐nitrosylation of parkin plays a neuroprotective role by modulating its E3 ligase activity, which in turn helps maintain mitochondrial homeostasis by promoting autophagic mitochondrial degradation. Consequently, inhibiting DJ1 and thereby promoting the denitrosylation of parkin seems to play a significant role in the pathogenesis of PD. Therefore, the activation of physiological S‐nitrosylated parkin could be considered a potential novel therapeutic strategy.^438^ Indeed, further research is necessary to fully understand the precise function of S‐nitrosylated parkin. GSNO reductase (GSNOR) is an enzyme that acts as a denitrosylase, regulating the protein S‐nitrosylation. Remarkably, depletion of GSNOR in brain cells leads to a significant increase in the aggregation of ubiquitin and α‐syn proteins, which is associated with motor control coordination failure. When mitochondrial depolarization occurs, full‐length PINK1 is localized on the outer mitochondrial membrane to recruit parkin and other autophagic receptors to mitochondria, thereby stimulating mitophagy. Depletion of GSNOR enhances the S‐nitrosylation of parkin in both brain cells and liver cells of mice. Deficiency of GSNOR effectively inhibits parkin function by promoting excessive S‐nitrosylation. Additionally, under conditions of mitophagy induction by the uncoupler CCCP, GSNOR depletion exacerbates mitophagy dysfunction, while wild‐type cells exhibit normal mitophagy function.^437^

### 10.3 Effects of abnormal S‐nitrosylation in HD

Glutamine (Q) is an amino acid that is encoded by CAG and CAA trinucleotides. In certain disorders, such as HD and other poly‐Q diseases, there are expansion mutations in the CAG repeats. Notably, the Htt protein, which is a polyQ‐expanded protein, has been identified as a substrate for S‐nitrosylation. The available evidence suggests that the polyQ expansion in the Htt protein triggers its S‐nitrosylation. Both COS7 and HEK293T cells have demonstrated a significant increase in S‐nitrosylation upon expression of the polyQ‐expanded form (Q128).^440^ Investigation of the N548 fragment and the C‐terminal region of the Htt protein has revealed that the full‐length protein has the potential to undergo S‐nitrosylation. This suggests that multiple cysteine residues in the Htt protein can be S‐nitrosylated, potentially leading to negative modifications in Htt function. Interestingly, polyQ expansion not only stimulates S‐nitrosylation in Htt but also in other polyQ‐containing proteins, such as ataxin‐1. This suggests that the effect of polyQ expansion on S‐nitrosylation may be a general occurrence for proteins with polyQ regions. Considering that palmitoylation of the Htt protein has been reported at cysteine 214 (Cys‐214/C214), this particular residue becomes of interest for evaluation under S‐nitrosylation conditions. The potential interplay between S‐nitrosylation and palmitoylation at Cys‐214 could have implications for the function and pathology of Htt.^440^ Exposure to S‐nitroso (SNO) treatment in cells expressing the N548 wild‐type (normal polyQ) and N548‐C214S mutant forms of Htt has revealed that S‐nitrosylation of Htt is significantly diminished. This suggests that the C214S mutation does not regulate the reduction in S‐nitrosylation. However, while this mutation has a significant effect on the degree of S‐nitrosylation of wild‐type Htt, it does not appear to have a significant impact on N548Q128, the polyQ‐expanded form of Htt. These findings indicate that the presence of Cys‐214 is involved in the S‐nitrosylation of wild‐type Htt, but its role may be different or less pronounced in the context of the polyQ‐expanded form of Htt (N548Q128).^440^ Indeed, studies have reported that expressing mHtt in neuronal cells leads to increased production of NO. Additionally, elevated levels of SNO‐Drp1 have been documented in the striatum of BACHD transgenic mice compared with control groups. It has been established that mHtt has a similar effect on gene expression in the striatum and cerebellum of HD mice, but a lower pathogenicity is observed in the cerebellum. Consistent with these findings, enhanced SNO‐Drp1 has been identified in the striatum, suggesting that it may be a contributing factor to the pathogenicity of HD. These results are not limited to animal models but have also been observed in HD patients, further supporting the association between SNO‐Drp1 and the pathogenicity of HD. Transfection of wild‐type Htt (wtHtt) or mHtt in HEK293‐nNOS cells has revealed that S‐nitrosylation of HTT is higher in mHtt compared with wtHtt. Interestingly, both mHtt and wtHtt are capable of interacting with Drp1, and this interaction is influenced by S‐nitrosylation. However, consistent with the results, it has been documented that there is a lack of Drp1 binding to Htt in the striatum region of control mice. Regarding the interaction of Drp1 with Htt, there is a hypothesis that when Drp1 is bound to mHtt‐SNO, NO might be transferred from mHtt to Drp1. This suggests that S‐nitrosylation of mHtt could potentially facilitate the transfer of NO from mHtt to Drp1 via their interaction. Both NOS inhibitors and S‐nitrosylation‐resistant mutant Drp1 (C644A, Cys^644^ → Ala) are capable of suppressing mitochondrial fragmentation caused by mHtt, suggesting that SNO‐Drp1 has a negative impact on mitochondrial morphology. Based on these results, it appears that mHtt expression provokes NO production, leading to the formation of SNO‐Drp1 and subsequent mitochondrial fragmentation. In HD, a synaptic loss is an early event preceding the neuronal loss. When transfecting cortical neurons with both mHtt (Q148) and wtDrp1, reduced dendritic spine density is observed. Consistently, cotransfection of mutant Drp1 (C644A) or wtDrp1 with the NOS inhibitor N‐nitro‐l‐arginine (NNA) (wtDrp1+NNA) in neuronal cells along with mHtt improves spine density. These findings provide evidence that S‐nitrosylated Drp1 resulting from mHtt not only triggers mitochondrial fission but also contributes to synaptic loss in HD.^441^

## 11 EFFECTS OF ABNORMAL SUCCINYLATION AND NNDs

**FIGURE 12:**
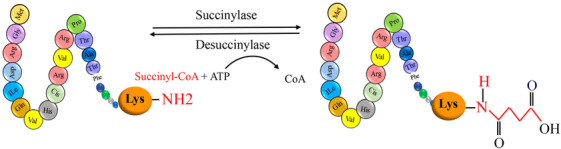
Succinylation is a reversible modification that involves the transfer of a succinyl group (‐CO‐CH_2_‐CH_2_‐CO_2_H) via succinyl‐CoA to a lysine residue of a protein. Succinylases catalyze this modification, which can be removed by desuccinylase enzymes.

Succinylation is a reversible modification that serves as a highly conserved mechanism in various prokaryotes and eukaryotic mitochondria. It entails the transfer of a succinyl group (−CO−CH_2_−CH_2_−CO_2_H) from succinyl‐CoA to a Lys residue on a protein, either through enzymatic or nonenzymatic means. During this succinylation reaction, the positive charge of Lys is counteracted by the incorporation of the succinyl group, leading to the formation of a negatively charged carboxylate group. This modification plays crucial roles in protein folding, function, and various cellular and biological processes, encompassing metabolism, gene transcription, and the DNA damage response (Figure 12). Recent studies have indicated that succinylation has the potential to impact cytosolic, mitochondrial, and nuclear proteins. This includes cellular enzymes that participate in fatty acid synthesis and oxidation, amino acid degradation, mitochondrial respiration, nitrogen metabolism, the urea cycle, and the TCA cycle.^442^ Therefore, succinylation plays a crucial role in the regulation of diverse cellular metabolic pathways. Additionally, the succinylation of histone proteins is believed to participate in the regulation of protein structure and function.

Dysregulation of succinylation has been associated with the development of several diseases, including those related to mitochondrial dysfunction, oxidative stress, cardiovascular disorders, hepatic disorders, pulmonary diseases, cancer, NDDs, and age‐related diseases. Despite its significance, protein succinylation has received relatively less research attention. The interplay between Lys succinylation and acetylation is known to exert notable influences on various metabolic pathways. However, the study of succinylation in the nervous system has been limited, despite its importance in metabolic processes.^443^ Indeed, previous research has provided evidence that Lys succinylation can have functional effects on enzymes involved in energy metabolism. This suggests that succinylation may act as a regulatory mechanism for modulating metabolic pathways in the nervous system. One such enzyme is the KG dehydrogenase complex (KGDHC), which is a multifunctional constituent of mitochondria. Dysregulation of KGDHC has been implicated in various NDDs. The complex consists of several subunits, including KG dehydrogenase (E1k), dihydrolipoamide succinyltransferase (E2k), and dihydrolipoamide dehydrogenase (E3). Indeed, the KG dehydrogenase complex (KGDHC) performs the conversion of KG to succinyl‐CoA, with its E2k subunit being responsible for transferring succinyl groups to target proteins. Interestingly, the evidence suggests that succinylation mediated by the E2k succinyltransferase of KGDHC is more potent compared with succinyl‐CoA. This implies that KGDHC plays a critical role in protein succinylation, which serves as a major signaling system. KGDHC can regulate PTMs of a wide range of proteins located in mitochondria, the cytosol, and the nucleus through its regulation of succinylation. Notably, KGDHC is capable of succinylating neuronal proteins. Loss of KGDHC function has been observed to reduce succinylation of cytosolic and mitochondrial proteins in neuronal cell cultures and cell lines. Decreased KGDHC activity has been reported in various neuronal disorders, including AD, PD, and HD. While the impact of KGDHC on brain function is not yet fully understood, it is known to affect numerous metabolic pathways and consequently influence cell function.^444^ In AD, a decrease in KGDHC activity has been observed, which is associated with the loss of cognitive function. It has been observed that in this condition, the succinylation of mitochondrial proteins is reduced, while the succinylation of cytosolic proteins is increased. This alteration in succinylation patterns suggests a potential translocation of KGDHC from the mitochondria to the cytosol. Additionally, there is evidence indicating that succinylation is involved in plaque and tangle formation, which are characteristic pathological features of AD. However, the specific mechanisms by which succinylation contributes to these processes, its precise implications for the progression of AD, and its effect on brain function are still areas of active research. Further studies are needed to fully elucidate the underlying mechanisms and understand the significance of changes in succinylation patterns in the context of AD. Based on recent studies, it has been determined that aberrant succinylation of Tau protein and APP is associated with the development of AD. Aberrant succinylation refers to abnormal or dysregulated succinylation, which can lead to functional alterations in Tau protein and APP. These modifications can impact their aggregation, processing, and clearance pathways, ultimately contributing to the pathogenesis of AD. However, further studies are needed to clarify the precise role of aberrant succinylation in AD.^444^

Tau protein contains two nucleating six‐residue sequences known as PHF6 (VQIVYK) and PHF6* (VQIINK), both of which are potentially associated with Tau aggregation. The PHF6 sequence is situated at the start of the second repeat (R2) and is only present in four‐repeat Tau isoforms. On the other hand, the PHF6* sequence is located at the beginning of the third repeat (R3) and is found in all Tau isoforms. PTMs of these hexapeptide regions not only have the potential to modify the function of Tau protein but are also associated with the characteristic pathological features of tauopathy diseases. In the brains of patients with AD, but not in control samples, Tau succinylation at the Lys‐311 residue within the PHF6 hexapeptide has been observed. Additionally, in tauopathy mouse models at 4 months of age, an elevated level of Lys succinylation has been detected within Tau oligomers (T22) and phospho‐Tau (AT8). However, at 10 months of age, a decrease in succinylation has been observed in mice. Research indicates that the succinylation of Tau at Lys‐311 significantly impacts the aggregation tendency of the PHF6 region, which is higher compared with PHF6*. Furthermore, studies have indicated that mixtures containing small amounts of succinylated PHF6, along with substantial amounts of unmodified PHF6, promote enhanced PHF6‐induced aggregation. This suggests the crucial role of succinylation in the aggregation of unmodified Tau protein. Interestingly, succinylated PHF6 has been observed to form short filaments resembling the PHFs found in AD, while unmodified PHF6 forms filaments with a longer appearance. In addition, succinylation has been shown to negatively regulate microtubule dynamics by preventing tubulin polymerization. Succinylation leads to the loss of function of normal Tau by suppressing Tau–tubulin interactions. As a result, Tau succinylation hampers microtubule dynamics.^445^

Observations have revealed the succinylation of the Lys‐612 residue in APP samples from patients with AD. Similarly, mouse models of AD have demonstrated an early increase in the rate of Lys succinylation. Similar to Tau protein, succinylation of APP is elevated in AD mouse models at 4 months of age but decreases in mouse models at 10 months of age. This decrease is associated with reduced localization of succinylated APP and Aβ plaque accumulation. Therefore, succinylation appears to be an event that occurs in the early stages of Aβ plaque formation in AD mouse models. It is well established that β‐secretase activity is involved in amyloidogenic processing, leading to the production of Aβ aggregation. Conversely, α‐secretase, involved in nonamyloidogenic processing, inhibits Aβ generation by cleaving the Aβ domain. The Lys‐612 and Leu‐613 site is the cleavage site for α‐secretase, and pathogenic mutations in this region can trigger early‐onset AD. Notably, succinylation of the Lys‐16 residue (corresponding to Lys‐612 in APP) has been shown to induce amyloidogenic processing, resulting in the generation of Aβ by inhibiting α‐secretase activity. The Lys‐16 residue (Lys‐612 in APP) is an important residue for both the aggregation and toxicity of Aβ42. Succinylation of Lys‐16 appears to contribute to the formation of Aβ oligomerization, which is considered the most toxic form of Aβ.^445^

## 12 CHALLENGES IN DEVELOPING THERAPIES TARGETING NDDs

Dysregulation of modifications in proteins can have a significant impact on the pathogenesis and progression of various diseases, including neurodegenerative disorders.^4^ Understanding the specific PTMs involved in these diseases is crucial for the development of potential therapeutic approaches. Currently, MS in combination with various analytical methods, has proven to be the most effective approach for exploring PTMs in complex protein mixtures. This powerful technique allows for the identification and characterization of a wide range of PTMs, including phosphorylation, ubiquitylation, acetylation, methylation, SUMOylation, glycosylation, and ADPR. By utilizing these advanced analytical methods, researchers can unravel the intricate landscape of PTMs in proteins, providing valuable insights into their roles in disease processes.^446^ Indeed, the identification and characterization of PTMs of specific residues in pathological amyloid proteins from various samples, such as brain cells, plasma, and CSF samples of neurodegenerative patients, can provide valuable insights into the underlying molecular mechanisms of these diseases.^447^ MS has emerged as a powerful tool in this regard, allowing for the detection and analysis of PTMs in complex biological samples.^448^ However, studying PTMs with MS does face several challenges. One of the primary challenges is the low abundance of PTM‐modified peptides, which can make their detection and analysis difficult. The random selection of precursor ions for MS analysis, based on their abundance, may result in the underrepresentation or complete omission of low‐abundance PTM‐modified peptides. Moreover, certain PTMs can be labile and may degrade during MS and tandem mass spectrometry analysis. Additionally, the study of multisite PTMs using MS can generate complex data that can be challenging to interpret.^446^, ^448^ However, when combined with other techniques, MS‐based proteomics can enhance our understanding of disease mechanisms and facilitate the development of potential methods for early clinical diagnosis of neurological disorders.^447^ Currently, some therapies employed for NDDs are based on immunotherapies and small molecules that target PTMs, inhibit kinases, and activate phosphatases (Table 5). However, despite extensive research, these treatments have not yielded success. This lack of success can be attributed to certain limitations. The CNS is the most intricate and delicate system in the human body, protected by the blood–brain barrier and the blood–CSF barrier. The primary challenge in treating NDDs lies in delivering drugs from the bloodstream to the CNS. The most significant obstacles are related to a limited understanding of the drug's mechanism of action and its bioavailability within brain cells.^449^ Additionally, there are concerns regarding side effects and off‐target effects on nonspecific receptors and enzymes. These barriers are further compounded by the complex pharmacology of drugs, inappropriate dosages, the volatility index of tested drugs, variations in human genotypes and their diverse responses to multiple drugs, the prolonged latent period of NDDs, and the lack of drug efficacy as the disease progresses.^450^ Moreover, one of the major challenges in treating NDDs is identifying the appropriate molecular target. AD, being the most prevalent NDD, has been the subject of numerous therapeutic approaches. However, the current FDA‐approved therapies for AD do not effectively prevent or reverse neuronal loss, brain atrophy, or progressive cognitive decline. Despite many treatment attempts targeting Aβ in AD, they have proven unsuccessful. As a result, there is now a shift in focus towards the Tau protein.^451^ Specifically, the phosphorylation of Tau, which is implicated in the early progression of the disease, has become a target for various therapeutic strategies, including vaccines. However, there are concerns regarding the safety of vaccines that target specific phosphorylation sites, as these sites may also be present in the brains of healthy individuals. Furthermore, it is worth noting that out of the 45 known phosphorylation sites on Tau, only one or two sites have been investigated thus far for the treatment of AD. Furthermore, selecting the appropriate immunogenic epitopes is a critical strategy. In the past, the N‐terminal region of Tau was used as an epitope for Tau‐based immunotherapy, but it has not demonstrated significant success in phase 2 clinical trials. Recent studies have shifted their focus towards the mid‐region of Tau, such as the MTBR, as a more promising target for immunotherapy.^451^ Moreover, a significant challenge in the strategy targeting specific PTMs of Htt, which are dysregulated in HD mutations, lies in identifying enzymes that specifically modulate these PTMs on Htt. Many enzymes, such as kinases and phosphatases, have multiple substrates, making it necessary to identify PTM‐related signaling systems to develop pharmacological modulators that specifically target PTMs. Further exploration is required to address the existing knowledge gaps in this area. Additionally, there is a dearth of animal models for HD therapies, and designing suitable transgenic models can be challenging since they selectively affect specific regions of the brain.^452^, ^453^ Furthermore, recent studies utilizing surface plasmon resonance and spectroscopy have revealed that PTMs of α‐syn can influence the binding affinity of intrabodies like NbSyn87. NbSyn87, which targets a central region within the C‐terminal domain of α‐syn, has demonstrated neuroprotective effects in both in vitro and in vivo models of PD. For example, while Ser129 phosphorylation has no impact on the NbSyn87–α‐syn interaction, Tyr‐125 leads to a reduced affinity in this interaction. Therefore, prioritizing an understanding of the specific properties of patient‐specific α‐syn pathology becomes crucial.^454^ The study of PTMs of α‐syn protein to uncover their functions in PD and other synucleinopathies faces certain limitations. One major challenge is the scarcity of antibodies capable of recognizing these modifications, along with their low abundance in brain cells. While several antibodies are available for phosphorylated α‐syn at the Ser‐129 position (pSer‐129 α‐syn), only a few antibodies exist for other altered α‐syn species, such as ubiquitinated and truncated forms.^455^ Furthermore, most studies investigating PTMs of α‐syn rely on models that involve overexpression of α‐syn rather than modulating PTMs of endogenous α‐syn. While identifying enzymes that modify α‐syn at specific residues can help elucidate the true role of PTMs in regulating pathology formation by endogenous α‐syn, the varying efficiency of these enzymes and the use of diverse cellular and animal models can lead to conflicting findings.

**TABLE 5:** List of some immunotherapies and small molecules that directly or indirectly regulate PTMs of certain proteins in the context of AD, PD, and HD.

NDDs	Drug	Target	Results	Models	References
AD	AV‐1980R/A with an AdvaxCpG adjuvant	PAD	It decreases hyperphosphorylated Tau at Ser 396 but not other phosphorylated Tau, increases IgG, and improves short‐term memory function.	rTg4510 mice	^456^, ^457^
AD	CP13i and PHF1i intrabodies	Intracellular Tau	Decreases Tau pathology	rTg4510 mice	^457^
AD	scFvs	Extracellular Tau	No therapeutic effect	rTg4510 mice	^457^
AD	ACI‐35 (liposomal vaccine)	pSer396/404 epitope of Tau	It induces antibody formation in mice, and decreases soluble and insoluble Tau protein phosphorylated at S396, however, Tau phosphorylated at s404 is not significantly affected by ACI‐35.	P301L mice	^457^, ^458^
			Safe and immunogenic in mild to moderate AD individuals.	clinical trial phase 1b/2a	^457^
AD	RG7345	Phosphorylated Tau at serine 422	Triggers unfavorable pharmacokinetics, discontinued from clinical development	Clinical trial phase 1	^457^, ^459^
AD	PNT001	Cis P‐Thr231	Safe and well tolerated at different dose levels in healthy volunteers	Clinical trial phase 1	^451^, ^460^
AD	Lu AF87908	Phosphorylated Tau at Ser396	Reduces the ability of Tau to form seed aggregates	rTg4510 mice	^451^, ^459^
			Ongoing, evaluate the safety, tolerability, and pharmacokinetics in both healthy and AD individuals	Clinical trial phase 1	^451^
AD	MK‐8719 (OGA inhibitor)	O‐GlcNAcase	Increases Tau protein glycosylation, reduces Tau pathology and mitigates brain atrophy	rTg4510 mouse	^457^
AD	Sodium selenate	PP2A	Activates PP2A, decreases Tau phosphorylation, restores memory deficits	Animal models of tauopathy	^457^
AD	Memantine (inhibits SET‐mediated PP2A inhibition)	SET	It prevents the inhibitory impact of SET on PP2A, elevating PP2A activity and reversing SET‐induced abnormal Tau phosphorylation.	PC12 cell	^461^
	COG112 (Apolipoprotein E‐mimetic peptide) (inhibits SET and PP2A interaction)	SET	It inhibits SET and PP2A interaction, enhancing PP2A activity and reducing Tau phosphorylation and somatodendritic accumulation of Tau in both hippocampus and cerebral cortex.	AD mouse model	^461^
AD	Salsalate	Acetylated K174	Reduces Tau protein acetylation at K174 and prevents hippocampal atrophy and memory deficits	PS19 mice	^457^
AD	MK‐8719, (OGA inhibitor)	O‐GlcNAcase	Increases Tau protein glycosylation, reduces aberrant Tau protein pathology and mitigates brain atrophy	rTg4510 mouse	^457^
AD	Folic acid	Tau protein	Prevents Tau aggregation, reduces Tau phosphorylation through stabilizing its native state	In vitro experiment	^457^
AD	CLR01	Tau protein	Decreases Tau hyperphosphorylation, aggregation, and oligomerization	P301S‐Tau mice	^457^
AD	Tideglusib (GSK‐3β inhibitor)	GSK‐3 β	There was not any positive therapeutic outcome	Clinical trial phase 2	^457^
AD	Lithium (GSK‐3β inhibitor)	GSK‐3 β	Reduces Tau phosphorylation (Ser202, Ser396/Ser404) and aggregation Reduces Tau phosphorylation in CSF of amnestic MCI patients and improves their cognitive performance Improves cognitive ability of AD patients	AD mouse model double‐blind trial clinical trial	^288^, ^457^ ^457^ ^457^
			ongoing, recruiting patients	Clinical trial phase 3	^457^
PD	Nilotinib (c‐Abl inhibitor)	C‐Abl	Clearance of α‐syn, improvement of motor performances, restoration of DAT, dopamine production in the striatum, and TH expression in the substantia nigra	Preclinical models	^462^
PD	Nilotinib	C‐Abl	Reduces oligomeric α‐syn and phosphorylated Tau, improves dopamine metabolism, and enhances HVA and DOPAC levels in CSF without affecting motor or nonmotor outcomes	Clinical trial	^462^
PD	IkT‐148,009 (c‐Abl inhibitor)	C‐Abl	Reduces p‐tyr39 and p‐ser129 α‐syn, inhibiting neurodegeneration progress	Preclinical models	^462^
			Ongoing, evaluate the safety, tolerability, maximum tolerated dose, and the pharmacokinetic profile in single and multiple doses	clinical trial phase 1	^462^
	Vodobatinib (C‐Abl inhibitor)	C‐Abl	Prevents the protein kinase activity of c‐Abl with a sub‐nanomolar potency, possesses appreciable BBB penetration and promotes autophagic flux. Protects mouse models from nigrostriatal neuronal loss.	Preclinical models	^462^
			Well‐tolerated, selection of two doses	Clinical trial phase 1	^462^
			Recruiting patients with early PD to evaluate the two selected doses	Clinical trial phase 2	^462^
PD	ASN120290 (OGA inhibitor)	O‐GlcNAcase	Increases α‐syn O‐GlcNAcylation and mitigates motor impairment	Preclinical model	^462^
PD	Ginkgolic acid	Not determined E1–SUMO complex	Reduces SUMOylation levels and stimulates the clearance of α‐syn aggregates in an autophagy‐dependent manner Preventing protein Sumoylation via suppressing E1–SUMO complex formation	In vitro experiment Preclinical model	^462^, ^463^ ^464^
HD	N6FFA	Huntingtin N17 domain	Improves phosphorylation of N17, reverses HD phenotypes and reduces huntingtin inclusions in the cortex	HD Mouse model	^8^, ^465^
HD	CP13 (anti‐Tau pS202 monoclonal antibody)	Tau pS202	Reduces hippocampal pS202, improves motor performance, and cognitive function	zQ175 mice	^288^
HD	L807mts (GSK‐3β inhibitor)	GSK‐3 β	Reduces striatal mHtt aggregates, improves motor coordination abilities and enhances neuroprotection	R6/2 mice	^288^
HD	Lithium (GSK‐3β inhibitor)	GSK‐3β	Improves motor performance and alleviates the striatal neuropathological deficits	YAC128 HD mouse model	^280^

Abbreviations: PAD, phosphatase‐activating domain; scFvs, single‐chain variable fragments; PP2A, protein phosphatase 2A; K175, lysine 175; OGA, O‐GlcNAcase; GSK3β, glycogen synthase kinase‐3 beta; CSF, cerebrospinal fluid; MCI, mild cognitive impairment; c‐Abl, c‐Abelson tyrosine kinase; DAT, dopamine transporter; TH, tyrosine hydroxylase; HVA, homovanillic acid; DOPAC, 3,4‐dihydroxyphenylacetic acid; BBB, blood–brain barrier; N6FFA, N6‐furfuryladenine.

## 13 DISCUSSION

There is a growing body of evidence indicating that cross‐talk between PTMs can play a significant role in disease pathogenesis and drug development.^466^ NDDs involve several pathological proteins that serve as substrates for numerous PTMs, suggesting the existence of interprotein PTM crosstalk. This interplay between the proteins and their PTMs may have important implications for understanding disease mechanisms and developing therapeutic interventions.^467^ In AD, pathological forms of Aβ and Tau proteins have traditionally been considered to function independently. However, recent studies suggest an interaction between these proteins that synergistically contributes to brain atrophy and cognitive decline.^451^ Additionally, there is evidence of molecular interaction and cross‐seeding between Aβ and α‐syn, with codeposition of these proteins observed in AD patients. Interestingly, this interaction appears to reduce Aβ accumulation.^253^ Understanding the intricate interactions between these proteins, particularly their PTMs, is crucial for the development of effective therapeutic strategies. The interprotein PTM cross‐talk among these proteins can influence their structure and function, leading to both antagonistic and synergistic effects. Additionally, it is important to note that intraprotein PTM crosstalk, involving modifications occurring on the same protein, may synergistically promote the aggregation of pathological proteins.^467^ In AD, Tau undergoes various PTMs that are associated with Tau aggregation. Abnormal phosphorylation of Tau is known to play a crucial role in the promotion of Tau tangles, a hallmark of AD pathology. Additionally, Lys methylation of specific sites on Tau, particularly within the MTBR and projection domain, has been reported to be involved in Tau tangle formation. However, it is worth noting that Lys methylation may also represent a physiological modification that exerts a neuroprotective role against Tau aggregation.^415^, ^468^ Moreover, acetylation and ubiquitination are involved in the regulation of Tau accumulation and mislocalization. Ubiquitination is associated with PHFs of Tau in AD tissues, inhibiting Tau‐mediated microtubule assembly, and hyperubiquitination exacerbates Tau accumulation.^469^ Importantly, in AD patients, there is a potential interplay between Tau ubiquitination and phosphorylation, as phosphorylation influences Tau ubiquitination, indicating a potential impact on the structure of Tau filaments.^340^ On the other hand, studies have also documented that deubiquitination of Tau contributes to its aggregation and delays its degradation. Additionally, the acetylation of the Lys‐280 residue of Tau triggers its phosphorylation. However, acetylation of Tau on the Lys‐259, Lys‐290, Lys‐321, and Lys‐353 residues prevents Tau phosphorylation.^469^ Hence, it is essential to identify the cross‐talk between PTMs of a protein, considering the wide variety and combinations of PTMs observed in NDDs. Furthermore, there may be competition between PTMs targeting a specific residue. Taking Tau as an example, the Lys residues of Tau are prone to various PTMs, such as SUMOylation, acetylation, ubiquitination, methylation, and glycation. Each of these modifications has its distinct effects on Tau's structure, function, and aggregation. It has been suggested that Lys‐based PTMs may inhibit Tau ubiquitination, consequently impairing its degradation. For instance, the Lys‐254 residue of Tau serves as a substrate for both ubiquitination and methylation. However, in fibrillar Tau, Lys‐254 is found to be more heavily methylated than ubiquitinated. This observation suggests that the methylation of Lys‐254 may suppress Tau degradation mediated by the UPS, ultimately leading to increased levels of Tau. Similarly, acetylation can also hinder Lys ubiquitination, resulting in inadequate turnover of endogenous Tau.^470^ Taken together, the documented involvement of abnormal PTMs in NDDs highlights the importance of further exploring the PTMs of specific proteins. Investigating the cross‐talk between these PTMs, their impact on protein interactions, and the competition between PTMs targeting specific residues can offer valuable insights into disease mechanisms and facilitate the development of targeted therapies. Such research could significantly contribute to a better understanding of NDDs and the identification of potential therapeutic interventions.

Numerous proteins expressed abundantly in the brain have crucial roles in neuronal function, undergoing various PTMs. This article provides a review of the key proteins associated with neurodegenerative disorders, including Aβ, Tau, and α‐syn, which are involved in important NDDs, and discusses their abnormal PTMs, with a particular focus on SUMOylation. PTMs, such as SUMOylation, phosphorylation, acetylation, methylation, ubiquitylation, and others, play a significant role in neuronal function and are altered in NDDs. These changes can lead to protein malfunction, aggregation, and accumulation, which are hallmark features of NDDs like AD. For instance, SUMOylation is a complex process that involves an intricate enzymatic cascade and various covalent and noncovalent interactions. It plays a crucial role in regulating protein function and cellular processes. A significant number of proteins have been identified as substrates for SUMO modification. Dysregulation of protein SUMOylation has been associated with a wide range of diseases. Protein SUMOylation is involved in the regulation of neuroprotection, plasticity, and synaptic transmission.^129^ Any dysregulation in this process has the potential to lead to the accumulation of key proteins associated with NDDs, thereby interfering with essential neurological functions.^16^ Studies have demonstrated that SUMOylation plays a dual role, both potentially exacerbating and safeguarding against protein aggregation. Consequently, SUMOylation has been implicated in various NDDs, including AD and PD. For example, the involvement of α‐syn SUMOylation and associated ubiquitin‐ligases in the pathogenesis of PD highlights their crucial role in disease progression. Substantial evidence suggests that SUMOylation plays a significant role in the pathological aggregation of α‐syn and the formation of LBs, which contribute to the development of inclusion formations in α‐synucleinopathies.^111^, ^114^ Moreover, as mentioned earlier, various abnormal PTMs have been identified in different NDDs. These PTMs, including SUMOylation, phosphorylation, ubiquitination, and methylation, can occur on the same protein and significantly contribute to the progression of NDDs. A prominent example is the Tau protein, which is strongly implicated in numerous NDDs. Multiple PTMs have been observed within different domains of the Tau protein. Consequently, in NDDs, the presence of abnormal PTMs, particularly phosphorylation at specific sites, has been shown to play a critical role in the pathogenesis of these diseases, notably in AD.^225^

The intricate interplay among diverse PTMs, including sumoylation, phosphorylation, O‐GlcNAcylation, and ubiquitination, plays a vital role in governing various physiological processes and influences the onset and progression of NDDs.^75^ Consequently, the cross‐talk between these PTMs plays a critical and pivotal role in the progression of NDDs. These PTMs on proteins not only individually modify their functions but also exert direct and indirect influences on each other. For instance, the intricate interplay of diverse PTMs on Tau plays a pivotal role in these diseases, particularly in AD. These PTMs have a multifaceted influence on Tau, affecting its functional roles, abnormal aggregation, and cytotoxicity. Therefore, understanding the complex relationship between these PTMs on Tau is essential for comprehending the pathogenesis of AD and other related NDDs.^471^ When Tau undergoes hyperphosphorylation, its propensity for SUMOylation is increased, resulting in decreased solubility and ubiquitination, and ultimately impeding Tau degradation. Hyperphosphorylation of Tau triggers a conformational change and initiates a decrease in its affinity for microtubules, leading to the release of soluble Tau. While p‐Tau denotes soluble hyperphosphorylated Tau, PHFs‐Tau is insoluble.^211^, ^228^ Purified Tau proteins exhibit high solubility and heat resistance. The characteristics displayed by free‐soluble Tau suggest the presence of potential cross‐talk interactions with ubiquitination and phosphorylation pathways. Additionally, SUMOylation may selectively target the free soluble pool of the substrate, underscoring the significance of these interactions and the implications of SUMOylation for Tau's pathogenic roles in NDDs.^28^, ^50^ These findings provide insights into the mechanisms underlying Tau accumulation in AD.

Furthermore, Tau undergoes O‐GlcNAcylation at specific serine and threonine residues. Elevated levels of O‐GlcNAcylation have the potential to regulate Tau's functions by preventing hyperphosphorylation. Consequently, this promotes Tau degradation and suppresses its aggregation.^471^ Furthermore, compelling studies have shed light on the influence of phosphorylation and O‐GlcNAcylation on the APP's serine and threonine residues, supporting the cross‐talk between these PTMs. These intricate modifications have been shown to intricately impact APP processing and the generation of Aβ peptides.^75^ Currently, there are no proven methods to halt or slow down the progression of NDDs such as AD, PD, and HD.^171^, ^472^ However, targeting PTMs to treat NDDs remains an expanding area of research and development, offering promising avenues for innovative therapeutic interventions. For instance, the intricate interplay of signaling pathways plays a crucial role in regulating protein phosphorylation by balancing the activities of protein kinases and PPs. This interplay provides a potential avenue for therapeutic regulation.^242^ In the context of tauopathies, the role of PP2A activation in modulating Tau phosphorylation has emerged as a significant area of research, offering new possibilities for AD treatment. Potential drugs such as folate, metformin, and ceramides have shown promise in targeting PP2A. Reduced PP2A activity leads to increased phosphorylation levels, promoting hyperphosphorylation of Tau and exacerbating Alzheimer's symptoms. Furthermore, PP2A plays a crucial role in dephosphorylating and deactivating kinases, in addition to its role in Tau phosphatase activity.^473^ The increasing understanding of PTMs and NDDs has driven research into targeted protein degradation (TPD) as a potential therapeutic strategy and chemical tool. Remarkable advancements have been achieved in TPD technologies, particularly in the development of proteolysis‐targeting chimeras. These innovative approaches have garnered significant attention for their potential applications in various NDDs, including HD, AD, and PD. By selectively targeting the degradation of disease‐associated proteins such as mHtt, Tau, Aβ, and α‐syn, these approaches offer promising prospects for the treatment of NDDs.^474^, ^475^

Modifying the PTMs of key proteins involved in various cellular pathways can have profound effects on their functions, potentially leading to cognitive deficits and other symptoms associated with NDDs. To gain a comprehensive understanding of the interplay between PTMs and ND‐associated proteins, precise identification of target proteins and the implications of PTMs in the context of NDDs is essential. While the importance of PTMs in the pathogenesis of NDDs is evident, the exact mechanisms and the extent of their cross‐talk and influence are still subjects of debate and require further investigation. Advancements in this field have the potential to unveil novel therapeutic targets and strategies for the treatment of NDDs, providing hope for improved outcomes for affected individuals.

## AUTHOR CONTRIBUTIONS

S. R. and M. DA. were responsible for conducting literature searches related to the topic of the article, while S. R., M. DA., M. DR., and N. S. contributed to the writing of the article itself. S. R. was responsible for organizing the structure and framing of the manuscript and also created all of the figures included in the article. A. A. and S. R. edited the final version of the article. Finally, all authors read and approved the final manuscript, indicating their agreement with the content and readiness for publication.

## CONFLICT OF INTEREST STATEMENT

The authors declare no conflict of interest.

## FUNDING INFORMATION

Not applicable.

## ETHICS STATEMENT

Not applicable.

## Data Availability

Not applicable.
